# Design, Synthesis, Antidiabetic Activity and *In Silico* Studies of New Hydrazone Derivatives Derived from Acetohexamide

**DOI:** 10.1021/acsomega.5c04642

**Published:** 2025-09-19

**Authors:** Bedriye Seda Kurşun Aktar, Yusuf Sıcak, Emine Elçin Oruç-Emre, Rabia Kılıç, Ebru Sağlam, Demet Taşdemir, Süleyman Kaya, Gizem Tatar Yılmaz, Ayse Sahin Yaglioglu

**Affiliations:** † Department of Hair Care and Beauty Services, Yeşilyurt Vocational School, Malatya Turgut Özal University, 44210 Battalgazi, Malatya, Türkiye; ‡ Department of Medicinal and Aromatic Plants, Köyceğiz Vocational School, Mugla Sitki Kocman University, 48800 Muğla, Türkiye; § Department of Chemistry, Faculty of Arts and Sciences, Gaziantep University, 27310 Gaziantep, Türkiye; ∥ Respiratory Diseases and Respiratory Surgery Research and Practice Center, Gaziantep University, 27310 Gaziantep, Türkiye; ⊥ Department of Medical Biochemistry, Faculty of Medicine, Gaziantep University, 27310 Gaziantep, Türkiye; # Department of Biostatistics and Medical Informatics, Faculty of Medicine, 52976 Karadeniz Technical University, 61080 Trabzon, Türkiye; ∇ Institute of Health Sciences, Department of Bioinformatics, Karadeniz Technical University, 61080 Trabzon, Türkiye; ○ Yılmaz Bilişim R&D Consulting Software Engineering and Services Trade Limited Company, 61081 Trabzon, Türkiye; ◆ Department of Chemistry and Chemical Process Technology, Technical Sciences Vocational School, Amasya University, 05100 Amasya, Türkiye

## Abstract

*Diabetes mellitus* affects over 500 million people globally and is expected to rise significantly in the coming decades. Existing antidiabetic drugs, including α-glucosidase and α-amylase inhibitors, often exhibit side effects and limited efficacy, prompting the search for safer alternatives. Hydrazone derivatives have shown promising antidiabetic activity due to their structural diversity and enzyme-targeting potential. In this study, 10 novel hydrazone compounds were synthesized and evaluated for their inhibitory effects against α-amylase and α-glucosidase. Compounds **8** and **10** showed the highest dual inhibition: compound **8** with IC_50_ = 30.21 ± 0.16 μM (α-amylase) and 38.06 ± 0.80 μM (α-glucosidase); compound **10** with IC_50_ = 34.49 ± 0.37 and 40.44 ± 0.23 μM, respectively. Cytotoxicity on HEK293 cells via MTT assay revealed IC_50_ values of 61.04 μM (compound **7**) and 69.25 μM (compound **9**), while other compounds and acarbose were nontoxic up to 100 μM. In silico drug-likeness analysis showed that 80% of the compounds complied with Lipinski’s rules, with topological polar surface area (TPSA) values ranging between 63 and 112 Å^2^. Gastrointestinal absorption was high for 7 out of 10 compounds; none showed blood–brain barrier permeability. Molecular docking confirmed strong binding interactions of compounds **8** and **10** with both enzymes’ active sites. These findings highlight hydrazone scaffolds as potent and safe candidates for further antidiabetic drug development.

## Graphical abstract

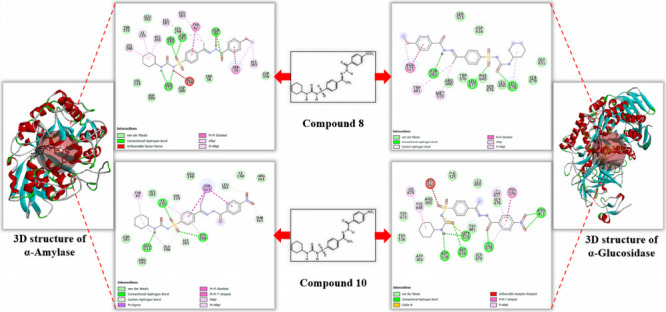

## 1 Introduction

*Diabetes mellitus* is a chronic endocrine disorder characterized by persistent hyperglycemia due to defects in insulin secretion, insulin action, or both.^1,2^ The prevalence of diabetes has increased dramatically worldwide, resulting in significant morbidity, mortality, and economic burden.^1^ Currently available oral antidiabetic drugs, including α-glucosidase and α-amylase inhibitors, aim to delay carbohydrate digestion and glucose absorption, thus reducing postprandial blood glucose levels.^3,4^ However, these drugs often cause adverse effects such as gastrointestinal discomfort and have limited efficacy, which underscores the urgent need for novel therapeutics with improved safety and potency.^5^

Rational drug design has emerged as a powerful approach in the development of new antidiabetic agents, focusing on the molecular optimization of bioactive scaffolds to enhance target specificity, bioavailability, and minimize toxicity.^6,7^ Within this framework, hydrazone derivatives have attracted significant interest due to their structural versatility and wide range of biological activities, including notable antidiabetic effects.^8,9^ The characteristic azomethine (−C=N–NH−) group of hydrazones facilitates diverse chemical modifications, enabling fine-tuning of their pharmacodynamic and pharmacokinetic profiles.^10,11^

Recent studies have demonstrated that hydrazone derivatives can effectively inhibit α-glucosidase and α-amylase enzymes, key targets in diabetes management that regulate carbohydrate metabolism.^12,13^ Inhibiting these enzymes slows down glucose release and absorption, thereby controlling postprandial hyperglycemia.^3,4^ The design of hydrazone-based inhibitors often incorporates strategic substitutions, such as heterocyclic rings and various electron-donating or withdrawing groups, to optimize enzyme binding affinity and selectivity, as confirmed by structure–activity relationship (SAR) studies.^14,15^ Furthermore, integration of molecular docking simulations with in vitro enzymatic assays provides mechanistic insights into ligand-enzyme interactions, facilitating the rational design of more effective and selective antidiabetic agents.^16,17^ In this context, the choice of crystal structures for molecular docking plays a critical role in accurately modeling enzyme–ligand interactions. Therefore, well-resolved structures such as human α-glucosidase (e.g., PDB ID: 3TOP) and porcine pancreatic α-amylase (e.g., PDB ID: 1OSE) were selected, based on previous studies demonstrating their structural reliability and relevance for inhibitor docking. For example, Nawaz et al. employed 3TOP to model α-glucosidase–inhibitor interactions in a study involving 5-amino-nicotinic acid derivatives and validated their results through redocking and SAR analysis.^18^ Similarly, Rosa et al. recently employed 3TOP in a comprehensive metabolomics–machine-learning–docking study of *Artabotrys sumatranus* leaf extract, showing strong binding of predicted α-glucosidase inhibitors to the human enzyme model.^19^ For α-amylase the use of 1OSE is well-documented; **for instance, Timalsina et al. performed in vitro and in silico analyses of Catunaregam spinosa extracts using porcine pancreatic α-amylase (PDB ID: 1OSE), identifying key interactions with catalytic residues such as Glu233 and Asp300.^20^ These examples support our selection of these PDB IDs for subsequent MD and MM/PBSA simulations.

Despite these promising findings, challenges remain in developing hydrazone derivatives with strong inhibitory activity and low cytotoxicity. This study aims to address these challenges by synthesizing a series of novel hydrazone compounds guided by rational design principles. Their inhibitory effects on α-glucosidase and α-amylase were evaluated, alongside cytotoxicity assessments in human embryonic kidney cells (HEK293). Molecular docking analyses further elucidated binding modes and affinities, contributing to the understanding of their potential as safer and more potent antidiabetic candidates.

## 2 Experimental Section

### 2.1 Materials and Methods

All chemicals and solvents were analytical grade, purchased from Acros, Alfa Aesar, Sigma-Aldrich and Merck in purity suitable for synthesis or analytical grade. Chemical reactions were monitored using thin layer chromatography (TLC, Merck 60 F_254_). Melting points were determined by EZ-Melt MPA120 Automated Melting Point Apparatus and were uncorrected. FTIR spectra were recorded on Cary 630 FTIR Spectrometer and PerkinElmer 1620 model Frontier spectrometer by attenuated total reflectance (ATR) apparatus (Waltham, Massachusetts, USA). Nuclear magnetic resonance spectra (NMR) of compounds were determined on the Bruker spectrometer (400 MHz), the TMS was used as an external reference and reported in parts per million. Analyzes of C, H, N, S percentages of the original synthesized compounds were made using the Thermo Scientific Flash 2000 Organic Elemental Analyzer device. Mass analyzes were performed by ionization method on LC-MS/MS Agilent Technologies 1260 Infinity II, 6460 Triple Quad Mass Spectrometer device. The ionization method in mass spectrometry was Electrospray ionization (ES). Antidiabetic inhibitory activities were carried out on a 96-well microplate reader, SpectraMax 340PC^384^, Molecular Devices (USA). Spectroscopic data of compounds **1**–**14** were given in Supporting Information.

### 2.2 General Procedure for the Synthesis of *N*-(Cyclohexylcarbamoyl)-4-(1-(2-(substitutedbenzoyl)­hydrazinylidene)­ethyl)­benzenesulfonamide (**1**–**14**)

Acetohexamide (1.0 mol) was dissolved in acetonitrile, 2–3 drops of glacial acetic acid and substituted hydrazides (1.0 mol) were added and heated under reflux. The reaction was checked with TLC at regular intervals and when the reaction was completed, it was left to cool. The resulting product was filtered, dried and purified by washing with ethanol.^21,22^

#### 2.2.1 4-(1-(2-Benzoylhydrazinilidene)­ethyl)-*N*-(cyclohexylcarbamoyl)­benzenesulfonamide (**1**)

White solid (65%); mp 215–217 °C; FT-IR: 3347 (N–H stretching band); 3045 (aromatic C–H stretching band); 2937, 2855 (aliphatic C–H stretching band); 1654 (C=O stretching band); 1550 (C=N stretching band); 1483, 1397 (aromatic ring C=C stretching band); 1338 (asymmetric SO_2_ stretching band); 1159 (symmetric SO_2_ stretching band). ^1^H NMR (DMSO-*d*_6_, 600 MHz), δ (ppm): 1.1–1.23 (m, 5H); 1.49 (d, 1H, *J* = 17.20 Hz); 1.56–1.77 (m, 4H); 2.41 (s, 3H); 3.38 (inside the water peak 1H); 6.33 (s, 1H); 7.49–7.61 (m, 5H); 7.88 (s, 2H); 8.03 (s, 2H); 10.90 (s, 1H). ^13^C NMR (DMSO-*d*_6_, 150 MHz) δ (ppm): 20.41, 22.94, 25.17, 26.68, 32.05, 47.43, 126.54, 127.16, 127.28, 127.70, 128.71, 128.73, 132.60, 139.73, 144.80, 149.93, 160.70. Elemental Analysis: C_22_H_26_N_4_O_4_S (442,53 g/mol). Anal. Calcd (%): C, 59.71; H, 5.92; N, 12.66; S, 7.24. Found (%): C, 59.75; H, 5.97; N, 12.69; S, 7.28. LC-MS (*m*/*z*): 443.1 [M]^+^

#### 2.2.2 4-(1-(2-(3-Chlorobenzoyl)­hydrazinylidene)­ethyl)-*N*-(cyclohexylcarbamoyl)­benzenesulfonamide (**2**)

White solid (17%); mp 212–214 °C; FT-IR: 3339, 3220 (N–H stretching band); 3186 3034 (aromatic C–H stretching band); 2933, 2858 (aliphatic C–H stretching band); 1651 (C=O stretching band); 1535 (C=N stretching band); 1453, 1394, 1334 (aromatic ring C=C stretching band); 1334 (asymmetric SO_2_ stretching band); 1162 (symmetric SO_2_ stretching band).^1^H NMR (DMSO-*d*_6_, 600 MHz), δ (ppm): 1.08–1.26 (m, 5H); 1.48–1.68 (m, 5H); 2.45 (s, 3H), 3.30 (s, 1H); 6.40 (d, 1H, *J* = 7.6 Hz); 7.57 (t, 1H, *J*_1_ = 8.0 Hz, *J*_2_ = 7.6 Hz); 7.70 (d, 1H, *J* = 7.6 Hz); 7.85–8.13 (m, 6H); 10.43 (s, 1H); 11.01 (s, 1H). ^13^C NMR (DMSO-*d*_6_, 150 MHz) δ (ppm): 15.17, 24.67, 25.45, 32.74, 48.61, 126.20, 127.34, 127.83, 128.22, 130.79, 131.90, 133.55, 136.33, 141.08, 142.73, 150.92, 154.31, 163.26. Elemental Analysis: C_22_H_25_ClN_4_O_4_S (476.98 g/mol). Anal. Calcd (%): C, 55.40; H, 5.28; N, 11.75; S, 6.72. Found (%): C, 55.43; H, 5.32; N, 11.80; S, 6.76. LC-MS (*m*/*z*): 475.0 [M-H]^−^

#### 2.2.3 *N*-(Cyclohexylcarbamoyl)-4-(1-(2-(4-fluorobenzoyl)­hydrazinylidene)­ethyl)­benzenesulfonamide (**3**)

White solid (56%); mp 142–143 °C; FT-IR: 3287 (N–H stretching band); 3068 (aromatic C–H stretching band); 2929, 2851 (aliphatic C–H stretching band); 1637 (C=O stretching band); 1550 (C=N stretching band); 1472, 1439, 1362 (aromatic ring C=C stretching band); 1343 (asymmetric SO_2_ stretching band); 1154 (symmetric SO_2_ stretching band). ^1^H NMR (DMSO-*d*_6_, 600 MHz), δ (ppm): 1.07–1.23 (m, 5H); 1.49 (d, 1H, *J* = 8.8 Hz); 1.58–1.65 (m, 4H); 2.41 (s, 3H); 2.96 (s, 1H); 6.37 (s, 1H); 7.34–7.44 (m, 2H); 7.91–7.99 (m, 6H); 10.92 (s, 1H). ^13^C NMR (DMSO-*d*_6_, 150 MHz) δ (ppm): 15.01, 24.22, 24.75, 25.51, 32.86, 49.81, 115.58, 115.80, 126.21, 127.12, 127.71, 130.88, 141.60, 142.37, 152.02, 165.73. Elemental Analysis: C_22_H_25_FN_4_O_4_S (460,52 g/mol). Anal. Calcd (%): C, 57.38; H, 5.47; N, 12.17; S, 6.96. Found (%): C, 57.43; H, 5.51; N, 12.20; S, 6.99. LC-MS (*m*/*z*): 461.1 [M]^+^

#### 2.2.4 *N*-(Cyclohexylcarbamoyl)-4-(1-(2-(4-iodobenzoyl)­hydrazinylidene)­ethyl)­benzenesulfonamide (**4**)

White solid (30%); mp 244–245 °C; FT-IR: 3331 (N–H stretching band); 3072 (aromatic C–H stretching band); 2936, 2859 (aliphatic C–H stretching band); 1647 (C=O stretching band); 1536 (C=N stretching band); 1492, 1478, 1450 (aromatic ring C=C stretching band); 1335 (asymmetric SO_2_ stretching band); 1162 (symmetric SO_2_ stretching band). ^1^H NMR (DMSO-*d*_6_, 600 MHz), δ (ppm): 1.06–1.21 (m, 5H); 1.44–1.63 (m, 5H); 2.47 (s, 3H); 3.25 (s, 1H); 6.36 (s, 1H); 7.65 (s, 2H), 7.89 (s, 4H); 8.01 (s, 2H); 10.38 (s, 1H); 10.91 (s, 1H). ^13^C NMR (DMSO- *d*_6_, 150 MHz) δ (ppm): 15.06, 24.62, 25.41, 32.69, 48.57, 99.78, 127.28, 127.81, 130.38, 133.69, 137.61, 140.95, 142.75, 150.83, 154.00, 164.00. Elemental Analysis: C_22_H_25_IN_4_O_4_S (568.43 g/mol). Anal. Calcd (%): C, 46.49; H, 4.43; N, 9.86; S, 5.64. Found (%): C, 46.52; H, 4.49; N, 9.89; S, 5.67. LC-MS (*m*/*z*): 569.1 [M]^+^.

#### 2.2.5 *N*-(Cyclohexylcarbamoyl)-4-(1-(2-(4-bromobenzoyl)­hydrazinylidene)­ethyl)­benzenesulfonamide (**5**)

White solid (78%); mp 259–260 °C; FT-IR: 3339 (N–H stretching band); 3004 (aromatic C–H stretching band); 1754, 1600 (C=O stretching band); 1526 (C=N stretching band); 1455, 1427 (aromatic ring C=C stretching band); 1344 (asymmetric SO_2_ stretching band); 1160 (symmetric SO_2_ stretching band). ^1^H NMR (DMSO-*d*_6_, 600 MHz), δ (ppm): 1.08–1.27 (m, 5H); 1.49 (d, 1H, *J* = 12.0 Hz); 1.58–1.67 (m, 4H); 2.64 (s, 3H); 6.39 (d, 1H, *J* = 8.40 Hz); 7.75 (d, 2H, *J* = 8.40 Hz); 7.86 (s, 2H); 8.02 (d, 2H, *J* = 8.80 Hz); 8.13 (d, 2H, *J* = 8.80 Hz); 10.60 (s, 1H). ^13^C NMR (DMSO-*d*_6_, 150 MHz) δ (ppm): 24.68, 25.43, 27.54, 32.71, 48.68, 126.21, 128.06, 129.19, 130.02, 132.11, 140.38, 144.38, 150.91, 165.44, 197.86. Elemental Analysis: C_22_H_25_BrN_4_O_4_S (521.43 g/mol). Anal. Calcd (%): C, 50.68; H, 4.83; N, 10.75; S, 6.15. Found (%): C, 50.71; H, 4.86; N, 10.81; S, 6.20.

#### 2.2.6 4-(1-(2-([1,1-Biphenyl]-4-carbonyl)­hydrazilidene)­ethyl)-*N*-(cyclohexylcarbomoyl)­benzenesulfonamide (**6**)

White solid (52%); mp 269–270 °C; FT-IR: 3346 (N–H stretching band); 3053 (aromatic C–H stretching band); 2934, 2858 (aliphatic C–H stretching band); 1654 (C=O stretching band); 1531 (C=N stretching band); 1449, 1396 (aromatic ring C=C stretching band); 1342 (asymmetric SO_2_ stretching band); 1164 (symmetric SO_2_ stretching band). ^1^H NMR (DMSO-*d*_6_, 600 MHz), δ (ppm): 1.06–1.21 (m, 5H); 1.45 (d, 1H, *J* = 9.0 Hz); 1.54–1.63 (m, 4H); 2.43 (s, 3H); 3.26 (d, 1H, *J* = 7.2 Hz); 6.36 (s, 1H); 7.39 (t, 1H, *J*_1_ = 7.8 Hz, *J*_2_ = 7.2 Hz); 7.48 (t, 2H, *J*_1_ = 7.8 Hz, *J*_2_ = 7.2 Hz); 7.72 (d, 2H, *J* = 7.2 Hz); 7.79 (d, 2H, *J* = 8.4 Hz); 7.91–8.02 (m, 5H); 10.39 (s, 1H); 10.93 (s, 1H). ^13^C NMR (DMSO-*d*_6_, 150 MHz) δ (ppm): 14.96, 24.62, 25.41, 32.70, 48.57, 126.94, 127.24, 127.35, 127.81, 128.58, 129.19, 129.51, 133.12, 139.61, 140.95, 142.83, 143.66, 150.91, 153.55, 164.26. Elemental Analysis: C_28_H_30_N_4_O_4_S (518.63 g/mol) Anal. Calcd (%): C, 64.85; H, 5.83; N, 10.80; S, 6.18 Found (%): C, 64.88; H, 5.86; N, 10.85; S, 6.23.

#### 2.2.7 *N*-(Cyclohexylcarbamoyl)-4-(1-(2-(4-methylbenzoyl)­hydrazinylidene)­ethyl)­benzenesulfonamide (**7**)

White solid (30%); mp 228–229 °C; FT-IR: 3339 (N–H stretching band); 3053 (aromatic C–H stretching band); 2934, 2858 (aliphatic C–H stretching band); 1654 (C=O stretching band); 1531 (C=N stretching band); 1484, 1449, 1396 (aromatic ring C=C stretching band); 1342 (asymmetric SO_2_ stretching band); 1164 (symmetric SO_2_ stretching band). ^1^H NMR (DMSO-*d*_6_, 600 MHz), δ (ppm): 1.06–1.11 (m, 3H); 1.15–1.21 (m, 2H); 1.45 (d, 1H, *J* = 12.60 Hz); 1.55 (d, 2H, *J* = 13.8 Hz); 1.62 (d, 2H, *J* = 12.6 Hz); 2.36 (d, 3H, *J* = 7.8 Hz), 2.47 (s, 2H); 3.25 (d, 1H, *J* = 7.2 Hz); 6.35 (d, 1H, *J* = 8.4 Hz); 7.30 (d, 2H, *J* = 7.8 Hz); 7.77 (s, 2H); 7.90 (d, 2H, *J* = 7.8 Hz); 7.98 (d, 2H, *J* = 8.4 Hz); 10.38 (s, 1H); 10.78 (s, 1H). ^13^C NMR (DMSO-*d*_6_, 150 MHz) δ (ppm): 14.84, 21.48, 26.62, 25.40, 32.68, 39.54, 39.68, 39.82, 39.96, 40.10, 40.24, 40.38, 48.56, 127.19, 127.79, 128.03, 128.64, 129.17, 131.44, 140.82, 142.90, 150.82, 153.04, 164.25. Elemental Analysis: C_23_H_28_N_4_O_4_S (456.56 g/mol). Anal. Calcd (%): C, 60.51; H, 6.18; N, 12.27; S, 7.02. Found (%): C, 60.54; H, 6.23; N, 12.34; S, 7.07. LC-MS (*m*/*z*): 457.1 [M]^+^.

#### 2.2.8 *N*-(Cyclohexylcarbamoyl)-4-(1-(2-(4-methoxy)­hydrazinilidene)­ethyl)­benzenesulfonamide (**8**)

White solid (30%); mp 233–234 °C; FT-IR: 3338 (N–H stretching band); 3268 (aromatic C–H stretching band); 3017 (aliphatic C–H stretching band); 1654 (C=O stretching band); 1575 (C=N stretching band); 1504, 1486, 1458 (aromatic ring C=C stretching band); 1336 (asymmetric SO_2_ stretching band); 1160 (symmetric SO_2_ stretching band). ^1^H NMR (DMSO-*d*_6_, 600 MHz), δ (ppm): 1.10–1.23 (m, 5H); 1.49 (d, 1H, *J* = 12.60 Hz); 1.55–1.66 (m, 4H); 2.65 (s, 3H), 3.26 (s, 1H); 3.83 (s, 3H); 6.46 (s, 1H); 7.05 (d, 2H, *J* = 6.00 Hz); 7.92 (d, 2H, *J* = 5.00 Hz); 8.03 (d, 2H, *J* = 5.60 Hz); 8.14 (d, 2H, *J* = 5.60 Hz); 10.56 (s, 1H). Elemental Analysis: C_23_H_28_N_4_O_5_S (472.56 g/mol). Anal. Calcd (%): C, 58.46; H, 5.97; N, 11.86; S, 6.78. Found (%): C, 58.49; H, 6.00; N, 11.89; S, 6.83.

#### 2.2.9 *N*-(Cyclohexylcarbamoyl)-4-(1-(2-(3-nitrobenzoyl)­hydrazinylidene)­ethyl)­benzenesulfonamide (**9**)

White solid (72%); mp 292–293 °C; FT-IR: 3346 (N–H stretching band); 2852 (aromatic C–H stretching band); 2085 (aliphatic C–H); 1614 (C=O stretching band); 1538 (C=N stretching band); 1452, 1393, 1233 (aromatic ring C=C stretching band); 1393 (asymmetric SO_2_ stretching band); 1096 (symmetric SO_2_ stretching band). ^1^H NMR (DMSO-*d*_6_, 600 MHz), δ (ppm): 1.10–1.24 (m, 5H,); 1.49 (d, 1H, *J* = 13.6 Hz); 1.59–1.65 (m, 4H); 2.45 (s, 3H); 3.36 (inside the water peak, 1H); 6.42 (s, 1H) 7.84 (t, 1H, *J*_1_ = 7.20 Hz, J_2_ = 5.6 Hz), 8.08 (d, 2H, *J* = 8.0 Hz); 8.13 (d, 2H, *J* = 8.0 Hz); 8.35 (d, 1H, *J* = 7.20 Hz); 8.44 (s, 1H); 8.69 (s, 1H); 10.69 (s, 1H); 11.22 (s, 1H). Elemental Analysis: C_22_H_25_N_5_O_6_S (487.53 g/mol). Anal. Calcd (%): C, 54.20; H, 5.17; N, 14.37; S, 6.58. Found (%): C, 54.23; H, 5.19; N, 14.43; S, 6.63.

#### 2.2.10 *N*-(Cyclohexylcarbamoyl)-4-(1-(2-(4-nitrobenzoyl)­hydrazinylidene)­ethyl)­benzenesulfonamide (**10**)

White solid (49%); mp 210–211 °C; FT-IR: 3319 (N–H stretching band); 3070 (aromatic C–H stretching band); 2931, 2855 (aliphatic C–H); 1716, 1661 (C=O stretching band); 1520 (C=N stretching band); 1482, 1402 (C=C stretching band of aromatic ring); 1337 (asymmetric SO_2_ stretching band); 1161 (symmetric SO_2_ stretching band). ^1^H NMR (DMSO-*d*_6_, 600 MHz), δ (ppm): 1.05–1.20 (m, 5H); 1.44 (m, 5H); 2.40 (s, 3H); 3.27 (s, 1H); 6.35 (s, 1H), 7.92 (s, 2H), 8.03 (s, 1H); 8.10 (s, 2H); 8.32 (s, 2H,); 10.38 (s, 1H); 11.18 (s, 1H). ^13^C NMR (DMSO-*d*_6_, 150 MHz) δ (ppm): 15.26, 24.62, 25.40, 32.68, 48.57, 123.88, 126.19, 127.43, 127.83, 128.11, 141.35, 142.58, 149.63, 150.82, 154.95, 163.19. Elemental Analysis: C_22_H_25_N_5_O_6_S (487.53 g/mol). Anal. Calcd (%): C, 54.20; H, 5.17; N, 14.37; S, 6.58. Found (%): C, 54.23; H, 5.21; N, 14.41; S, 6.62.

#### 2.2.11 4-(1-(2-(1-Naphthalene)­hydrazinylidene)­ethyl)-*N*-(cyclocarbamoyl)­benzenesulfonamide (**11**)

White solid (53%); mp 232–233 °C; FT-IR: 3332, 3209, (N–H stretching band); 3045, 3179 (aromatic C–H stretching band); 2933, 2858 (aliphatic C–H stretching band); 1647 (C=O stretching band); 1524, 1453, 1394 (aromatic ring C=C stretching band); 1334 (asymmetric SO_2_ stretching band); 1162 (symmetric SO_2_ stretching band). ^1^H NMR (DMSO-*d*_6_, 600 MHz), δ (ppm): 1.09–1.20 (m, 5H); 1.44–1.64 (m, 5H); 2.36 (s, 3H); 3.35 (s, 1H); 6.38 (d, 1H, *J* = 7.8 Hz); 7.52–7.61 (m, 4H); 7.77 (d, 1H, *J* = 6.60 Hz); 7.98 (d, 2H, *J* = 8.80 Hz); 8.07 (t, 2H, *J*_1_ = 9.00 Hz, *J*_2_ = 8.40 Hz); 8.19 (d, 1H, *J* = 9.60 Hz); 10.38 (s, 1H); 11.43 (s, 1H). ^13^C NMR (DMSO-*d*_6_, 150 MHz) δ (ppm): 15.01, 24.62, 25.41, 32.70, 48.59, 118.47, 125.45, 125.59, 126.20, 126.61, 126.79, 127.36, 127.82, 128.75, 130.76, 133.59, 140.94, 142.95, 150.87, 152.66, 166.01, 172.30. Elemental Analysis: C_26_H_28_N_4_O_4_S (492.59 g/mol). Anal. Calcd (%): C, 63.40; H, 5.73; N, 11.37; S, 6.51. Found (%): C, 63.46; H, 5.78; N, 11.43; S, 6.57. LC-MS (*m*/*z*): 490.9 [M-H]^−^

#### 2.2.12 4-(1-(2-(2-Naphthalene)­hydrazinylidene)­ethyl)-*N*-(cyclocarbamoyl)­benzenesulfonamide (**12**)

White solid (61%); mp 154–157 °C; FT-IR: 3332 (N–H stretching band); 3041 (aromatic C–H stretching band); 2933 (aliphatic C–H stretching band); 1651 (C=O); 1524, 1453 (aromatic ring C=C stretching band); 1334 (SO_2_ asymmetric stretching band); 1162 (symmetric SO_2_ stretching band). ^1^H NMR (DMSO-*d*_6_, 600 MHz), δ (ppm): 1.07–1.26 (m, 5H); 1.49 (d, 1H, *J* = 13.20 Hz); 1.59–1.66 (m, 4H); 2.47 (s, 3H); 3.28 (s, 1H); 6.38 (d, 1H, *J* = 7.60 Hz); 7.61–7.68 (m, 2H); 7.95–8.12 (m, 9H); 8.54 (s, 1H); 11.07 (s, 1H). ^13^C NMR (DMSO-*d*_6_, 150 MHz) δ (ppm): 15.02, 24.20, 24.70, 25.47, 30.83, 32.79, 48.60, 126.21, 127.27, 127.80, 128.14, 128.30, 129.41, 132.51, 134.76, 141.67, 142.72, 145.28, 149.07, 151.33, 161.68, 164.49. Elemental Analysis: C_26_H_28_N_4_O_4_S (492.59 g/mol). Anal. Calcd (%): C, 63.40; H, 5.73; N, 11.37; S, 6.51. Found (%): C, 63.45; H, 5.79; N, 11.41; S, 6.55 LC-MS (*m*/*z*): 491.1 [M-H]^−^.

#### 2.2.13 *N*-(Cyclohexylcarbamoyl)-4-(1-(2-(2-nitrobenzoyl)­hydrazinylidene)­ethyl)­benzenesulfonamide (**13**)

White solid (61%); mp 154–157 °C; FT-IR: 3307 (N–H stretching band); 3236 (aromatic C–H stretching band); 2934, 2851 (aliphatic C–H stretching band); 1687 (C=O); 1593 (C=N stretching band); 1528, 1449 (aromatic ring C=C stretching band); 1338 (SO_2_ asymmetric stretching band); 1165 (symmetric SO_2_ stretching band).^1^H NMR (DMSO-*d*_6_, 600 MHz), δ (ppm): 1.10–1.23 (m, 5H); 1.47–1.65 (m, 5H); 2.30 (s, 3H); 3.37 (s, 1H); 6.37 (s, 1H) 7.74 (d, 2H, *J* = 7.20 Hz), 7.88 (t, 2H, *J*_1_ = 7.60, *J*_2_ = 7.60 Hz); 7.96 (d, 1H, *J* = 8.40 Hz); 8.09–8.12 (m, 3H); 10.97 (s, 1H). ^13^C NMR (DMSO-*d*_6_, 150 MHz) δ (ppm): 15.46, 22.24, 24.18, 25.43, 32.74, 49.78, 124.63, 124.81, 126.04, 127.53, 127.69, 127.91, 130.11, 130.59, 132.03, 134.26, 147.66, 164.97. Elemental Analysis: C_22_H_25_N_5_O_6_S (487.53 g/mol). Anal. Calcd (%): C, 54.20; H, 5.17; N, 14.37; S, 6.58. Found (%): C, C, 54.25; H, 5.20; N, 14.41; S, 6.65. LC-MS (*m*/*z*): 488.1 [M]^+^.

#### 2.2.14 4-(1-(2-(4-Chlorobenzoyl)­hydrazinylidene)­ethyl)-*N*-(cyclohexylcarbamoyl)­benzenesulfonamide (**14**)

White solid (61%); mp 212–214 °C; FT-IR: 3325 (N–H stretching band); 3096 (aromatic C–H stretching band); 2942, 2851 (aliphatic C–H stretching band); 1650 (C=O); 1528 (C=N stretching band), 1521, 1448, 1397 (aromatic ring C=C stretching band); 1338 (SO_2_ asymmetric stretching band); 1162 (symmetric SO_2_ stretching band). ^1^H NMR (DMSO-*d*_6_, 600 MHz), δ (ppm): 1.10–1.26 (m, 5H); 1.49 (d, 1H, *J* = 12.80 Hz); 1.58–1.67 (m, 4H); 2.41 (s, 3H); 6.40 (d, 1H, *J* = 8.40 Hz); 7.60 (d, 4H, *J* = 8.40 Hz,); 7.87–8.04 (m, 4H); 10.64 (s, 1H); 10.95 (s, 1H). ^13^C NMR (DMSO-*d*_6_, 150 MHz) δ (ppm): 15.46, 22.24, 24.18, 25.43, 32.74, 49.77 (Cyclohegzyl C), 124.63, 124.81, 126.26, 127.53, 127.69, 130.10, 130.58, 132.02, 134.26 (Ar–C), 147.66 (C16), 164.97 (C=O). Elemental Analysis: C_22_H_25_ClN_4_O_4_S (476,98 g/mol) Anal. Calcd (%): C, 55.40; H, 5.28; N, 11.75; S, 6.72. Found (%): C, 55.43; H, 5.34; N, 11.78; S, 6.75.

### 2.3 Determination of Antidiabetic Inhibition Activity

The compounds (**1**–**14**) in all biological activity studies were dissolved in dimethyl sulfoxide (DMSO) and DMSO was used as a negative control.

#### 2.3.1 Determination of α-Amylase Inhibitory Activity of Compounds **1**–**14**

α-Amylase inhibitory activity of compound **1**–**14** was tested by using the spectroscopic method with slight changes.^23^ Briefly, 25 μL sample solution in different concentrations (12,5–25–50–100 μM) and 50 μL α-amylase solution (0.1 U/mL) in phosphate buffer (20 mM pH = 6.9 phosphate buffer prepared with 6 mM NaCl) were mixed in a 96-well microplate. The mixture was preincubated for 10 min at 37 °C. After preincubation, 50 μL starch solution (0.05%) was added and incubated for more 10 min at 37 °C. The reaction was stopped by addition of 25 μL HCl (0.1 M) and then 100 μL Lugol solutions were added for monitoring. 96-well microplate reader was used to measure absorbance at 565 nm.

#### 2.3.2 Determination of α-Glucosidase Inhibitory Activity of Compounds **1**–**14**

α-Glucosidase inhibitory activity of compound **1**–**14** was determined using the spectroscopic method with slight modifications.^24^ Briefly, 50 μL phosphate buffer (10 mM pH = 6.9), 25 μL *p*-nitrophenyl-α-d-glucopyranoside in phosphate buffer (10 mM pH = 6.9), 10 μL sample solution in different concentrations (12,5–25–50–100 μM) and 25 μL α-glucosidase (0.1 U/mL) in phosphate buffer (10 mM pH = 6.0) were mixed in a 96-well microplate. After 20 min incubation at 37 °C, 90 μL Na_2_CO_3_ (100 mM) was added into the each well to stop the enzymatic reaction. Absorbance of the 96-well microplate reader was recorded at 400 nm.

#### 2.3.3 Determination of PPAR-Gamma Activity of Compounds **1**–**14**

The activity of PPARγ was assessed by ELISA using PPAR-gamma Ligand Screening/Characterization Assay Kit (Abcam, Cambridge, UK). Prepared a reaction mixture that included 1 μL of samples from various concentrations (100, 50, 25, 12.5 μM/mL) of newly synthesized chemicals, 24 μL of fluorescent probe, and PPAR-gamma recombinant. The prepared reaction mixture was transferred to a 384-well plate designed for fluorescence reading. Fluorescence was measured every 10 min for 60 min using the Thermo Variscan at excitation/emission wavelengths of 375/460–470 nm. The kit included the control ligand WY-14643, which was manufactured at doses of 10.000, 1000, 100, 50, 25, and 12.5 μM. The positive control was WY-14643.

### 2.4 Cell Culture And Chemical Exposure

The HEK293 was used for cell culture studies and obtained from the American Type Culture Collection (ATCC). Cells were cultured in RPMI-1640 cell medium supplemented with 10% fetal calf serum (FBS) and 1% penicillin/streptomycin. Cells were seeded 6 × 10^5^/mL density into 96 well cell culture plates and incubated in a CO_2_ incubator at 37 °C. The cells were exposed to different chemical concentrations (100, 50, 25 and 12,5 μM) for 24 h after reaching a confluency of 80%.

### 2.5 Assessment of Toxicity

The MTT assay was used to assess the cell viability. The MTT assay included putting the cells in an MTT solution (at 1 mg/mL concentration) and incubating them for 1 h at 37 °C. The absorbance was measured at a wavelength of 570 nm using an Epoch microplate spectrophotometer.^25^

### 2.6 Molecular Docking

Molecular docking is a computational technique to predict how a ligand (e.g., a small molecule, peptide, or drug candidate) binds to a specific target molecule, usually a protein or enzyme. By simulating molecular interactions at the atomic level, molecular docking provides valuable insights into the structure–function relationships of biomolecules, aiding in the rational design of therapeutics.^26^ In this study, 14 designed compounds were subjected to molecular docking analyses to investigate their interactions with α-amylase and α-glucosidase enzymes. The main objective was to investigate the binding affinities of these compounds for both enzymes and their binding interactions at the molecular level.

The crystal structures of α-amylase (PDB ID: 4W93)^27^ and α-glucosidase (PDB ID: 5NN4)^28^ were retrieved from the Protein Data Bank (https://www.rcsb.org/) and selected based on their high resolution and prior use in the literature for studying enzyme–ligand interactions.^29–32^ Before docking, water molecules were removed from both structures, and the protonation states were adjusted to physiological conditions (pH 7) using the PDB 2PQR software.^33^ The binding regions of the enzymes were determined using the AGFR1.2 software.^34^ For α-amylase, the Cartesian coordinates of the grid box center were set at *x* = −11.712, *y* = 3.609, and *z* = −23.324, with a grid box size of 76 × 62 × 56 Å and a grid spacing of 0.375 Å. Similarly, for α-glucosidase, the center coordinates were set at *x* = −12.941, *y* = −29.032, and *z* = 97.326, with a grid box size of 62 × 68 × 66 Å and a grid spacing of 0.375 Å. A grid parameter file (.gpf) was generated based on these parameters to facilitate the docking process. The Lamarckian Genetic Algorithm (LGA), a hybrid optimization method, was employed to identify the most favorable binding conformations between the ligands and enzymes. The docking simulations were carried out using 2,500,000 score evaluations and 27,000 generations, with 100 docking runs conducted for each compound. The docking parameter file (.dpf) necessary for these simulations was generated using AutoDock 4.2 software,^35^ and all calculations were performed using the same platform.

The docking results were analyzed using scoring functions to assess the binding energies between the ligands and the enzymes. The ligands with the highest binding affinities for the enzymes’ active sites, determined by the lowest binding energies, were identified. Subsequently, the two-dimensional structures of the resulting enzyme-ligand complexes were modeled, and the interactions (including hydrogen bonds, van der Waals forces, and electrostatic interactions) between the ligands and the amino acids in the active sites of the target enzymes were examined.

### 2.7 Molecular Dynamics (MD) Simulations and MM/PBSA Free Energy Analyses

Based on molecular docking and experimental studies involving α-amylase and α-glucosidase enzymes, compounds **8** and **10** were identified as the most promising candidates, demonstrating high binding affinities and significant biological activities. To further assess the structural stability, binding interactions, and energy profiles of the enzyme-ligand complexes formed by these compounds with both enzymes, molecular dynamics (MD) simulations were performed using the GROMACS software package.^36^ The enzyme models were prepared with the CHARMM36 force field and the TIP3P water model,^37^ while ligand topology parameter files were generated using the CGenFF server.^38^ Simulation systems were constructed within a dodecahedral box under periodic boundary conditions, and sodium ions were added to neutralize the overall charge. After system preparation, MD simulations were conducted in three stages: energy minimization, equilibration, and production.

During the initial energy minimization stage, 1000 steps of the steepest descent algorithm were applied to resolve steric clashes and achieve optimal geometric configurations. Following this, equilibration was performed in two phases: first, stabilizing the system under an isothermal-isochoric (NVT) ensemble, followed by equilibration under an isothermal–isobaric (NPT) ensemble, with each phase lasting 100 ps. The final production stage involved a 100 ns simulation using a 2 fs time step. Throughout the simulations, the structural behaviors of the complexes were rigorously analyzed using metrics such as root-mean-square deviation (RMSD), root-mean-square fluctuation (RMSF), and radius of gyration (Rg).

Binding free energy calculations were performed using the Molecular Mechanics Poisson–Boltzmann Surface Area (MM/PBSA) method, employing the GROMACS-compatible g_mmpbsa tool.^57^ The binding free energy (Δ*G*_bind_) of the protein–ligand complexes was calculated using the following equations:

$$\Delta G_{\mathrm{bind}}=E_{\mathrm{vdW}}+E_{\mathrm{elec}}+G_{\mathrm{polar}}+G_{\mathrm{nonpolar}}$$

In this equation, the binding free energy (Δ*G*_bind_) is determined through the individual contributions of van der Waals interaction energy (*E*_vdW_), electrostatic interaction energy (*E*_elec_), polar solvation energy (*G*_polar_), and nonpolar solvation energy (*G*_nonpolar_), all of which are calculated separately.

### 2.8 In Silico ADME and Toxicity Analysis

Computational studies of the synthesized compounds **1**–**14** were performed to predict molecular properties using the SwissADME online server.^39^ The molecular volume (Mv), molecular weight (Mw), logarithm of partition coefficient (mi log *P*), number of hydrogen-bond donors (HBDs), number of hydrogen-bond acceptors (HBAs), topological polar surface area (TPSA), number of rotatable bonds (Nrotbs), and Lipinski’s rule of five of the synthesized compounds were determined. Osiris Property Explorer is a knowledge-based activity prediction tool that predicts undesired properties such as mutagenic, tumorigenic, irritant, and reproductive effects of novel compounds based on chemical fragment data of available drugs and nondrugs as reported.^40^ Protox-II incorporates molecular similarity, fragment propensities, most frequent features and (fragment similarity based CLUSTER cross-validation) machine-learning, based on a total of 61 models for the prediction of toxicity end points such as acute toxicity, organ toxicity, toxicological end points, molecular initiating events, metabolism, adverse outcomes (Tox21) pathways and toxicity targets.^41^ PASS Online, a software developed by the Russian Institute of Biomedical Chemistry (IBMC) and freely accessible by browsers, predicts the biological activities of compounds based on the structural formulas of synthesized drug-like synthetic organic compounds and provides data.^42^

### 2.9 Statistical Analysis

Antidiabetic inhibition activity data were taken in three parallel measurements for four concentrations of each synthesis sample. The results of the biological activity analyses are presented as IC_50_ values. Data were recorded as mean ± SEM (standard error of the mean) *p* < 0.01.

## 3 Result and Discussion

### 3.1 Chemistry

This study is the first research in which the in vitro antidiabetic inhibition activities of synthesized compounds **1–14** were studied. Scheme 1 shows the synthetic pathway and substituent groups (**1**–**14**) carried.

**Scheme 1:**
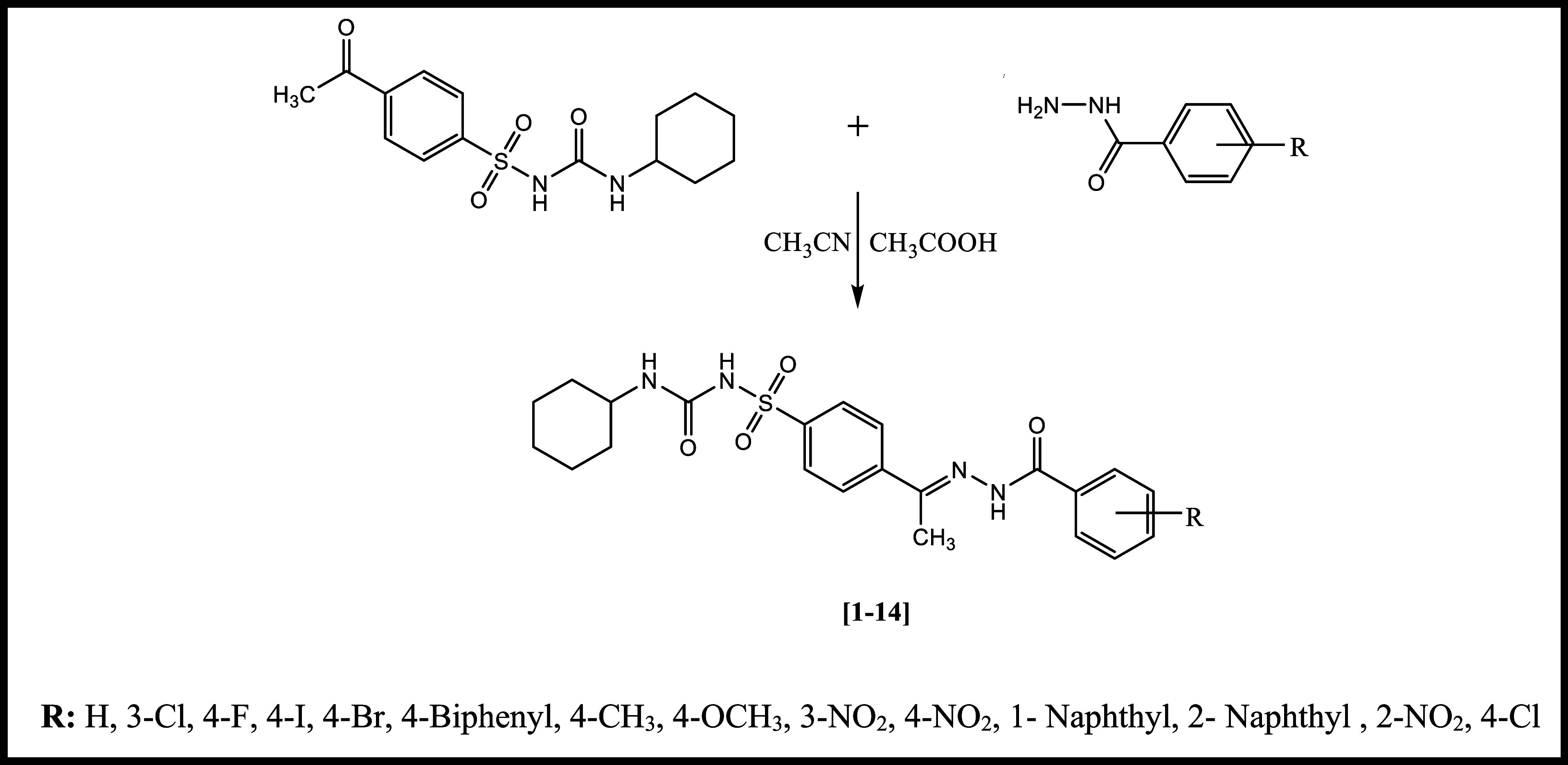
Synthetic Pathway of *N*-(Cyclohexylcarbamoyl)-4-(1-(2-(substitutedbenzoyl)­hydrazinylidene)­ethyl)­benzenesulfonamides (**1–14**)

In the FT-IR spectra were examined, it was determined that the N–H bands were in the range of 3287–3346 cm^–1^; aromatic C–H stretching bands were in the range of 3004–3239 cm^–1^; C=C stretching bands were in the range of 1233–1492 cm^–1^; C=O stretching bands were in the range of 1600–1754 cm^–1^; C=N stretching bands were in the range of 1520–1575 cm^–1^; SO_2_ asymmetric and symmetric stretching bands were in the range of 1272–1393 cm^–1^; 1096–1165 cm^–1^, respectively. The literature values of N–H, C=O and C=N stretching bands are 3223–3347 cm^–1^; 1600–1798 cm^–1^, and 1510–1600 cm^–1^, respectively, and are observed to be consistent with the values in our study.^43–45^ The C=O stretching bands (1600–1754 cm^–1^) of the synthesized hydrazone derivative compounds are in accordance with the C=O stretching bands (1600–1798 cm^–1^) reported in the literature, indicating the correctness of the structures of the synthesized hydrazone derivatives. When the ^1^H NMR spectra were examined, it was found that there are two types of protons in the structure of the cyclohexyl ring and they are positioned as equatorial and axial. It is stated that when a substituent is attached to the cyclohexane ring, the substituent generally prefers the equatorial position.^46^ In this project, the cyclohexane ring coming from acetohexamide used as a starting point preferred the chair structure in the hydrazone compound as in chalcone and pyrazole. The NH protons (10.56–11.43 ppm) belonging to the hydrazone structure and the carbons belonging to C=N were the strongest evidence that hydrazone compounds were synthesized. In the literature, it was found that the N–H peaks of the hydrazone scaffold resonated at 10–17–10.96 ppm, and the N–H peak values of the hydrazones we synthesized were found to be within this range.^47^ The CH_3_ protons resonated in the range of 2.30–2.65 ppm. In the ^13^C NMR spectra are examined, the carbon atoms belonging to C=O resonate in the range of 147.66–166.01/160.70–172.30 ppm, respectively, and the carbon atom belonging to the C=N resonated in the range of 142.37–152.66 ppm. The literature values of C=N resonance peaks were 141.12–159.00 ppm, and it was determined that they were compatible with the values of the synthesized compounds.^48^

As a result of the ES ionization of compound **11**, the C_16_H_21_N_4_O_4_S fragment with a mass of *m*/*z* 365 was observed at the negative ionization fragmentation, and the C_10_H_7_ fragment with a mass of *m*/*z* 127 was observed to gain a proton in the positive ionization fragmentation and to emerge as the *m*/*z* 128 fragment (Table 1).

**Table 1:**
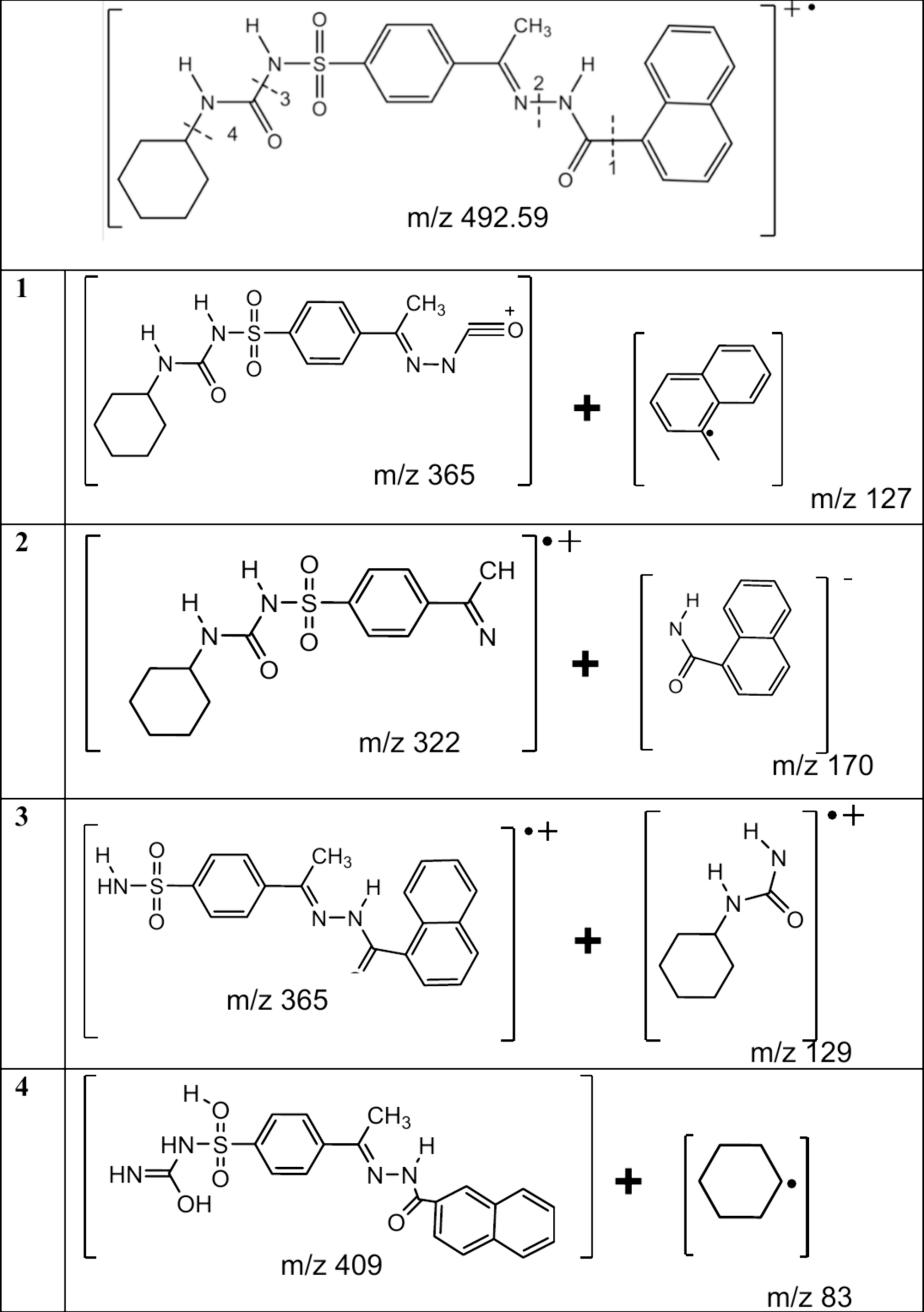
Mass Fragmentation of Compound **11**

As a result of the negative ionization, the fragmentation product of compound **11**, the C_15_H_2_0N_3_O_3_S fragment with a mass of *m*/*z* 322 was observed to give an M^+1^ peak due to the sulfur isotope atom in its structure and to emerge as the *m*/*z* 323 mass. The other product of negative ionization, the C_11_H_8_NO fragment, was observed to have a mass of *m*/*z* 170.9 in the negative ionization fragmentation and these values are shown in the path number 2.

Compound **11**, as a result of fragmentation number 3, has undergone negative ionization fragmentation and the mass of the C_19_H_16_N_3_O_3_S fragment has been found to be *m*/*z* 366, the other product of the fragmentation, fragment C_7_H_12_NO, has given a peak of mass *m*/*z* 126 and as a result of positive ionization fragmentation, it has taken two protons and its mass has been found to be *m*/*z* 128. In negative ionization fragmentation, it has been observed that the C_20_H_17_N_4_O_4_S, the product of fragmentation number 4, has given a peak of M^–1^ with a mass of *m*/*z* 409 and its mass has been found to be *m*/*z* 408.

### 3.2 Pharmacological Activities

#### 3.2.1 Antidiabetic Inhibition Activity and Kinetic Results

The IC_50_ values of antidiabetic inhibition activities of compounds **1**–**14** are given in Table 2. According to activity results, when the overall antidiabetic inhibition activity was tested against both enzymes, it was found that almost all substances exhibited IC_50_ values at <100 μM. Among the molecules in α-amylase inhibition activity, compounds **8** (IC_50_ = 30.21 ± 0.16 μM) and **10** (IC_50_ = 34.49 ± 0.37 μM) exhibited the best activity; in terms of α-glucosidase activity, all synthesized compounds except **1**, **4**, and **6** were determined to be more active than acarbose (IC_50_ = 80.78 ± 0.25 μM), which was used as the positive standard. The most effective on α-glucosidase activity and those with the best IC_50_ were found to be compound **8** (IC_50_ = 38.06 ± 0.80 μM); **10** (IC_50_ = 40.44 ± 0.23 μM); it was found to be compound **3** (IC_50_ = 45.34 ± 0.36 μM) and **7** (IC_50_ = 46.14 ± 0.33 μM). Among the compounds that were tested α-amylase and α-glucosidase inhibition activities, compounds **8** and **10** were exhibited significant activity against both α-amylase and α-glucosidase enzymes (Figures 1 and 2).

**Table 2:** Antidiabetic Inhibition Activities of Compounds **1**–**14**

	antidiabetic inhibition activity IC_50_ (μM)	
compound	α-amylase	α-glucosidase
**1** (H)	86.07 ± 0.12	108.05 ± 0.69^a^
**2** (3-CI)	53.98 ± 0.61	68.75 ± 0.37
**3** (4-F)	39.73 ± 0.55	45.34 ± 0.36
**4** (4-I)	69.43 ± 0.19	84.97 ± 0.46
**5** (4-Br)	60.51 ± 0.80	77.36 ± 0.31
**6** (biphenyl)	78.05 ± 0.14	96.68 ± 0.28
**7** (4-CH_3_)	41.40 ± 0.26	46.14 ± 0.33
**8** (4-OCH_3_)	30.21 ± 0.16	38.06 ± 0.80
**9** (3-NO_2_)	51.26 ± 0.41	61.18 ± 0.69
**10** (4-NO_2_)	34.49 ± 0.37	40.44 ± 0.23
**11** (1-naphthyl)	48.27 ± 0.39	57.06 ± 0.78
**12** (2-naphthyl)	45.26 ± 0.10	50.39 ± 0.11
**13** (2-NO_2_)	52.75 ± 0.60	66.38 ± 0.37
**14** (4-CI)	43.09 ± 0.72	52.25 ± 0.44
**acarbose** ^b^	35.18 ± 0.73	80.78 ± 0.25

a Values expressed are the mean ± SEM of three parallel measurements (*p* < 0.05).

b Reference compounds.

**Figure 1:**
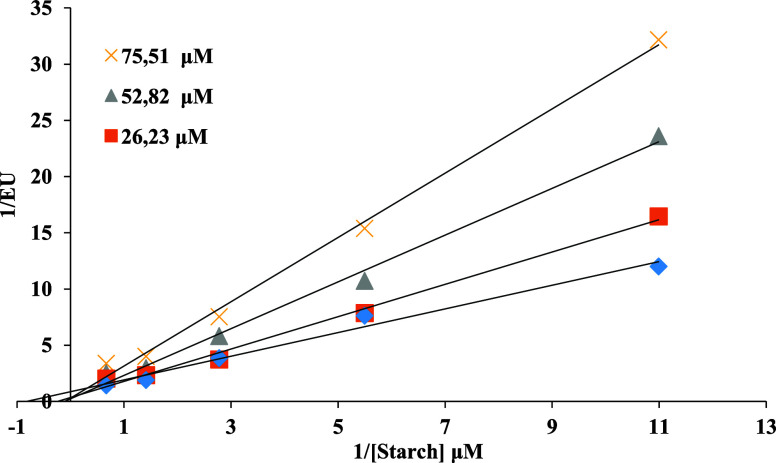
EnzyLineweaver–Burk plot of the inhibition kinetics of α-amylase by compound **8**.

**Figure 2:**
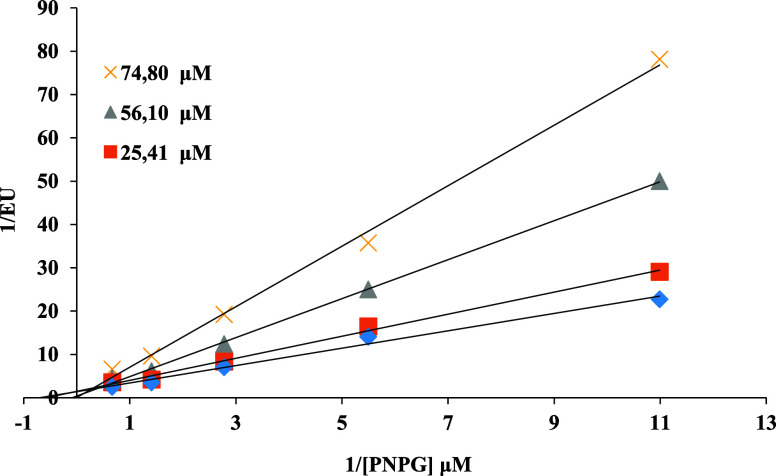
EnzyLineweaver–Burk plot of the inhibition kinetics of α-glucosidase by compound **8**.

#### 3.2.2 Structure–Activity Relationships (SAR)

The aldehyde group of acetohexamide is coupled with hydrazine/hydrazides, bioactive hydrazone compounds are obtained. The bioactivity of hydrazones is considered to be one of the most interesting structural entities in medicinal chemistry due to the ability of the azomethine scaffold to interact with the target protein of the enzyme through noncovalent interactions by acting as a hydrogen bond acceptor. From this point of view, it is observed from the activity results that the new hydrazone scaffolds based on acetohexamide inhibit α-amylase and α-glucosidase to different degrees. The different degrees of inhibition of enzymes by these synthetic derivatives can be explained by the electronic effect of the substituents in the synthetic molecules and the positioning of these substituents. The substituents in the synthetic derivatives contain electron withdrawing and electron donating groups.

The IC_50_ values of compounds **3** (IC_50_ = 45.34 ± 0.36 μM), **4** (IC_50_ = 84.97 ± 0.46 μM), **5** (IC_50_ = 77.36 ± 0.31 μM), and **14** (IC_50_ = 43.09 ± 0.72 μM) containing halogen groups (F, Cl, Br, I) attached to the fourth position of the phenyl ring of were examined, it was determined that α-amylase and α-glucosidase inhibition activities decreased as the atomic diameter increased. It was determined that compound **3**, carrying F atom, was the molecule with the lowest IC_50_ value among the halogen-bearing compounds (**4**, **5,** and **14**).

The IC_50_ values of compound **7**, which had a methyl group attached to the fourth position of the phenyl ring, and compound **8**, which hada methoxy group attached, were determined as 46.14 ± 0.33 and 38.06 ± 0.80 μM, respectively. These two compounds were compared in terms of activity, due to the electronegativity of the oxygen atom, the methoxy group was a group that provides electrons to the ring by resonance compared to the methyl group, in this case it was thought to have a positive contribution to the activity by activating the bond. It was observed that compound **8** had the best activity.

Nitro-substitution is included in the structure of drugs such as Azomycin, Nifurtimox, Benznidazole, Tinidazole, Fexinidazole, Ventetoclax, Delamanid, Entacapone, etc. In addition, it is also found in the structure of therapeutic agents containing a nitro group with bioreductive potential, such as Paclitaxel, Tarlocotinib, BTZ043, Evofosfamide, CB-1954 and KS119,^49^ which are known as prodrugs. However, the nitro group is considered both a pharmacophore and a toxicophore (hepatotoxic, mutagenic). In this study, -NO_2_, an electron-withdrawing group, was bound to the phenyl ring at different positions, the IC_50_ values were 61.18 ± 0.69 μM for compound **9**, 40.44 ± 0.23 μM for compound **10** and 66.38 ± 0.37 μM for compound **13**. It was observed that the enzyme inhibition activity for compound **10**, which nitro group was connected to the phenyl ring at fourth position, was better compared to compound **13**, which nitro group was connected to second position, and compound **9**, which nitro group was connected to third position. Compounds containing only a nonsubstituted phenyl ring in the structure were examined, phenyl rings were bonded in compound **1**, biphenyl rings in compound **6**, 1-naphthalene rings in compound **11** and 2-naphthalene rings in compound **12**, and electrophilic positioning in naphthalene occurs more easily than in benzene. Among these compounds, compound **12** (IC_50_ = 50.39 ± 0.11 μM), which carried a 2-naphthalene ring, showed the best inhibition activity in this series, while compound **11** (IC_50_ = 57.06 ± 0.78 μM), compound **6** (IC_50_ = 96.68 ± 0.28 μM) and compound **1** (IC_50_ = 108.05 ± 0.69 μM). It was observed that compound **6**, to which the biphenyl ring was attached, gived higher inhibition activity compared to compound **1**, to which the unsubstituted phenyl ring is attached. When all the synthesized compounds were examined, the compounds that were active against α-glucosidase were compounds **2**, **3**, **5**, **7**, **8**, **9**, **10**, **11**, **12**, and **13,** respectively. The most effective compound against α-glucosidase was determined to be compound **8**.

Compound **2** and **14** were examined for the Cl atom bonded to the phenyl ring at different positions, the α-amylase inhibition activity of compound **14**, which the Cl atom was bonded at fourth position, was IC_50_ = 43.09 ± 0.72 μM and the α-glucosidase inhibition activity of this compound was IC_50_ = 52.25 ± 0.44 μM. It was observed that α-amylase inhibition activity IC_50_ = 53.98 ± 0.61 μM and α-glucosidase inhibition activity IC_50_ = 68.75 ± 0.37 μM for compound **2**, to which the Cl atom was bonded at third position. In the α-amylase inhibition activity of these two compounds, it was observed that the inhibition activity value of compound **14**, which was attached to the ring at the fourth position, had a better activity than compound **2**, which the Cl atom was attached to the ring at the third position. When the α-glucosidase inhibition activities were examined, it was observed that compound **4** had a better inhibition activity than compound **2**, and it was determined that both compounds had a better α-glucosidase inhibition activity than acarbose.

A nitro group can generate large charge density by withdrawing electrons from the aromatic ring via resonance effect, while the compound halogen substituent can shift the electron cloud via an inductive effect. It has been observed that the moderate electron withdrawing methoxy group inhibits both enzymes better than the strong electron donating nitro group. Based on this, it is shown that the novel hydrazone scaffolds based on acetohexamide containing strong electron withdrawing groups in the scaffold can seriously inhibit these two enzymes. In conclusion, novel hydrazone scaffolds based on acetohexamide showed significant inhibition on α-amylase and α-glucosidase and can serve as lead molecules in the design of DM inhibitors.

### 3.3 PPAR-Gamma Ligand Activation Analysis

In our study, we did not assess PPARγ activations, which may also contribute to the beneficial effects of newly synthesized compounds.

### 3.4 In Vitro Cytotoxic Analysis

All of compounds were evaluated cytotoxic effect by the MTT test on the HEK293 cell line. All IC_50_ values are shown in Table 3. Compounds **7** and **9** have been observed to kill half of the healthy HEK293 cells (***p < 0.001 vs DMSO, IC_50_:61.04 μM, and ***p < 0.001 vs DMSO, IC_50_:69.25 μM), respectively. Even at high concentrations, other compounds and the positive control acarbose showed no cytotoxic effects on healthy cells.

**Table 3:** IC_50_ Values for Compounds **1–14** in HEK293 Cells

compounds	HEK293 IC_50_ (μM)
**1**	>100
**2**	>100
**3**	>100
**4**	>100
**5**	>100
**6**	>100
**7**	61.04
**8**	100
**9**	69.25
**10**	>100
**11**	>100
**12**	>100
**13**	>100
**14**	100
acarbose	>100

For patient safety, toxicity evaluation is the most important process in drug development.^17^ Thus, using the MTT test, the toxicity of all compounds on HEK-293 cells^50^ was investigated at different doses. Except for compounds **7** (IC_50_: 61.04 μM) and **9** (IC_50_: 69.25 μM), all other compounds and the positive control acarbose exhibited no cytotoxic effects on healthy cells, even at elevated concentrations. According on the antidiabetic inhibitory effects of compounds **1**–**14**, compounds **3**, **8**, and **10** have been selected as the most potential drugs. Simultaneously, it has been shown that these compounds have no negative impact on healthy cells, suggesting that the drugs may be safe.

### 3.5 Molecular Docking Results

According to molecular docking analyses, the binding energy of the α-amylase complexed with acarbose was calculated to be −6.30 kcal/mol (Table 4). All tested compounds exhibited higher binding affinities compared to the reference acarbose compound. Notably, compound **8** and **10** demonstrated the strongest inhibitory activities and strong binding affinities among the series. The binding energies of these compounds were calculated as −9.35 kcal/mol and −9.29 kcal/mol, respectively (Table 4). Examination of the 2D structure of the α-amylase complex with compound **8** revealed the formation of hydrogen bonds with amino acids Gln63, Arg195, Asp197, and Glu233; van der Waals interactions with Trp58, Tyr151, Leu162, Ser199, Val234, His299, Asp300, and Asp356; Π-Π stacked interactions with Trp59 and Tyr62; and Alkyl and Π-Alkyl interactions with His101, Leu165, Lys200, His201, Ile235, and His305 (Figure 3). Similarly, the 2D structure of the complex formed with compound 10 demonstrated hydrogen bonding with Lys200, Glu233, and Ile235; van der Waals interactions with His101, Arg161, Asp197, and Glu240; Π-Sigma interactions with Tyr151; and Alkyl and π-alkyl interactions with Tyr62 (Figure 3). The binding regions of both compounds were consistent with the interaction sites reported in the literature.^27^

**Table 4:** Molecular Docking Analysis of Compounds with α-Amylase Enzyme: IC_50_ Value, Binding Energy, and Amino Acid Interactions

compound number	IC_50_ value	binding energy α-amylase	interactions of amino acids^a^
**acarbose**	35.18 ± 0.73	–6.30	**Ile235,Thr163, Glu233, His201, Asp197,** Gly104, Thr163, Tyr62, His299
**1**	86.07 ± 0.12	–9.28	**Lys200, His201, Ile235, Glu233, Ala198, Val234,** His101, Tyr151, Leu237
**2**	53.98 ± 0.61	–9.54	**Asp197, His305, Glu233, Asp197, Asp300,** His101, Trp59, Ala198, Lys200, Ile235, His201, His305
**3**	39.73 ± 0.55	–8.95	**His101, Tyr151, Lys200, Ile235, Asp197,** Glu233, His201, Leu237, Ala307, Leu162, Ala198, Leu162
**4**	69.43 ± 0.19	–9.60	**Trp59, His305, Asp356,** Tyr62, Pro54, Trp58, His299, Trp357
**5**	60.51 ± 0.80	–9.32	**Glu233,** His305, Tyr62, His299, Trp58, Tyr62, Ala198, Lys200, Ile235, Leu165
**6**	78.05 ± 0.14	–10.38	**Trp59, Arg195, Asp197, Glu233,** Tyr62, His299, Trp58, Lys200, Ile235, His201, His305, Leu165
**7**	41.40 ± 0.26	–9.40	**Arg195, Asp197, Glu233,** Trp59, His299, Trp59, Tyr62, Lys200, Ile235, His201
**8**	30.21 ± 0.16	–9.35	**Gln63, Arg195, Asp197, Glu233,** Trp59, Tyr62, Lys200, Ile235, Leu165
**9**	51.26 ± 0.41	–9.56	**Arg195, Asp197, Thr163, Gly104,** Lys200, Ile235, Leu165, His101, His201
**10**	34.49 ± 0.37	–9.29	**Lys200, Ile235, Glu233, Leu162, Thr163, Ala198, His201, Val234,** Tyr151, Ala198, Tyr62
**11**	48.27 ± 0.39	–10.05	**Gln63,** Glu233, His201, Ala106, Leu165, Ala198, Lys200, Ile235
**12**	45.26 ± 0.10	–10.74	**Trp59, Arg195, Asp197, Glu233,** His305, Tyr62, His299, Trp58, Tyr62, Lys200, Ile235, His201, Leu165
**13**	52.75 ± 0.60	–9.65	**Lys200, Ile235, Leu237, Glu240, Glu233, Ala198, His201, Val234,** Tyr151, Tyr62, Ala307
**14**	43.09 ± 0.72	–9.59	**Arg195, Asp197, Glu233,** Trp59, Tyr62, Lys200, Ile235, Leu165, His201

a The amino acids highlighted in bold signify hydrogen bond formation.

**Figure 3:**
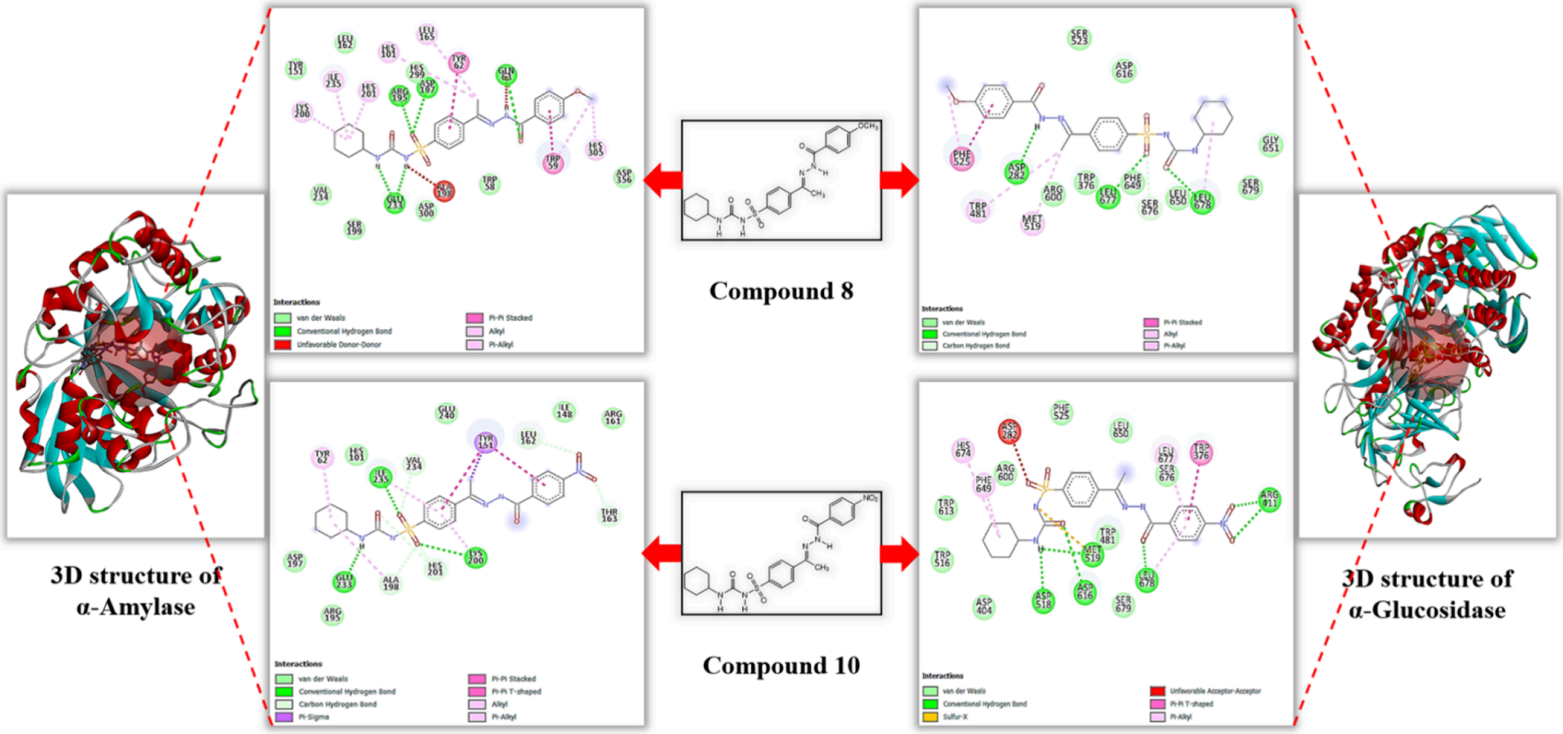
2D analysis of the lowest-energy binding conformations of compounds **8** and **10**, which exhibit the best binding affinities and biological activity for α-amylase and α-glucosidase enzymes.

For the α-glucosidase enzyme, the binding energy of the complex with acarbose was calculated as −4.66 kcal/mol (Table 5)**.** Notably, all tested compounds demonstrated higher binding affinities compared to acarbose. Among them, compounds **8** and **10** exhibited the strongest inhibitory activities, with binding energies of −8.57 kcal/mol and −9.10 kcal/mol, respectively (Table 5)**.** The analysis of the 2D structures of the α-glucosidase complexes with compounds **8** and **10** revealed distinct interaction profiles (Figure 3)**.** For compound **8,** hydrogen bonds were observed with Asp282, Leu677, and Leu678, while van der Waals interactions involved Ser523, Arg600, Asp616, Phe649, Leu650, Gly651, and Ser679. Additionally, π-π stacking occurred with Phe525, and Alkyl and Π-Alkyl interactions were identified with Trp481 and Met519. In the case of compound **10,** hydrogen bonding was noted with Arg411, Asp518, Met519, Asp616, and Leu678, and van der Waals interactions included Asp404, Trp481, Trp516, Phe525, Arg600, Trp613, Leu650, Ser676, and Ser679. Furthermore, Π-Π stacking interactions were observed with Trp376, and Π-Alkyl interactions occurred with Phe649, His674, and Leu677. These interaction regions for both compounds align with the binding sites previously reported in the literature.^51^

**Table 5:** Molecular Docking Analysis of Compounds with α-Glucosidase Enzyme: IC_50_ Value, Binding Energy, and Amino Acid Interactions

compound number	IC_50_ value	binding energy α-glucosidase	interactions of amino acids^a^
**acarbose**	80.78 ± 0.25	–4.66	**Leu677, Leu678, Asp404**, **Asp282**
**1**	108.05 ± 0.69	–8.79	**Ser523, Leu677, Leu678, Asp282, Ser676,** Phe525, Met519
**2**	68.75 ± 0.37	–9.19	**Ser523, Leu677, Leu678, Asp282, Ser676,** Phe525, Met519, Trp481
**3**	45.34 ± 0.36	–8.62	**Leu677, Leu678, Asp282, Ser676,** Phe525, Met519, Trp481
**4**	84.97 ± 0.46	–9.60	**Leu678, Asp282,** Trp376, Leu677, Trp481, Phe649,
**5**	77.36 ± 0.31	–9.18	**Ser523, Leu677, Leu678, Asp282, Ser676,** Phe525, Trp481, Met519
**6**	96.68 ± 0.28	–9.03	**Leu677, Leu678, Asp282, Ser676,** Phe525, Trp481, Met519
**7**	46.14 ± 0.33	–9.05	**Ser523, Leu677, Leu678, Asp282, Ser676,** Phe525, Met519
**8**	38.06 ± 0.80	–8.57	**Leu677, Leu678, Asp282, Ser676,** Phe525, Met519, Trp481, Phe525
**9**	61.18 ± 0.69	–8.68	**Asp282, Ser523, Phe525, Ser676,** Trp481, Leu650, Leu678, Ala555
**10**	40.44 ± 0.23	–9.10	**Arg411, Asp616, Leu678, Asp518, Met519,** Trp376, Phe649, His674, Leu677
**11**	57.06 ± 0.78	–9.04	**Leu677, Leu678, Ser676,** Asp282, Met519, Trp481, Ala555
**12**	50.39 ± 0.11	–9.71	**Ser523, Leu677, Leu678, Asp282, Ser676,** Phe525, Trp481, Met519
**13**	66.38 ± 0.37	–8.32	**Arg411, Asp616,** Phe649, His674, Leu677
**14**	52.25 ± 0.44	–9.02	**Ser523, Leu677, Leu678, Asp282, Ser676,** Phe525, Trp481, Met519

a The amino acids highlighted in bold signify hydrogen bond formation.

### 3.6 Molecular Dynamics (MD) Simulations and MM/PBSA Free Energy Analyses Results

RMSD (Root Mean Square Deviation) analysis was performed to evaluate the stability and structural changes of the enzyme-ligand complexes. For the α-amylase-**8** complex, the RMSD values ranged between 0.20 and 0.47 nm for the entire complex, 0.0005 and 0.36 nm for the enzyme, and 0.0004 and 0.38 nm for the compound. Similarly, in the α-amylase-**10** complex, the RMSD values were 0.24–0.48 nm for the complex, 0.0005–0.36 nm for the enzyme, and 0.0004–0.40 nm for the compound. In the simulations involving the α-glucosidase enzyme, the RMSD values for the α-glucosidase-**8** complex ranged from 0.72 to 1.08 nm for the complex, 0.0005–0.38 nm for the enzyme, and 0.0004–0.38 nm for the compound. For the α-glucosidase-**10** complex, the RMSD values were 0.75–1.00 nm for the complex, 0.0004–0.45 nm for the enzyme, and 0.0004–0.48 nm for the compound (Figure 4)**.**

**Figure 4:**
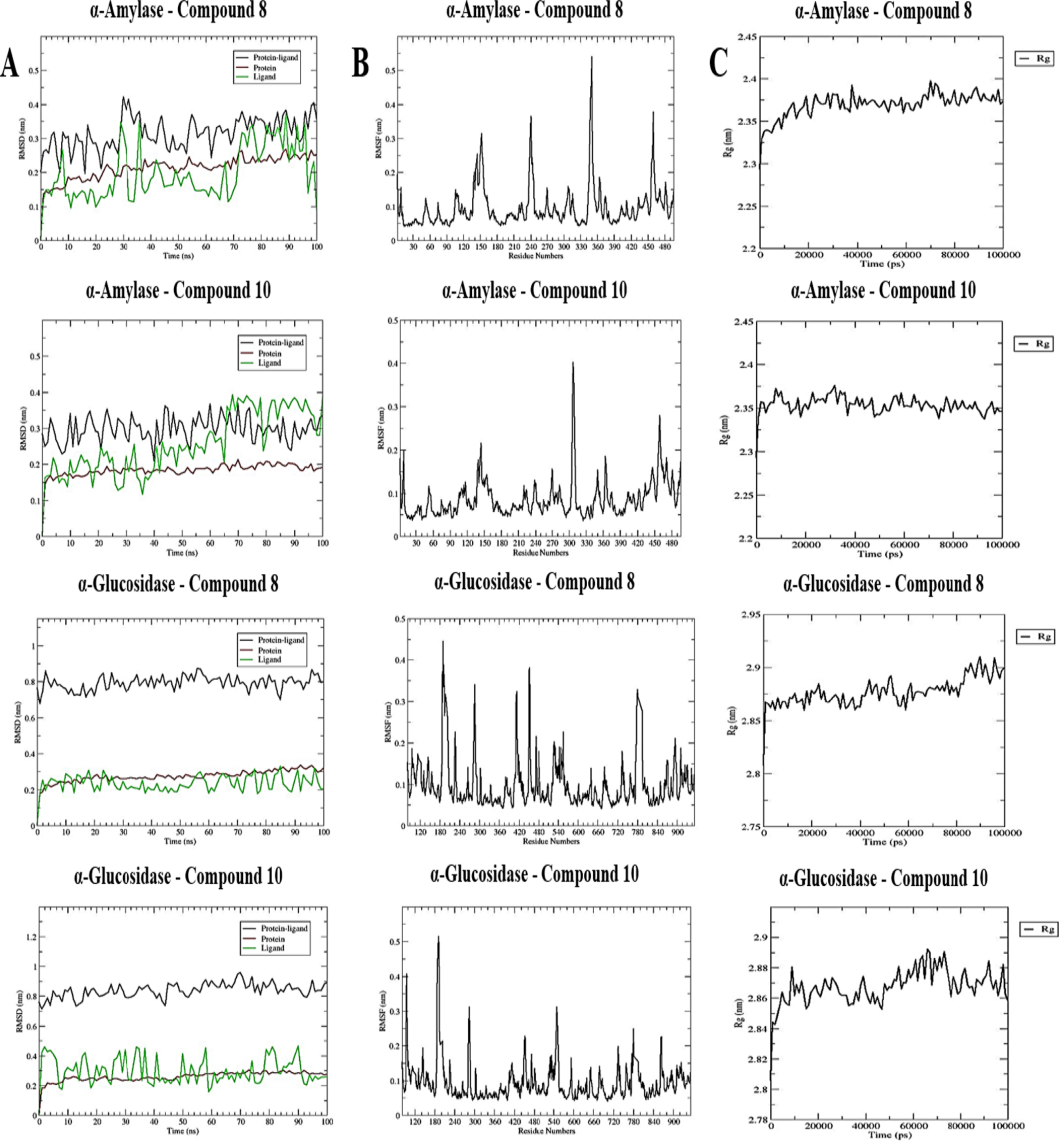
(A) The RMSD changes of the protein, ligand, and complex structures over 250 ns. The protein is represented in red, the complex (protein–ligand) in black, and the ligand in green. (B) The RMSF profile of the target enzymes in the complex structures. (C) The radius of gyration (Rg) analysis of the target enzyme structures during the 250 ns MD simulation.

RMSF (Root Mean Square Fluctuation) analysis, which provides a detailed evaluation of the dynamic properties of the complexes, revealed RMS fluctuations for amino acids in the α-amylase-**8** complex between 0.04 and 0.89 nm, and in the α-amylase-10 complex between 0.04 and 0.84 nm. In both complexes, low RMSF values were observed for key functional amino acids, including Ile148, Tyr151, Leu162, Thr163, Arg195, Ala198, Lys200, Val234, Ile235, and His305. Similarly, in the α-glucosidase-131 complex, the RMS fluctuations for all amino acids ranged between 0.03 and 0.63 nm, and in the α-glucosidase-10 complex, they ranged from 0.03 to 0.85 nm. Low fluctuations were also observed in amino acids critical for the enzyme’s functional activities, such as Asp616, Leu650, Gly651, Leu678, and Ser679 (Figure 4)**.**

The Rg (Radius of Gyration) analysis, performed to assess the structural compactness of the complexes during the simulation, indicated that the complexes remained stable in a compact structure throughout. For the α-amylase-**8** and α-amylase-**10** complexes, the enzyme’s Rg values were calculated to be between 1.52 and 2.43 nm and 1.50–2.42 nm, respectively, over 250 ns. Similarly, in the α-glucosidase-**8** and α-glucosidase-**10** complexes, the enzymes maintained compact stability, with Rg values ranging from 1.99 to 2.92 nm and 2.00–2.94 nm, respectively (Figure 4)**.**

MM/PBSA binding free energy analyses thoroughly assessed the complexes’ binding energies and energy components. The binding free energy of the α-amylase-**8** complex was calculated as −72.277 ± 22.647 kJ/mol. When examining the energy components, the van der Waals energy was found to be −129.989 ± 40.595 kJ/mol, electrostatic energy −26.466 ± 19.528 kJ/mol, polar solvation energy 100.318 ± 54.989 kJ/mol, and solvent accessible surface area (SASA) energy −16.141 ± 4.198 kJ/mol. For the α-amylase-**10** complex, the binding free energy was determined to be −80.187 ± 16.730 kJ/mol, with contributions from van der Waals energy −180.644 ± 20.148 kJ/mol, electrostatic energy −49.219 ± 13.410 kJ/mol, polar solvation energy 169.128 ± 24.820 kJ/mol, and SASA energy −19.451 ± 1.303 kJ/mol.

Similarly, for the α-glucosidase-**8** complex, the binding free energy was found to be −76.573 ± 26.145 kJ/mol. The energy components included van der Waals energy −116.993 ± 19.850 kJ/mol, electrostatic energy −32.332 ± 12.157 kJ/mol, polar solvation energy 87.223 ± 29.554 kJ/mol, and SASA energy −14.472 ± 2.211 kJ/mol. For the α-glucosidase-**10** complex, the binding free energy was calculated as −52.621 ± 39.555 kJ/mol, with van der Waals energy −99.101 ± 29.446 kJ/mol, electrostatic energy −18.002 ± 19.776 kJ/mol, polar solvation energy 76.220 ± 51.165 kJ/mol, and SASA energy −11.738 ± 3.348 kJ/mol.

### 3.7 In Silico ADME and Toxicity Analysis

For a potent molecule to be effective as a drug, it must reach its target in the body in sufficient concentration and remain there in a bioactive form long enough for the expected biological events to occur. ADME (which stands for Absorption, Distribution, Metabolism, and Elimination) is an important concept in the context of cellular biology and biochemistry that describes the potential effect of a chemical or drug on a living system. This is because the movement and metabolism of molecules are determined by the physicochemical properties of the molecule and the host system. The movement of molecules is called “kinetics” or “pharmacokinetics” and chemical properties such as polarity, molecular weight, molecular size, chirality, HOMO/LUMO, and more all have an impact on the ADME potential of a molecule. However, the ADME concept can also be applied to non-pharmaceutical compounds, including those resulting from toxic exposure. SwissADME data is given in Table 6.

**Table 6:** SwissADME Prediction of Compounds **1**–**14** through Online Software^a^

no	MW	TPSA (A^0^)	Lipinski’s violation	mi log *P*	GI absorption	*n*-ON	*n*-OHNH	BBB permeability
**1**	442.53	125.11	yes	2.76	low	5	3	no
**2**	476.98	108.04	yes	3.64	low	4	3	no
**3**	460.52	125.11	yes	2.52	low	6	3	no
**4**	566.45	108.04	yes	3.96	low	4	3	no
**5**	521.43	125.11	yes	2.57	low	5	3	no
**6**	526.65	153.86	yes	3.22	low	4	3	no
**7**	454.59	108.04	yes	3.57	low	4	3	no
**8**	472.56	134.34	yes	2.60	low	6	3	no
**9**	487.53	170.93	yes	2.64	low	7	3	no
**10**	487.53	153.86	yes	2.49	low	6	3	no
**11**	492.59	125.11	yes	4.05	low	5	3	no
**12**	492.59	125.11	yes	4.05	low	5	3	no
**13**	487.53	170.93	yes	2.64	low	7	3	no
**14**	476.98	125.11	yes	2.36	low	5	3	no

a Molecular weight (MW), Topological polar surface area (TPSA), Logarithm of partition coefficient between *n*-octanol and water (mi log *P*), Number of hydrogen bond donors (*n*-OHNH). Number of hydrogen bond acceptors (*n*-ON).

Computer-aided drug design is gaining importance in drug development in order to increase the number of designed compounds, increase the success rate, shorten the research and development process and keep it at a minimum level economically. In this context, many computer software containing different methods support practical studies. The radar images taken using the Swiss-ADME program, one of them, are evaluated, many parameters such as LIPO-lipophilicity, SIZE-molecular weight, POLAR-polarity, INSOLU-solubility, INSATU-saturation, FLEX-flexibility can be examined.^52–56^ The radar images of the synthesized compounds (**1**–**14**) according to Swiss-ADME program were taken and evaluated in Figure 5, suitable radar images of compounds **1**, **2**, **8**, **11**, **12**, and **14** were determined. It is observed that compounds **7**, **9**, **10**, and **13** are more polar than they should be, and the lipophilicity of compounds **3**, **4**, **5**, and **6** is higher than it should be.

**Figure 5:**
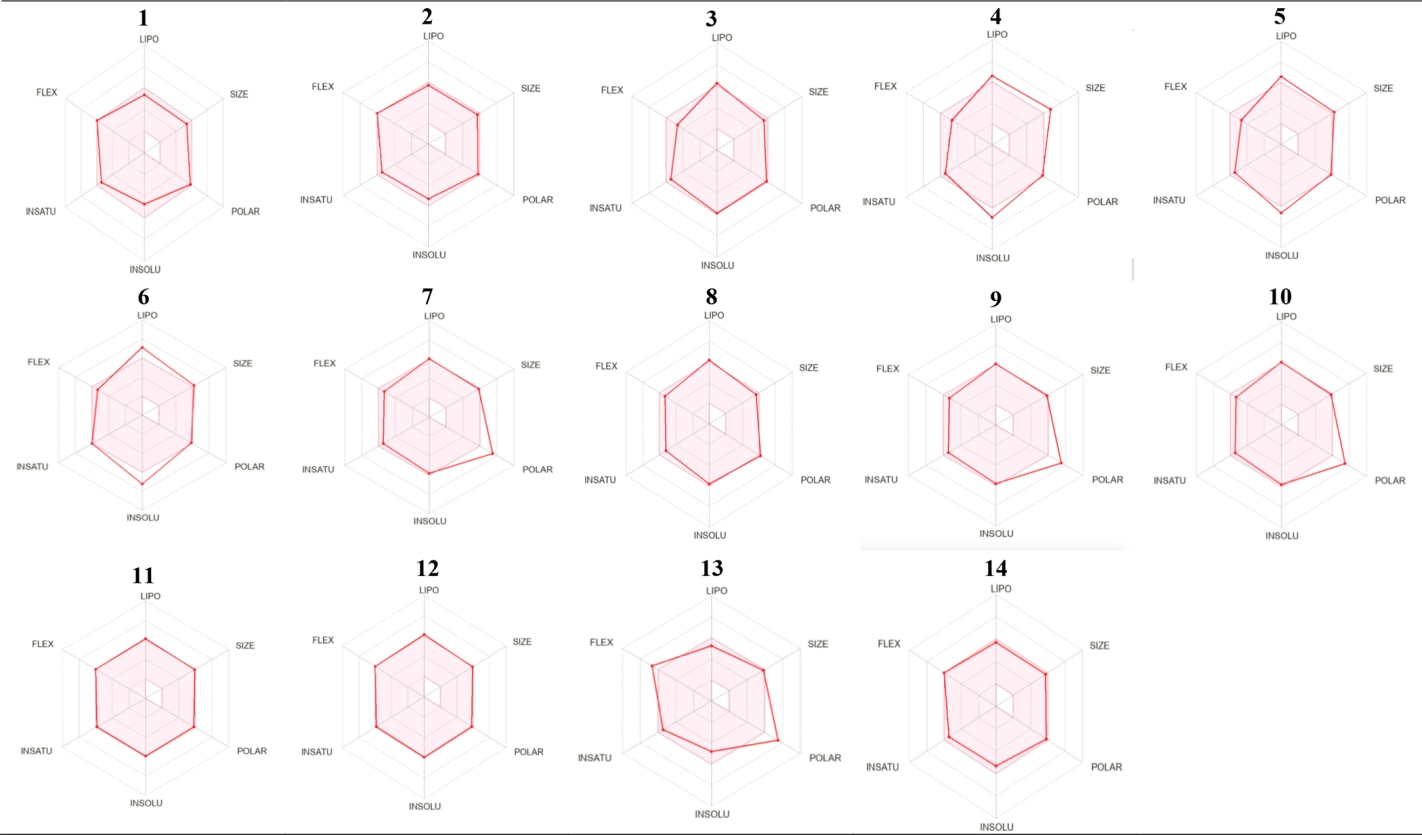
Bioavailability radar visual of compounds **1**–**14**.

Toxicokinetics studies describe the rates at which a substance enters a biological system and what happens to the substance once it enters the system. Measuring toxicity is an important step in drug development. However, current experimental methods used to predict drug toxicity are expensive and time-consuming. This indicates that they are not suitable for large-scale evaluation of drug toxicity at the early stage of drug development. For this reason, using the OSIRIS program, which is a computational model that can predict drug toxicity risks, four toxicity risks are included in Table S1 mutagenic effect, tumorogenic effect, irritant effect and reproductive effect. Except compound **11** from synthesized hydrazone compounds, it was observed that all hydrazones did not have any effect when looking at four toxicity risks: mutagenic effect, tumorogenic effect, irritant effect and reproductive effect. In the compound **11** was seen that it had a tumorogenic effect.

The toxicities and toxicity classes of the target molecules were analyzed by an in silico study using the Protox-II web server and are given with the data in Figure 6. Toxic doses are usually given as LD_50_ values in mg/kg body weight. LD_50_ is the median lethal dose, that is, the dose at which 50% of subjects die when exposed to a compound. Toxicity classes are defined according to the globally harmonized classification system (GHS) of labeling of chemicals. LD_50_ values are given in [mg/kg]: Class I: fatal if swallowed (LD_50_ ≤ 5), Class II: fatal if swallowed (5 < LD_50_ ≤ 50), Class III: toxic if swallowed (50 < LD_50_ ≤ 300), Class IV: harmful if swallowed (300 < LD_50_ ≤ 2000), Class V: may be harmful if swallowed (2000 < LD_50_ ≤ 5000), Class VI: nontoxic (LD_50_ > 5000). While compounds **1**, **2**, **7**, **11**, **12**, and **14** belong to LD_50_ value of 2000 mg/kg; compound **5** is LD_50_ value of 3000 mg/kg. Compound **8**, which is LD_50_ value of 3250 mg/kg. Compounds **3**, **4**, and **6** is LD_50_ value of 4000 mg/kg. Compounds **9**, **10**, and **13** was found as LD_50_ value of 15000 mg/kg. The toxicity class of compounds provides a measure of total toxicity. Class I is the most toxic and most dangerous class of toxicity, and class VI is the least toxic. Compound **1**, **2**, **7**, **11**, **12**, and **14** toxicity class IV; compounds; **3**, **4**, **5**, **6**, **8**, and **10** toxicity class V; compounds **9**, **10**, and **13** were determined as toxicity class VI.

**Figure 6:**
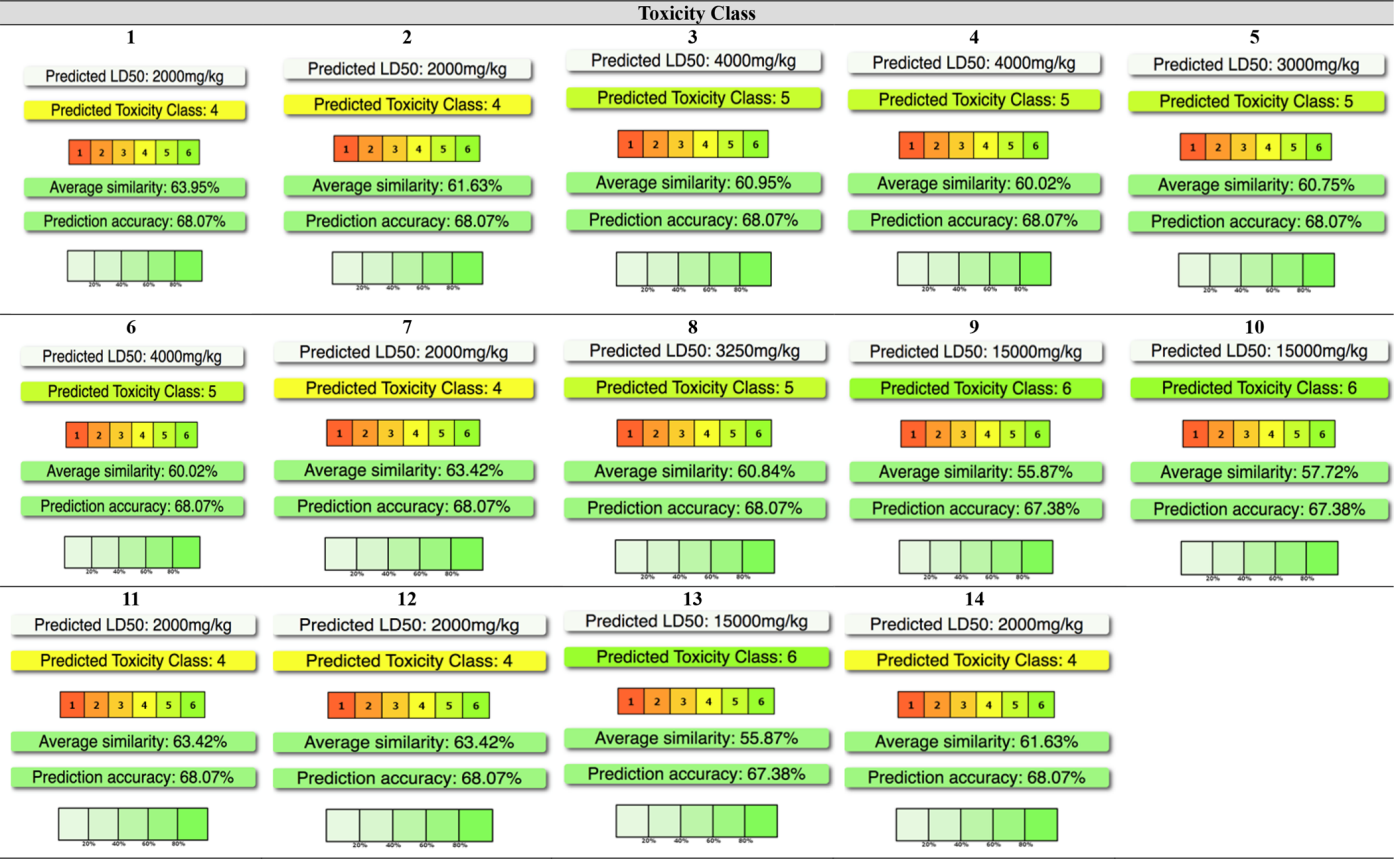
Protox-II*** data of synthesized compounds. ***­(https://tox-new.charite.de/protox_II/).

PASS Online, a software developed by the Russian Institute of Biomedical Chemistry (IBMC) and freely accessible by browsers, predicts the biological activities of compounds based on the structural formulas of synthesized drug-like synthetic organic compounds and provides data. These predictions are based on drug substances, new drug candidates in various stages of clinical and preclinical research, and analysis of structure–activity relationships. Prediction results in the PASS Online program are realized as “active” or “inactive”. In the Table 7, Prediction results in the PASS Online program of diabetic activity of the synthesized hydrazone compounds was given. Pa = probability of being active, Pi = probability of being inactive and maximum Δ*P* value = Pa-Pi was calculated to estimate the most probable activities. Under these findings, the antidiabetic activity values of the synthesized hydrazone compounds were calculated. According to Δ*P* values, while compounds **2**, **3**, **1**, **7**, and **13** are the most active; compounds **4**, **9**, **10**, and **14** were observed showing the least activity. As a result, it is thought that especially compound **8** could be a leading compound among the synthesized hydrazone derivatives.

**Table 7:** PASS^a^ Online Data of the Antidiabetic Activity of the Synthesized Compounds

**compound**	**Pa**	**Pi**	**ΔP**
**1**	0.371	0.052	0.319
**2**	0.374	0.051	0.323
**3**	0.372	0.052	0.320
**4**	0.288	0.089	0.199
**5**	0.359	0.056	0.303
**6**	0.360	0.056	0.304
**7**	0.368	0.053	0.315
**8**	0.349	0.060	0.289
**9**	0.251	0.113	0.138
**10**	0.254	0.112	0.142
**11**	0.345	0.062	0.283
**12**	0.345	0.062	0.283
**13**	0.374	0.051	0.323
**14**	0.251	0.113	0.138

a (http://way2drug.com/ddi/).

## 4 Conclusions

In order to provide a more effective therapeutic effect than Acetohexamide used for diabetes, new Acetohexamide-based hydrazone derivatives were designed and synthesized based on the broad bioactivity, target selectivity and bioavailability of the nitrogen atoms in their chemical structure. According to the OSIRIS and Protox_II programs, it is estimated that the probability of any toxicity of Acetohexamides-derived hydrazone is low. It is seen that compounds **1**, **2**, **3**, **8**, **11**, **12**, and **14** may have significant advantages due to their bioavailability. In vitro, compounds **3**, **8**, and 10 inhibit α-amylase; Other synthesized compounds of compounds **1, 4**, and **6,** except, were all found to inhibit α-glucosidase. Enzyme kinetic studies showed that compounds **8** is uncompetitive inhibitors against both α-amilase and α-glucosidase. In this context, compounds **3**, **8**, and **10** are promising to be used to inhibit the both enzymes. According to our findings, compounds **3**, **8**, and **10** showed the possibility of inhibiting the enzymes α-amylase and α-glucosidase while not causing any damage to healthy cells. However, compounds **1**–**14** did not affect PPAR gamma activation. In silico molecular docking analysis revealed that the binding interactions with α-amylase and α-glucosidase were consistent with the experimental data. Furthermore, molecular simulation analyses demonstrated that these interactions remained stable over 100 ns. These findings offer valuable insights into the structural and energetic properties of active compounds binding to α-amylase and α-glucosidase, supporting the rational design of targeted therapies for diabetes treatment.

## Supplementary Material



## Notes

1H NMR, ^13^C NMR, FT-IR, and mass spectrum of compounds **1**–**14** (PDF)
