# Supplementary material for: Design, Synthesis, Antidiabetic Activity and In Silico Studies of New Hydrazone Derivatives Derived from Acetohexamide
Source: ACS Omega. 2025 Sep 19;10(38):43916–31. doi: 10.1021/acsomega.5c04642 (PMC12489694; doi:10.1021/acsomega.5c04642)
Supplement: Supplementary file 1 [file ao5c04642_si_001.pdf]

SUPPLEMENTARY DATA

Title: Design, Synthesis, Antidiabetic Activity and In silico Studies of New Hydrazone Derivatives Derived from Acetohexamide

Bedriye Seda Kurşun Aktar[a,*], Yusuf Sıcak[b], Emine Elçin Oruç-Emre[c], Rabia Kılıç[c], Ebru Sağlam[d], Demet Taşdemir[d,e], Süleyman Kaya[f], Gizem Tatar Yılmaz[f,g,h], Ayse Sahin Yaglioglu[i]

[a]Department of Hair Care and Beauty Services, Yeşilyurt Vocational School, Malatya Turgut Özal University, Malatya, Türkiye

[b]Department of Medicinal and Aromatic Plants, Köyceğiz Vocational School, Mugla Sitki Kocman University, Köyceğiz, Muğla, Türkiye

[c]Department of Chemistry, Faculty of Arts and Sciences, Gaziantep University, Gaziantep, Türkiye

[d]Respiratory Diseases and Respiratory Surgery Research and Practice Center, Gaziantep University, Gaziantep, Türkiye

[e]Department of Medical Biochemistry, Faculty of Medicine, Gaziantep University, Gaziantep, Türkiye

[f]Department of Biostatistics and Medical Informatics, Faculty of Medicine, Karadeniz Technical University, Trabzon, Türkiye

[g]Karadeniz Technical University, Institute of Health Sciences, Department of Bioinformatics, 61080, Trabzon, Türkiye

[h]Yılmaz Bilişim R&D Consulting Software Engineering and Services Trade Limited Company 61081, Trabzon, Türkiye

[i]Department of Chemistry and Chemical Process Technology, Technical Sciences Vocational School, Amasya University, Amasya, Türkiye

***Correspondence Author:**

Bedriye Seda Kurşun Aktar

Department of Hair Care and Beauty Services, Yeşilyurt Vocational School, Malatya Turgut Özal University, Malatya, Türkiye

Phone number: +90 252 211 32 58

Fax number: +90 252 211 50 41

E-mail: bseda.kursunaktar@ozal.edu.tr

Table S1. OSIRIS** data of synthesized compounds.

**Compounds**	**Toxicity Effect**			
	**Mutagenic**	**Tumorogenic**	**Irritant**	**Reproductive**
**1**	-	-	-	-
**2**	-	-	-	-
**3**	-	-	-	-
**4**	-	-	-	-
**5**	-	-	-	-
**6**	-	-	-	-
**7**	-	-	-	-
**8**	-	-	-	-
**9**	-	-	-	-
**10**	-	-	-	-
**11**	-	-	-	-
**12**	-	-	-	-
**13**	-	-	-	-
**14**	-	-	-	-

**(https://www.organic-chemistry.org/prog/peo/) -: No effect

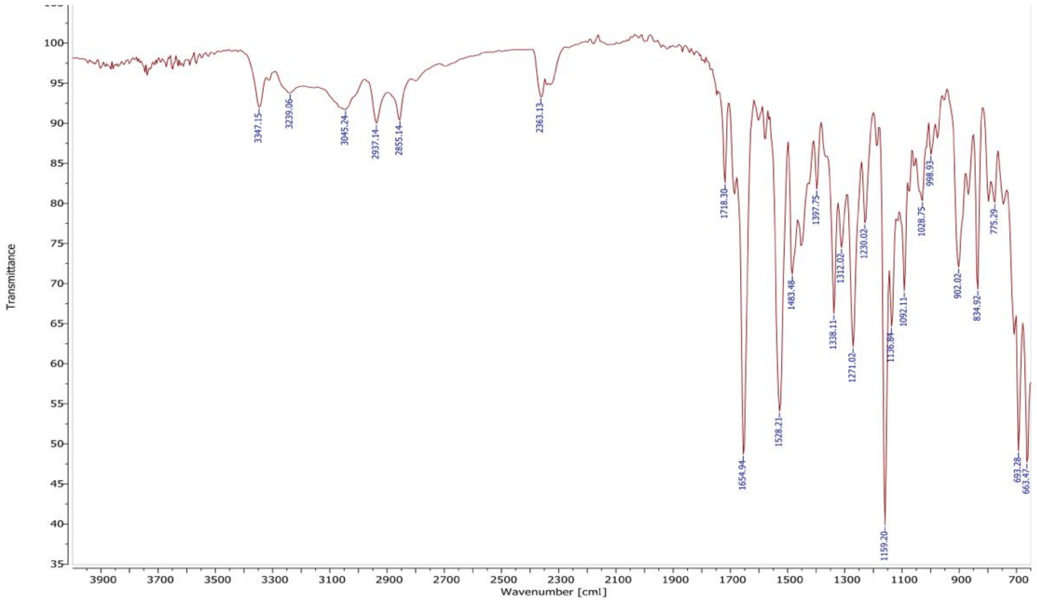

Figure S1. FTIR spectrum of compound **1**

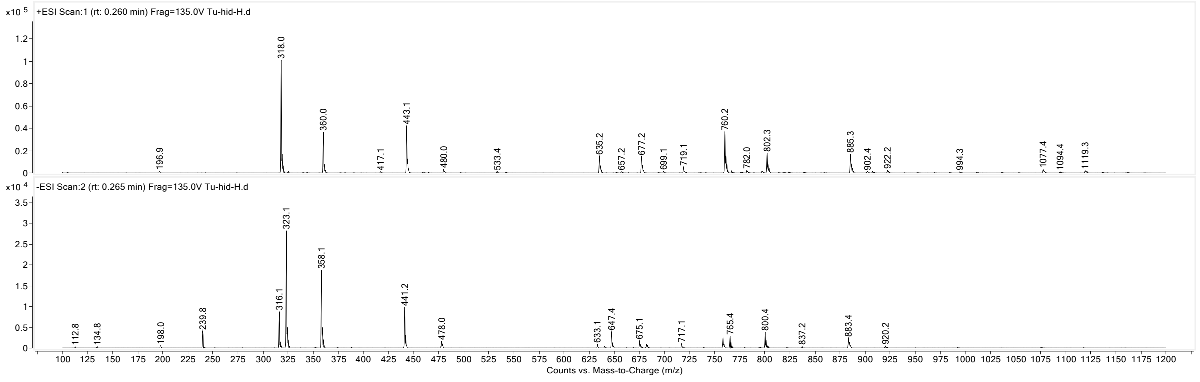

Figure S2. Mass spectrum of compound **1**

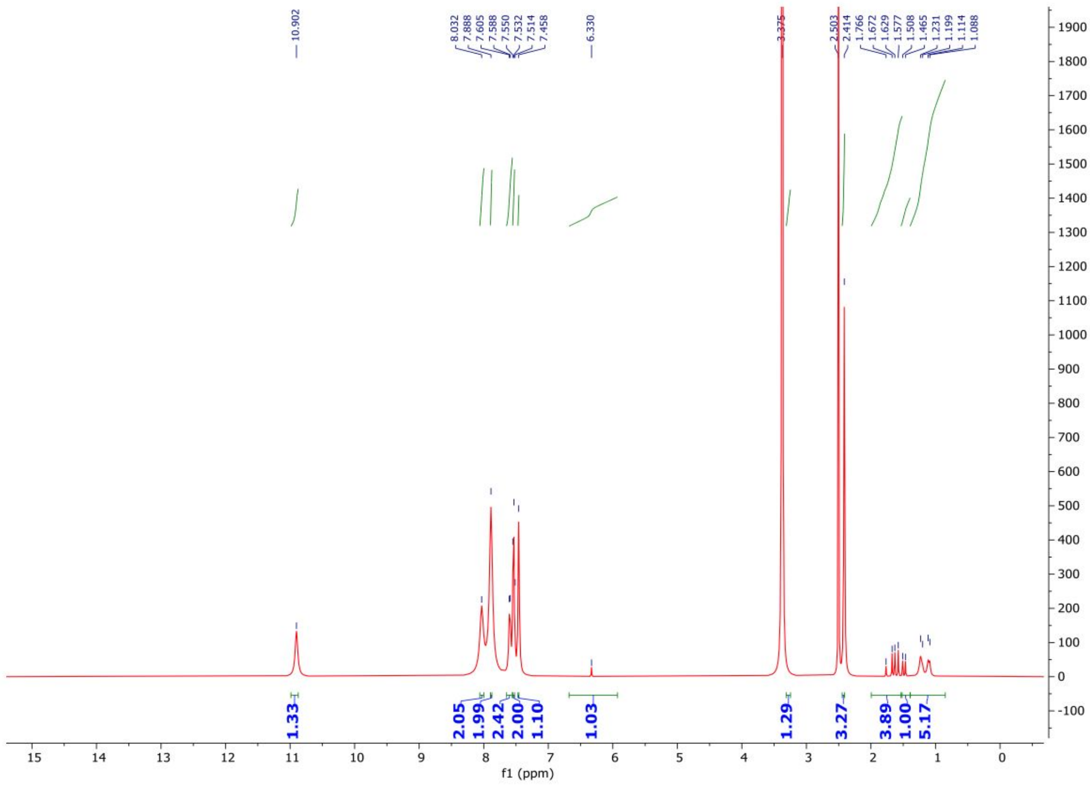

Figure S3. ^{1}H NMR spectrum of compound **1**

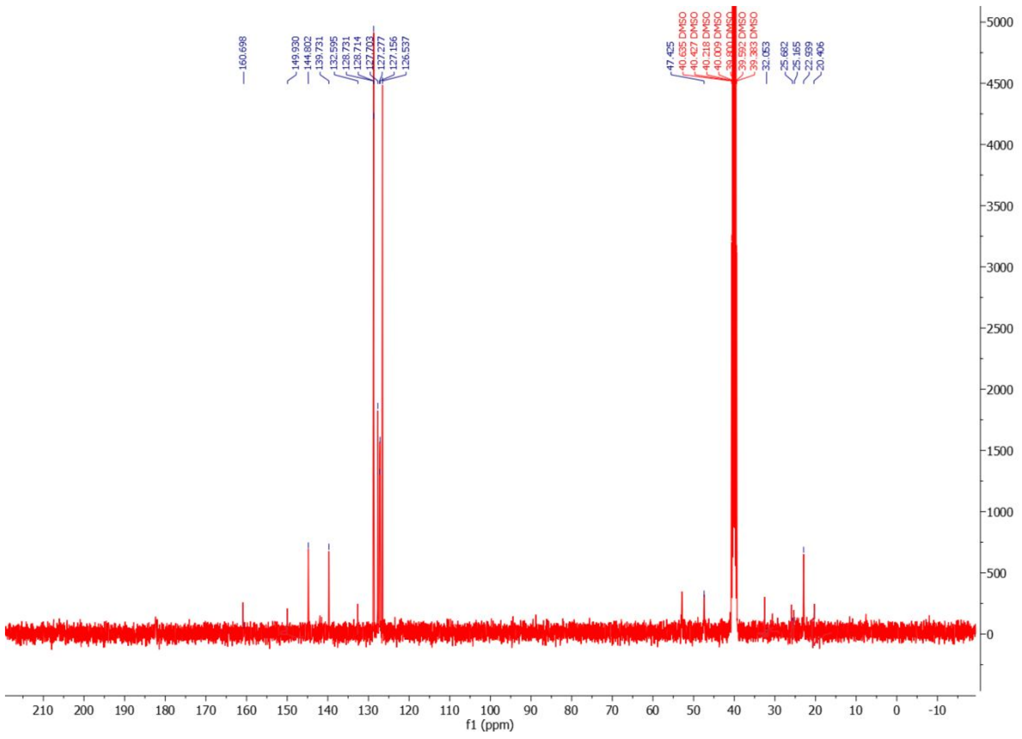

Figure S4 ^{13}C NMR spectrum of compound **1**

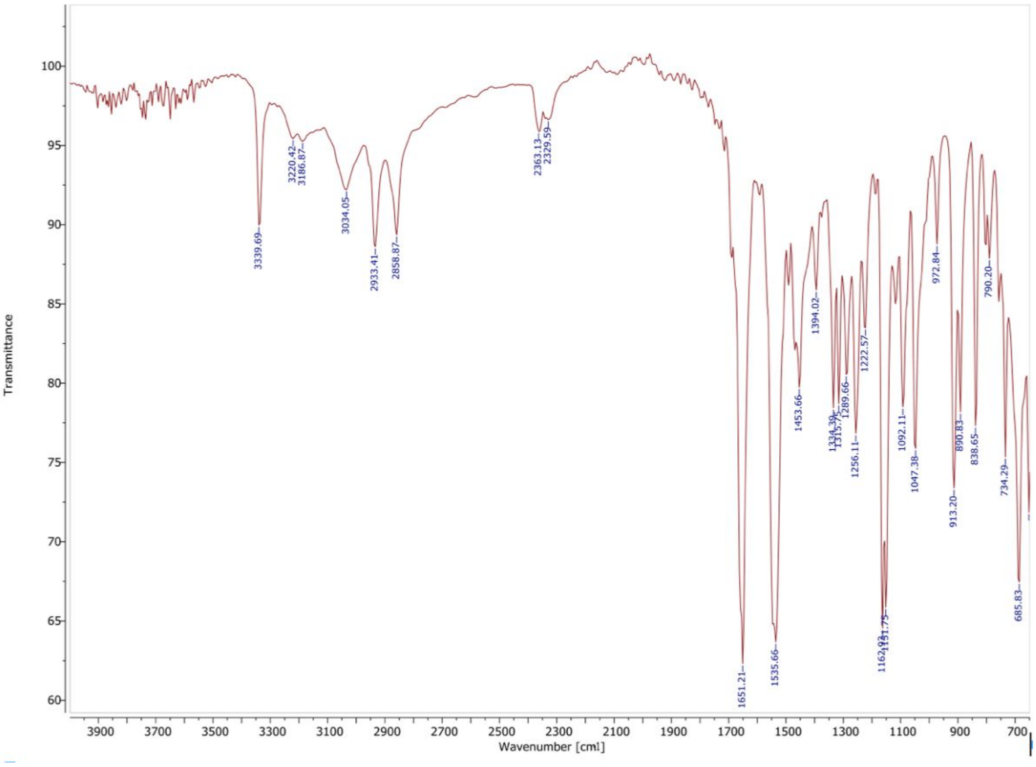

Figure S5. FTIR spectrum of compound **2**

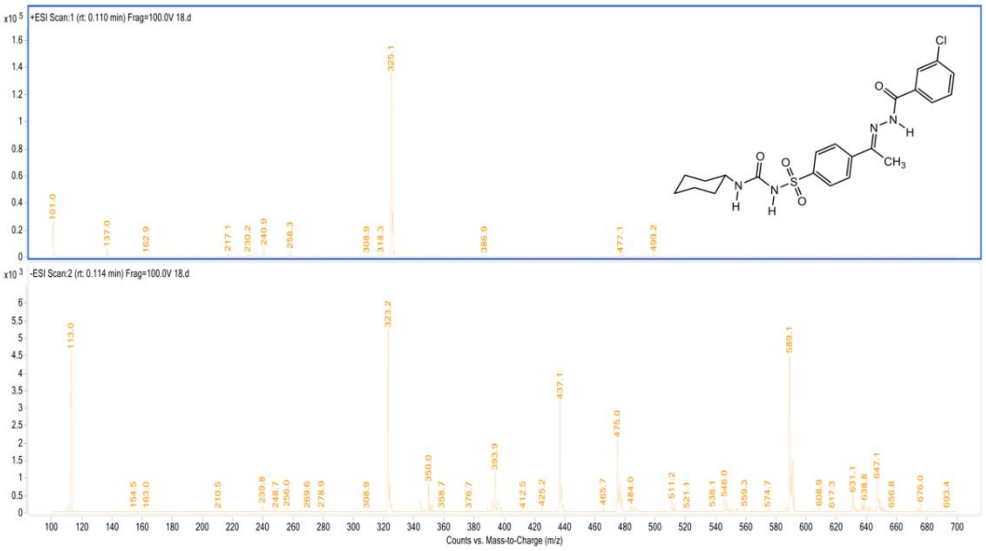

Figure S6. Mass spectrum of compound **2**

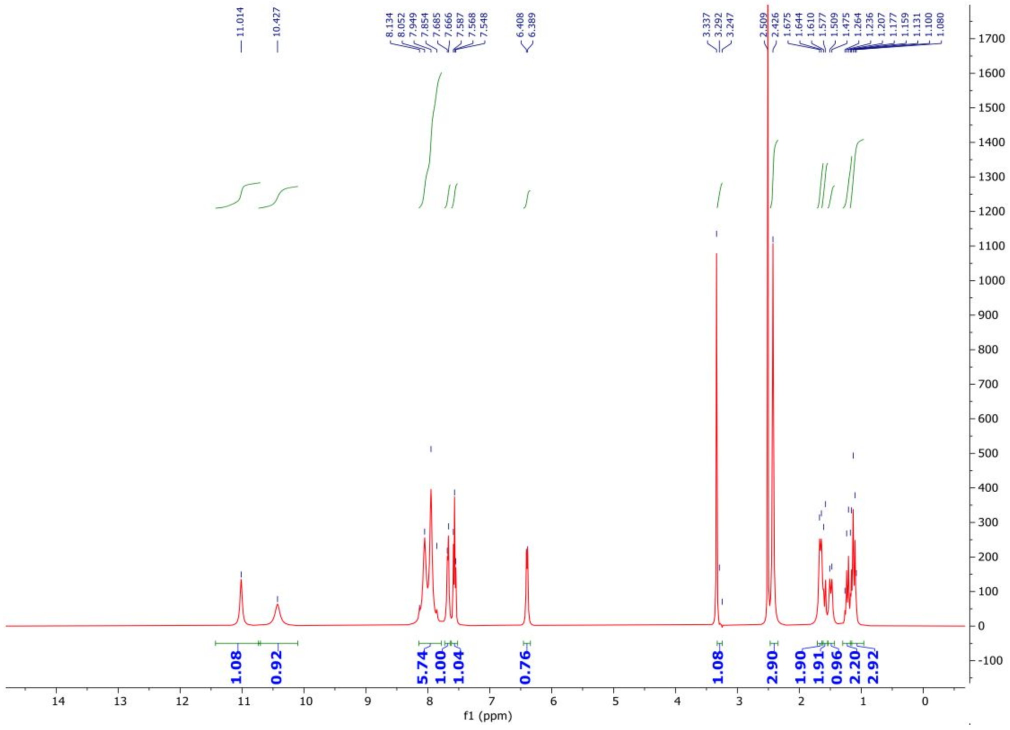

Figure S7. ^{1}H NMR spectrum of compound **2**

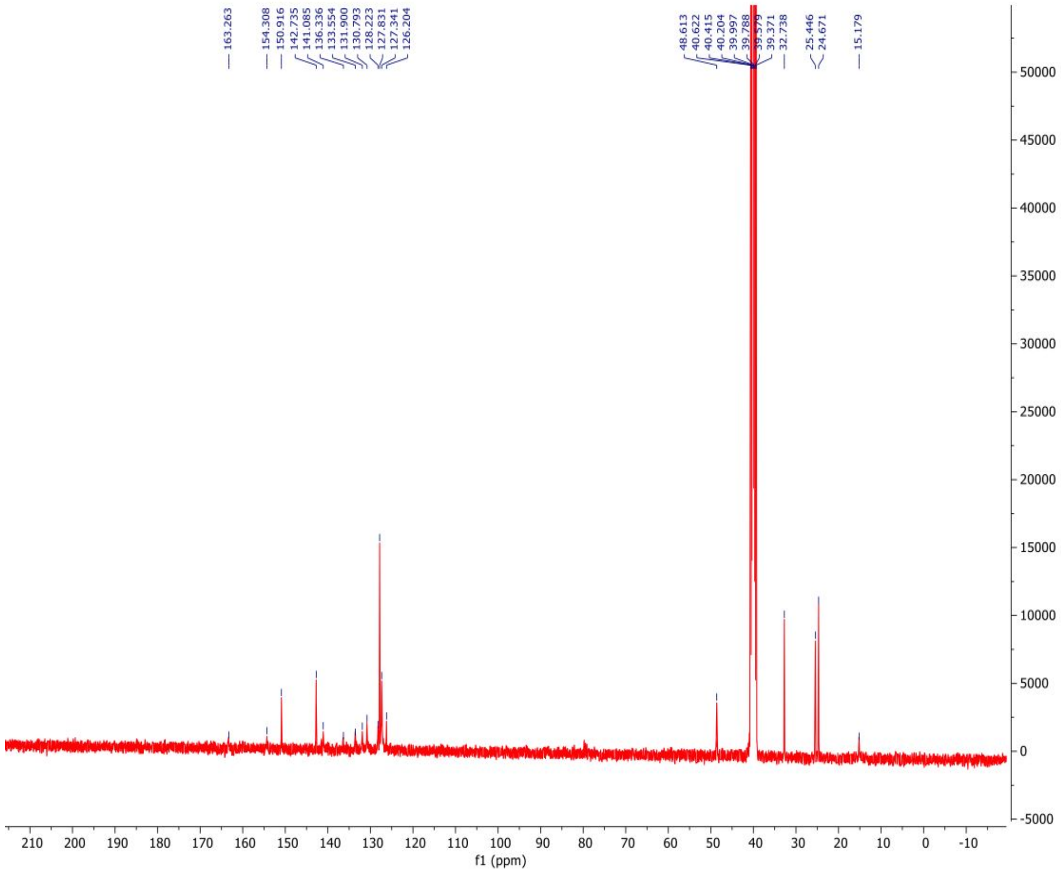

Figure S8 ^{13}C NMR spectrum of compound **2**

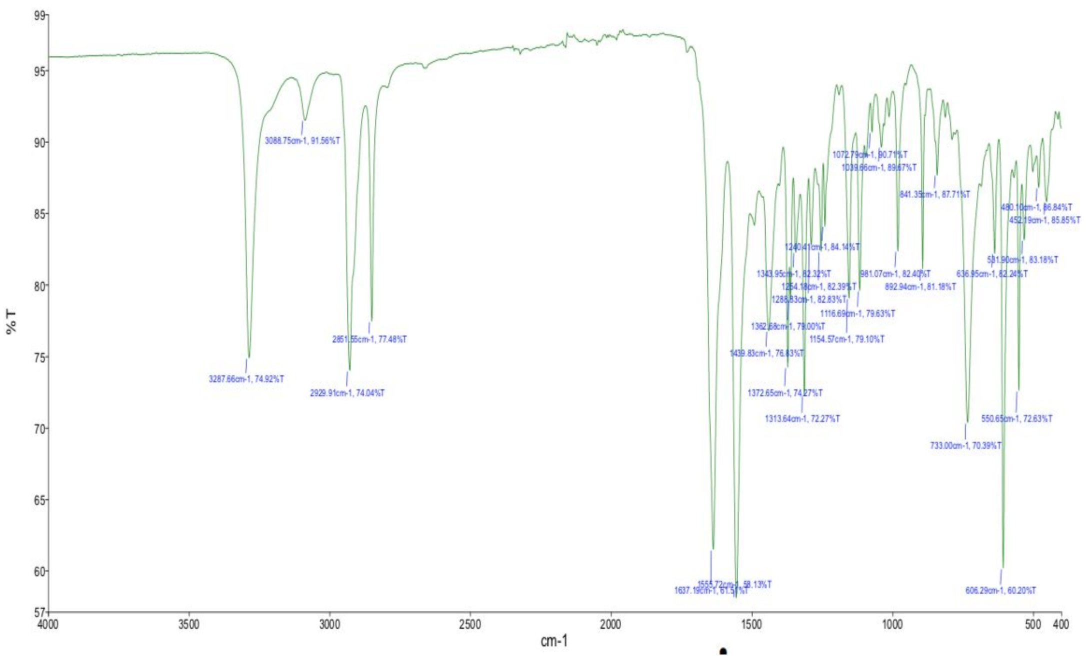

Figure S9. FTIR spectrum of compound **3**

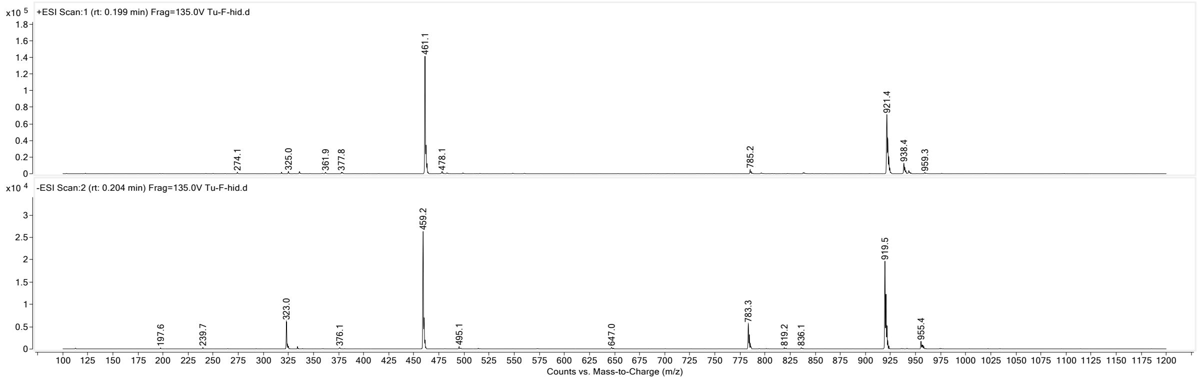

Figure S10. Mass spectrum of compound **3**

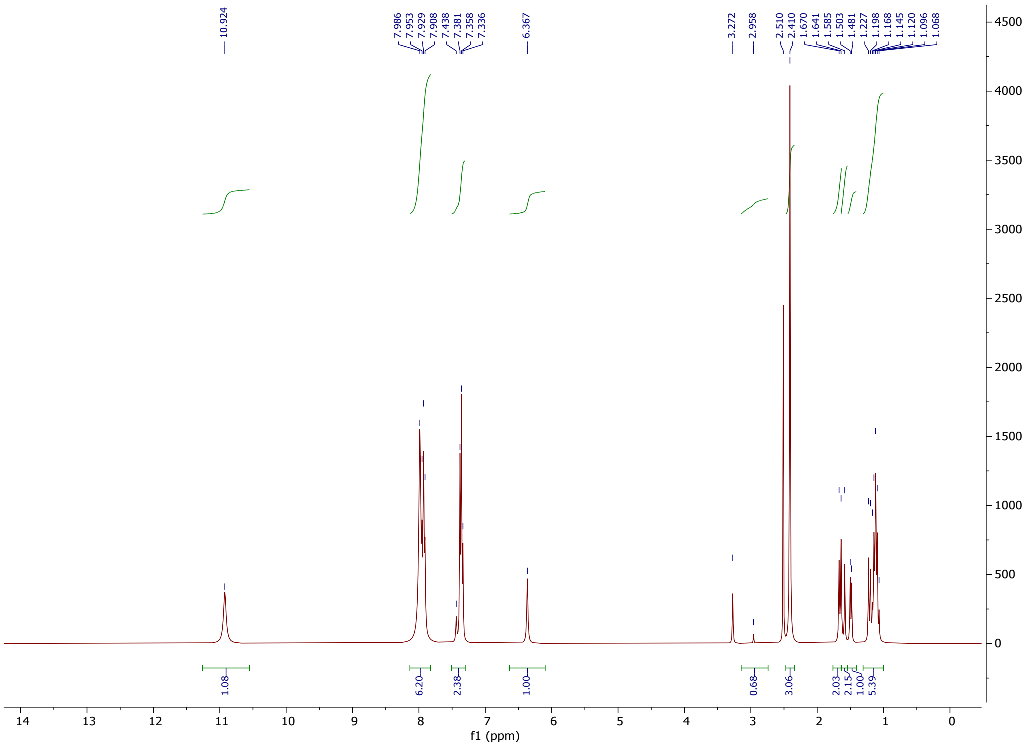

Figure S11. ^{1}H NMR spectrum of compound **3**

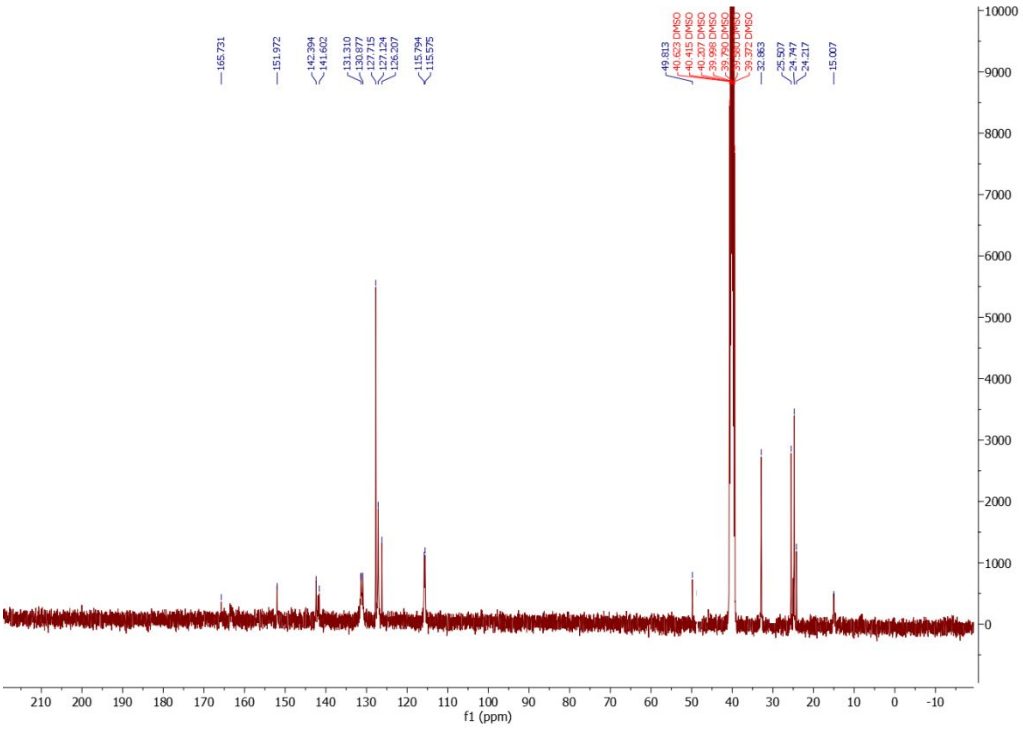

Figure S12. ^{13}C NMR spectrum of compound **3**

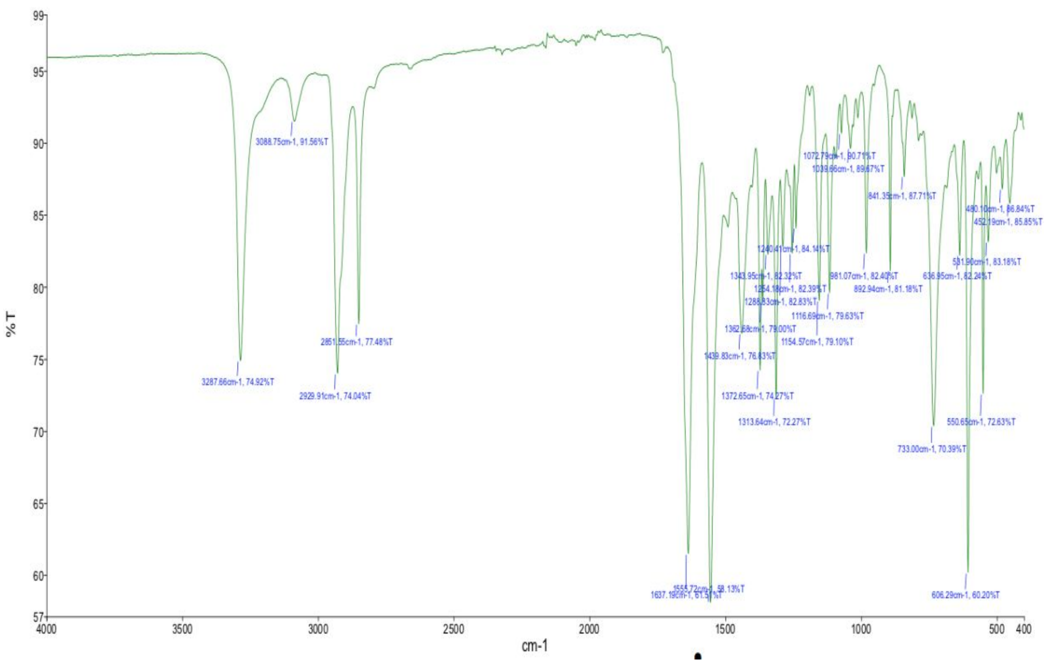

Figure S13. FTIR spectrum of compound **4**

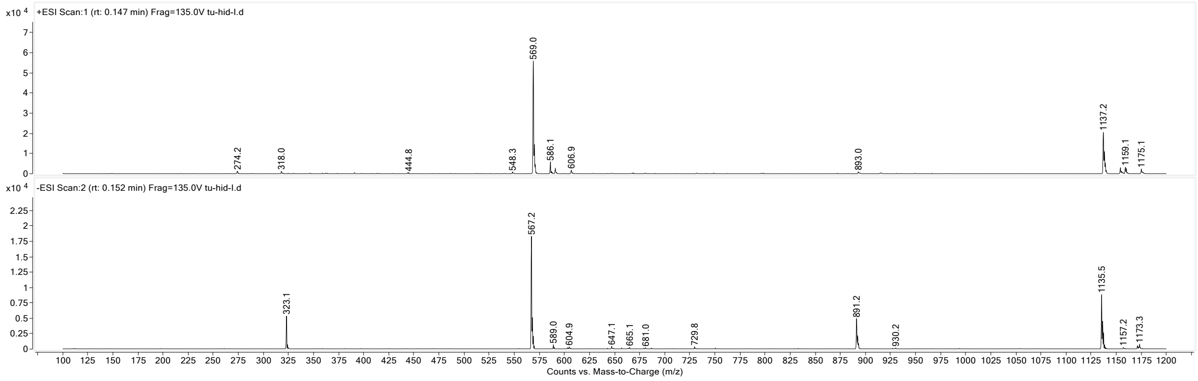

Figure S14. Mass spectrum of compound **4**

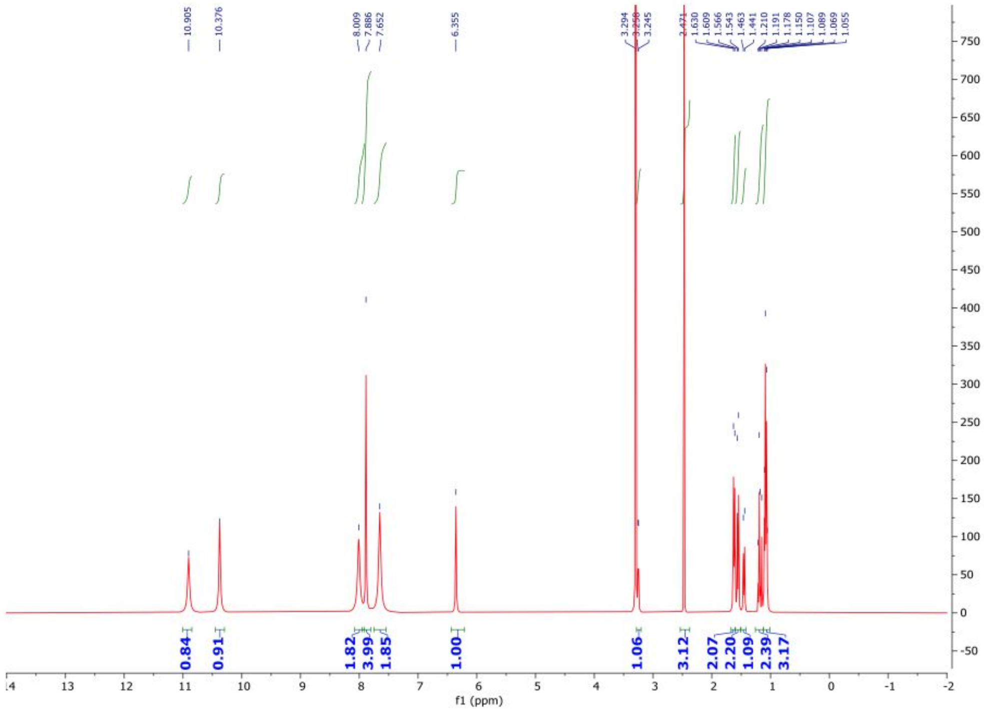

Figure S15. ^{1}H NMR spectrum of compound **4**

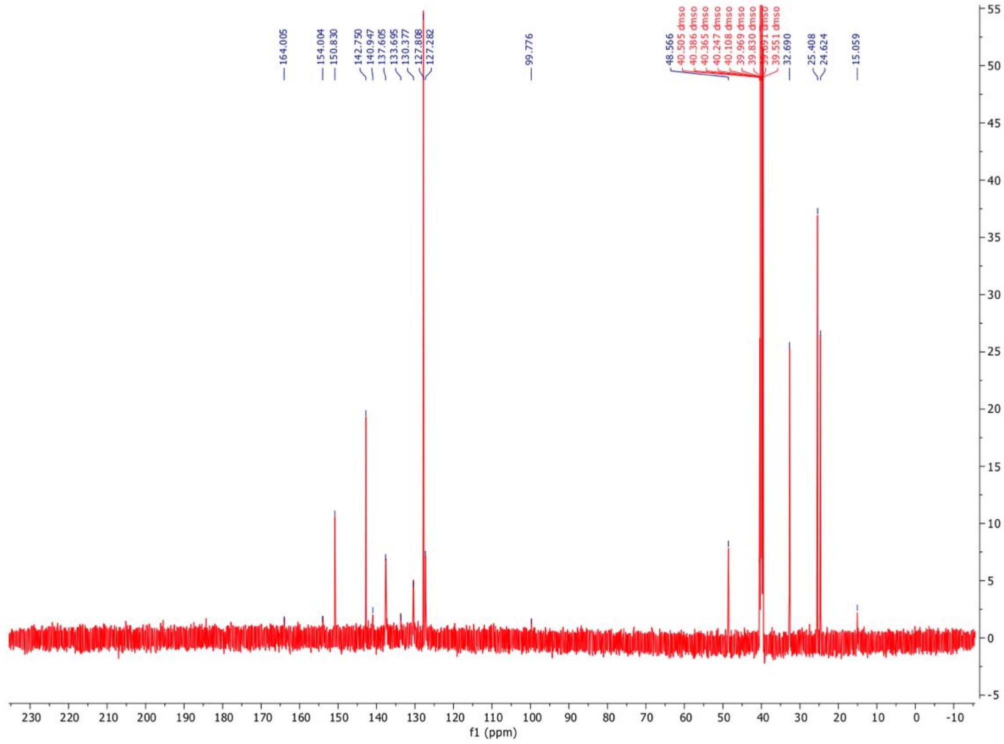

Figure S16. ^{13}C NMR spectrum of compound **4**

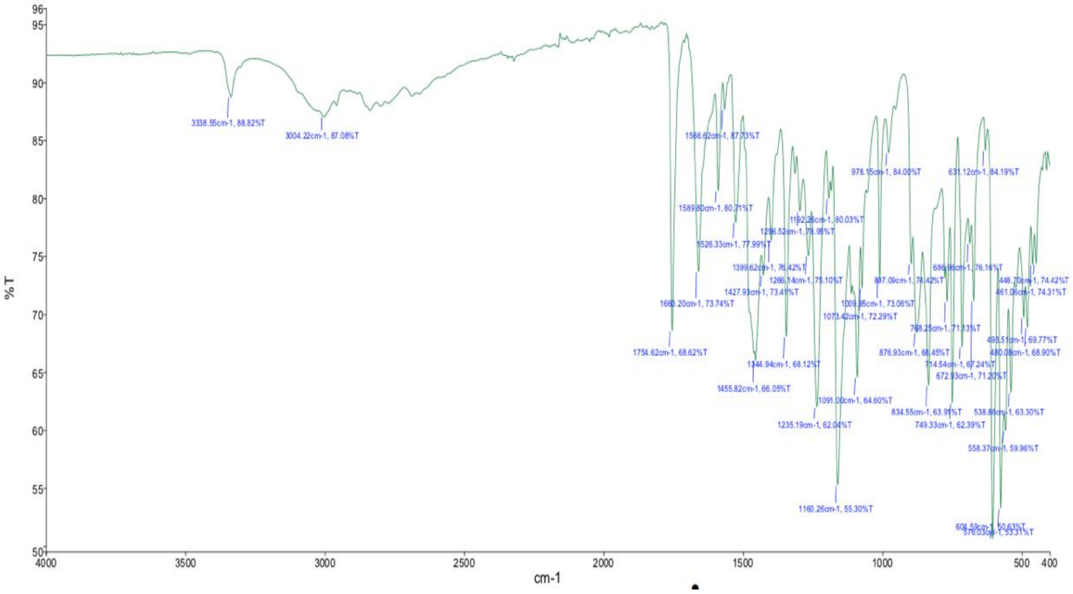

Figure S17. FTIR spectrum of compound **5**

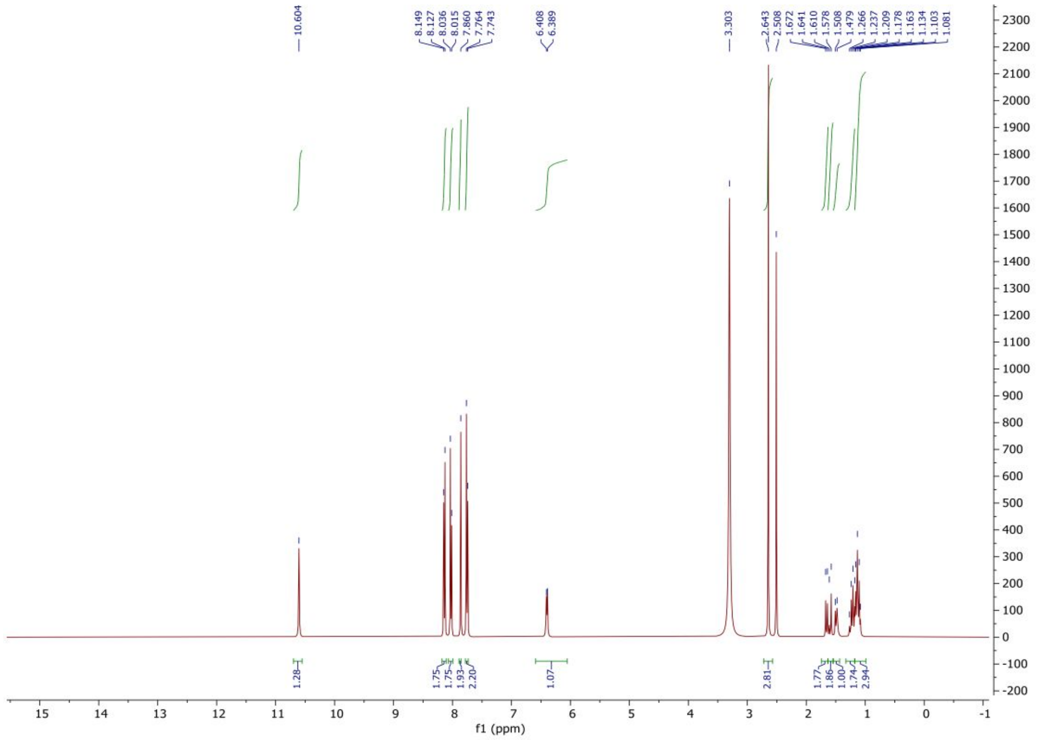

Figure S18. ^{1}H NMR spectrum of compound **5**

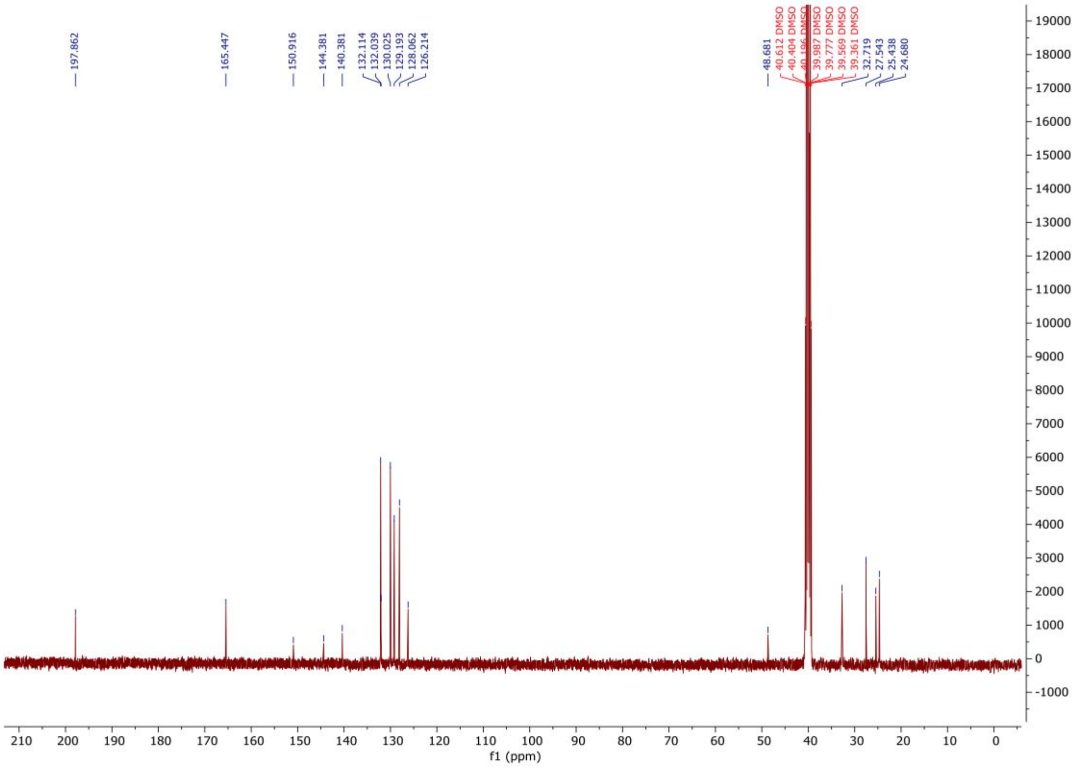

Figure S19. ^{13}C NMR spectrum of compound **5**

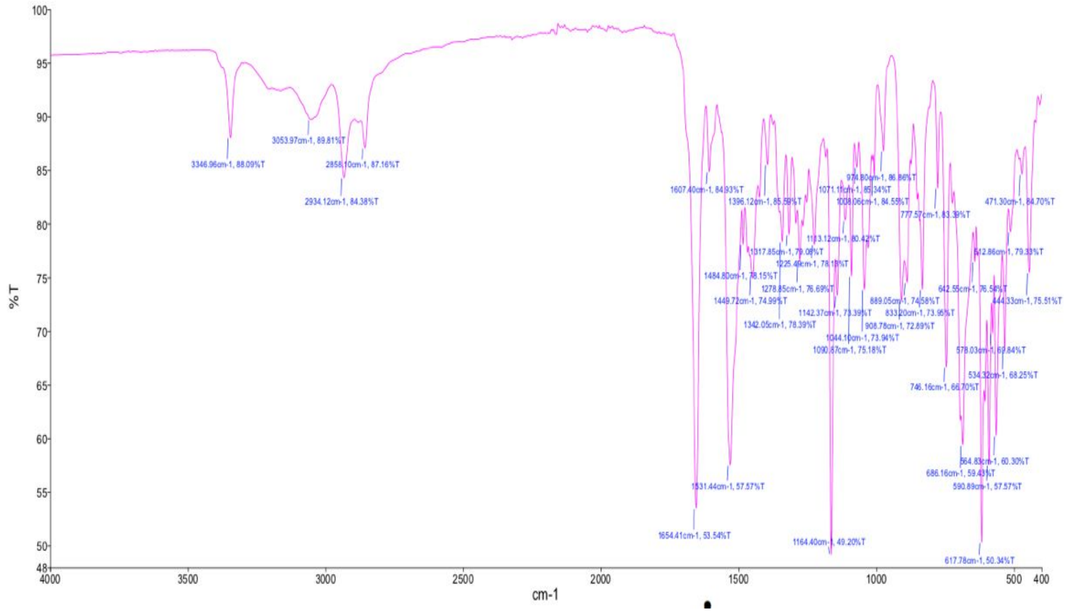

Figure S20. FTIR spectrum of compound **6**

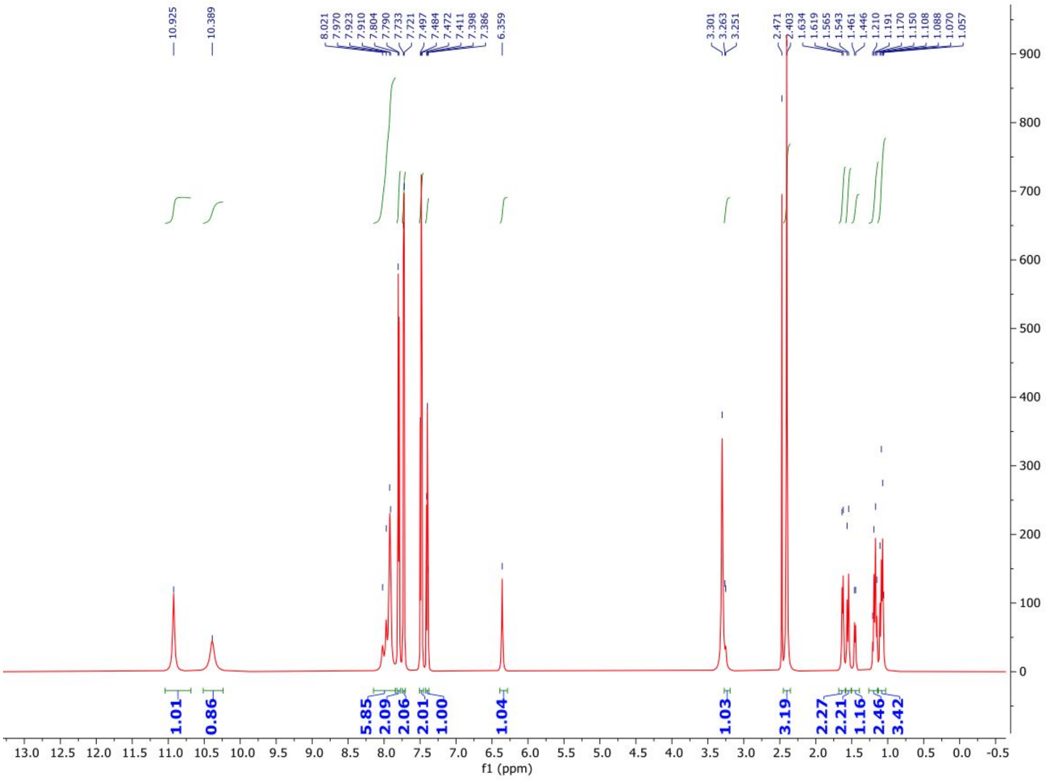

Figure S21. ^{1}H NMR spectrum of compound **6**

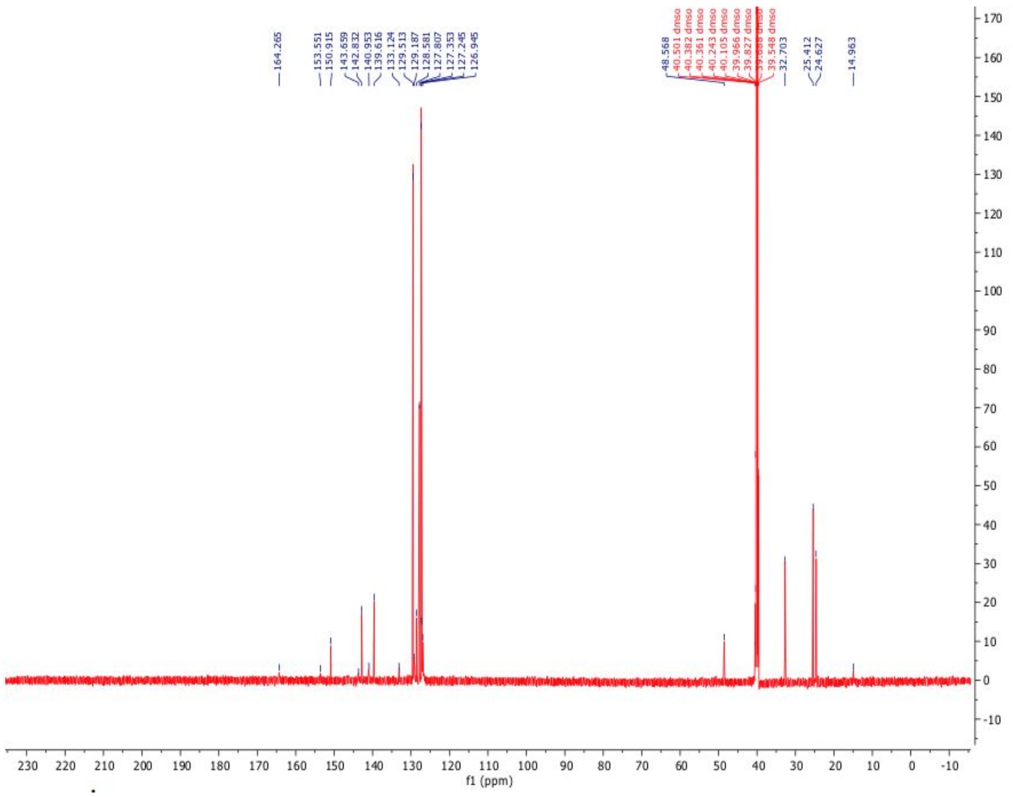

Figure S22. ^{13}C NMR spectrum of compound **6**

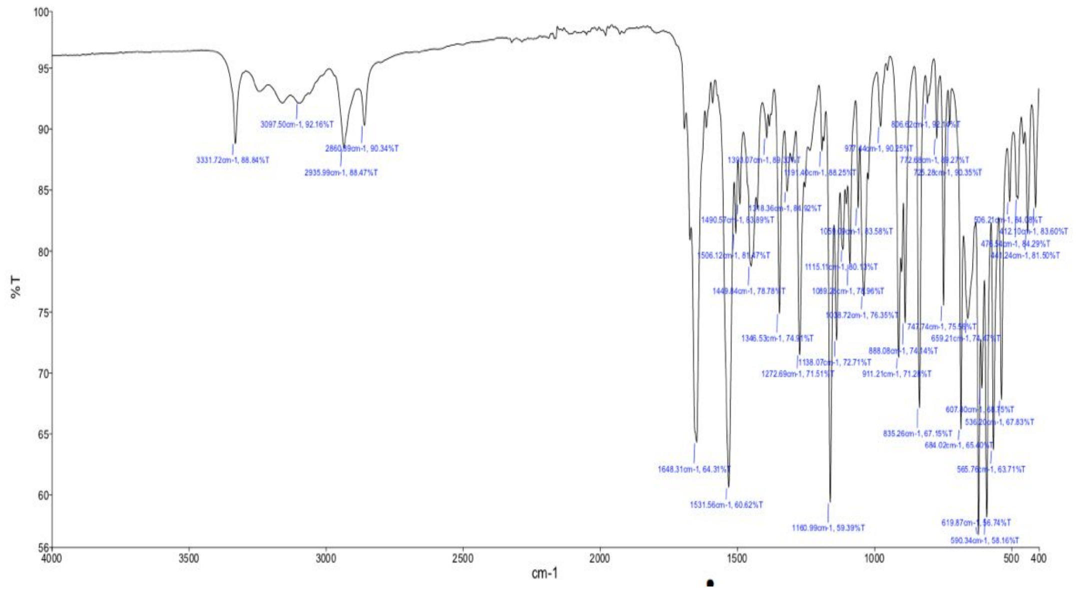

Figure S23. FTIR spectrum of compound **7**

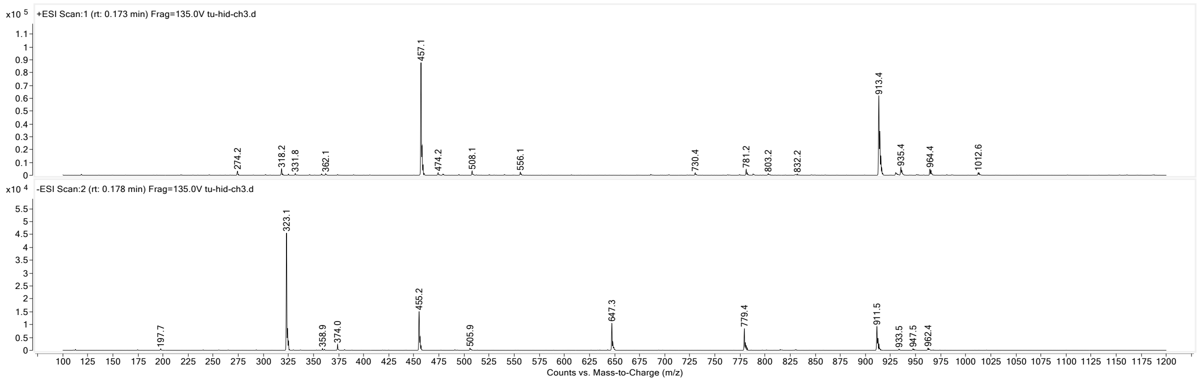

Figure S24 Mass spectrum of compound **7**

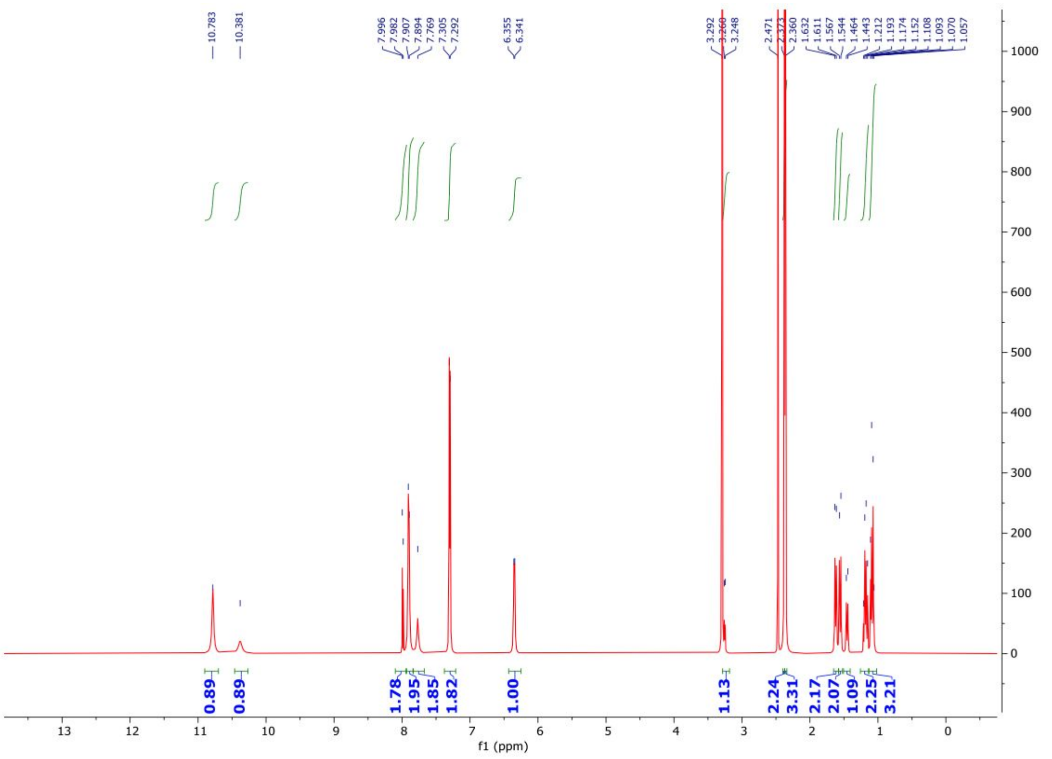

Figure S25. ^{1}H NMR spectrum of compound **7**

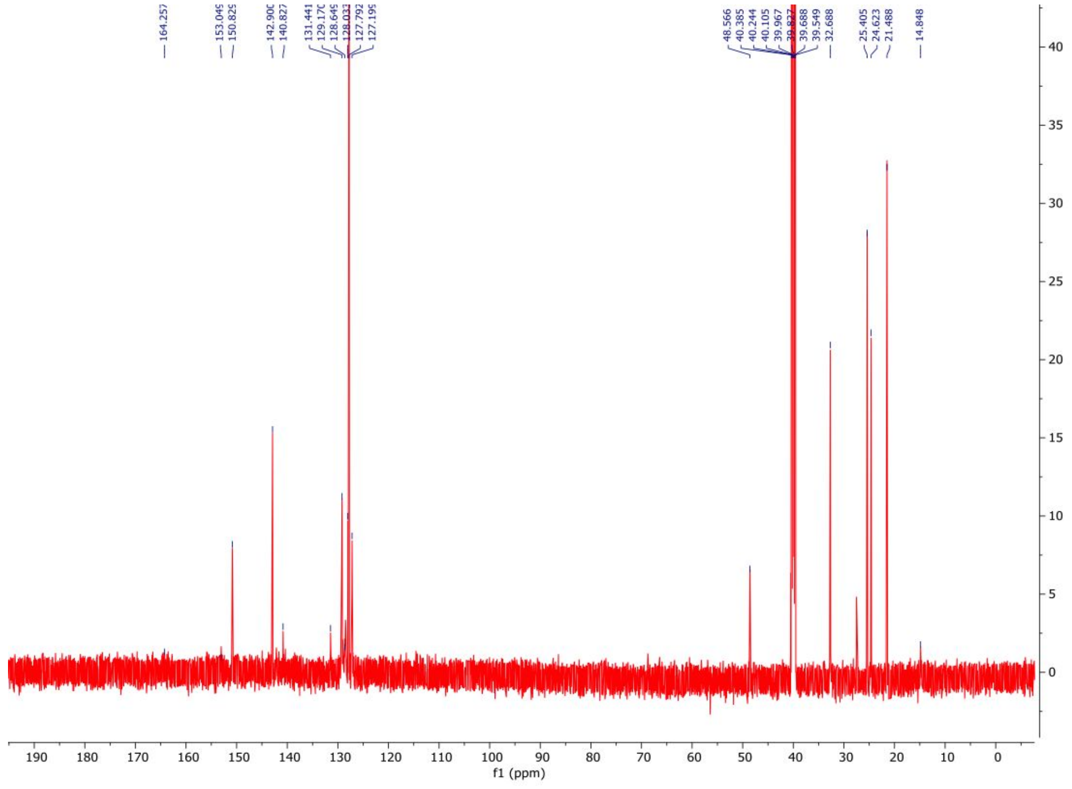

Figure S26. ^{13}C NMR spectrum of compound **7**

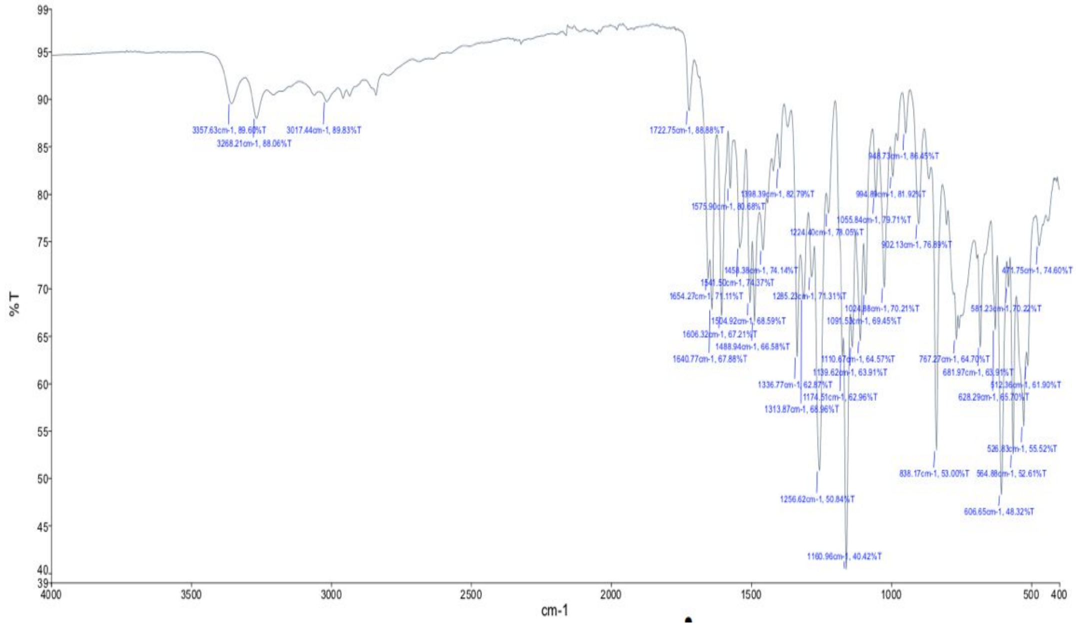

Figure S27. FTIR spectrum of compound **8**

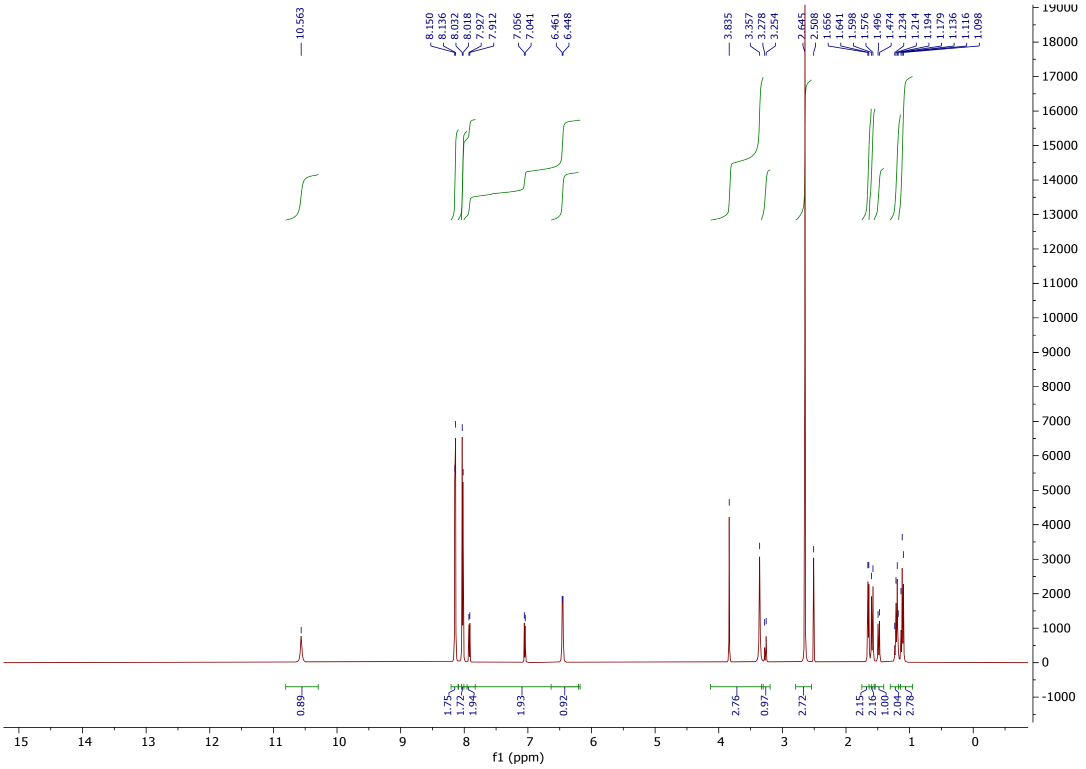

Figure S28. ^{1}H NMR spectrum of compound **8**

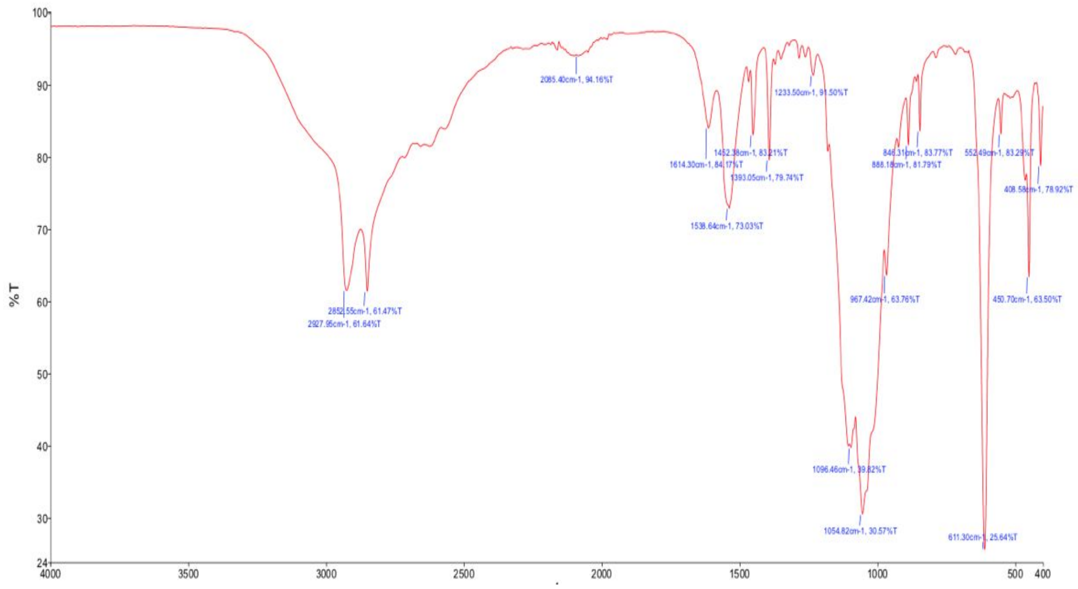

Figure S29. FTIR spectrum of compound **9**

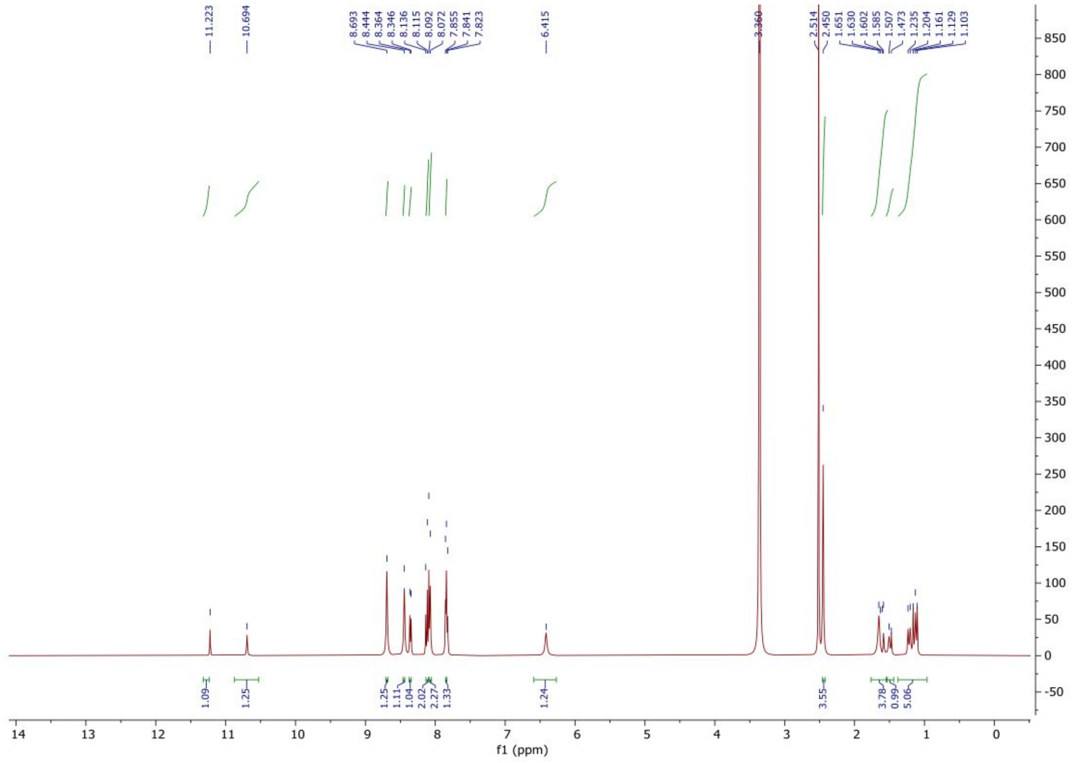

Figure S30. ^{1}H NMR spectrum of compound **9**

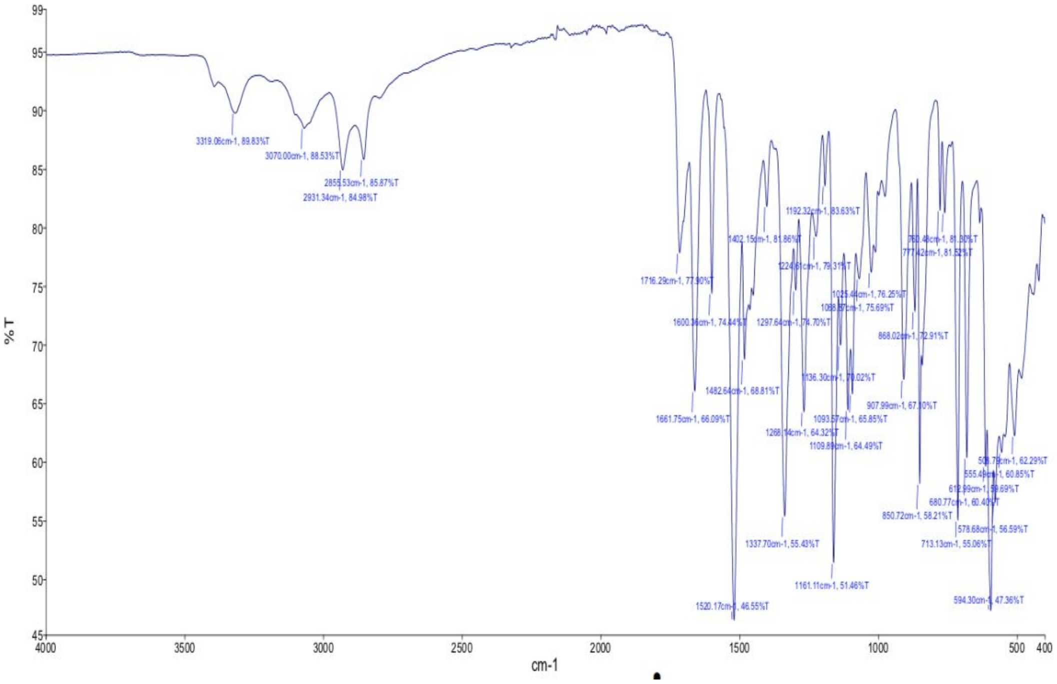

Figure S31. FTIR spectrum of compound **10**

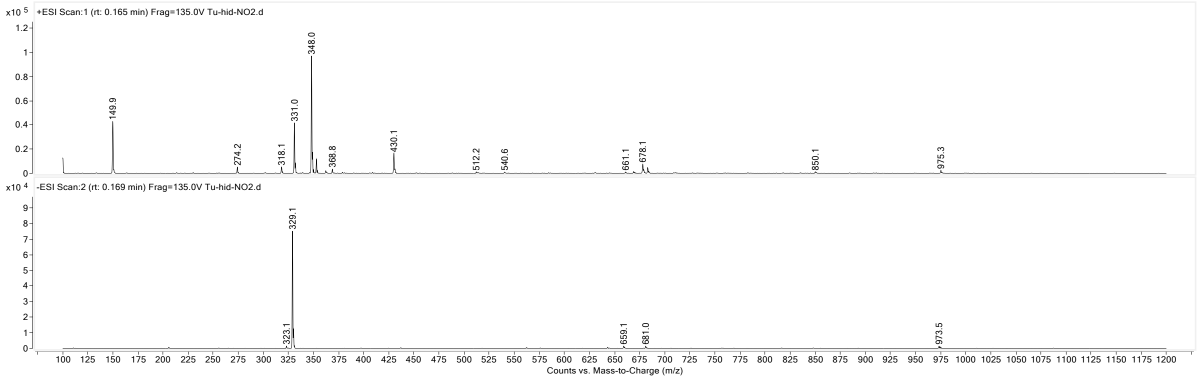

Figure S32. Mass spectrum of compound **10**

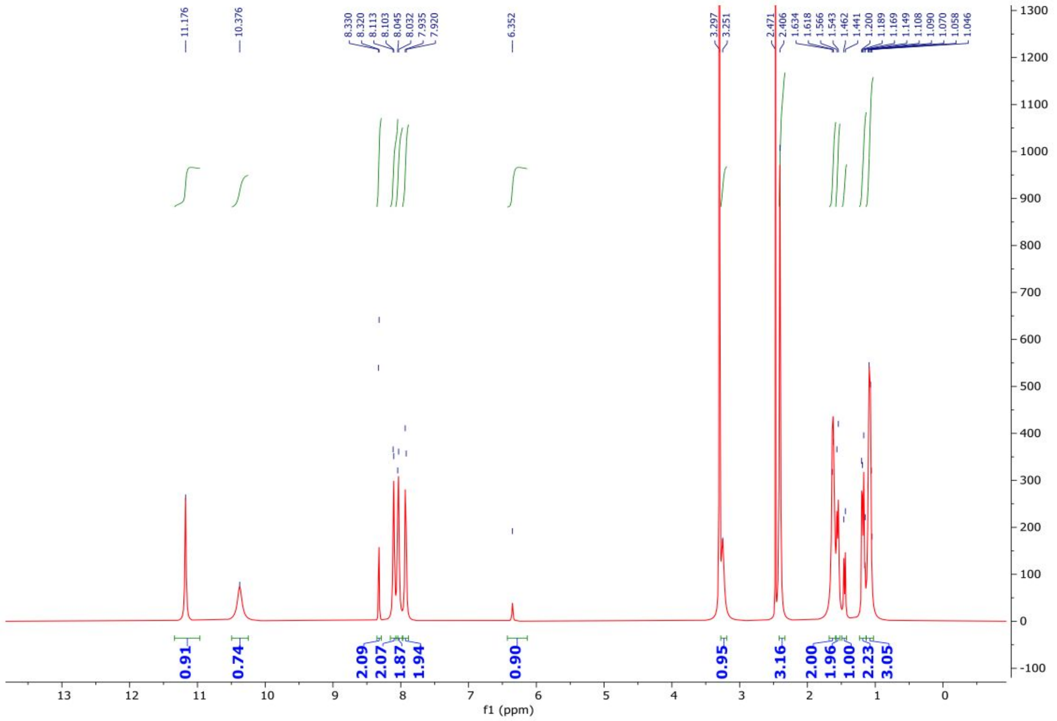

Figure S33. ^{1}H NMR spectrum of compound **10**

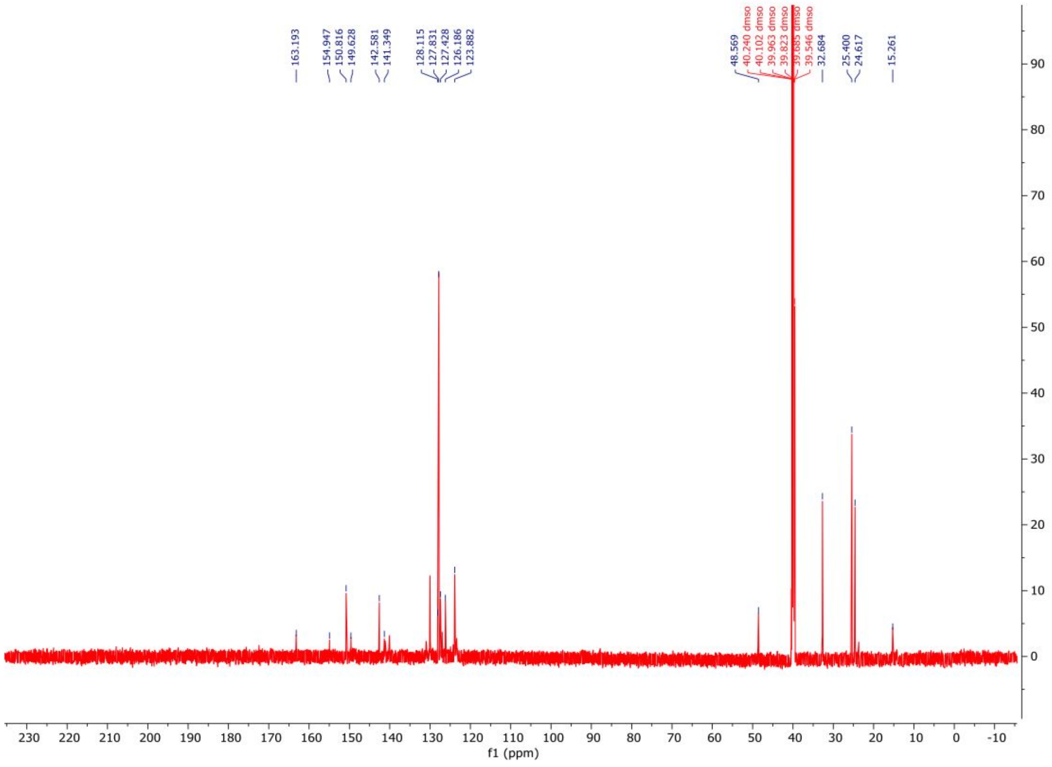

Figure S34. ^{13}C NMR spectrum of compound **10**

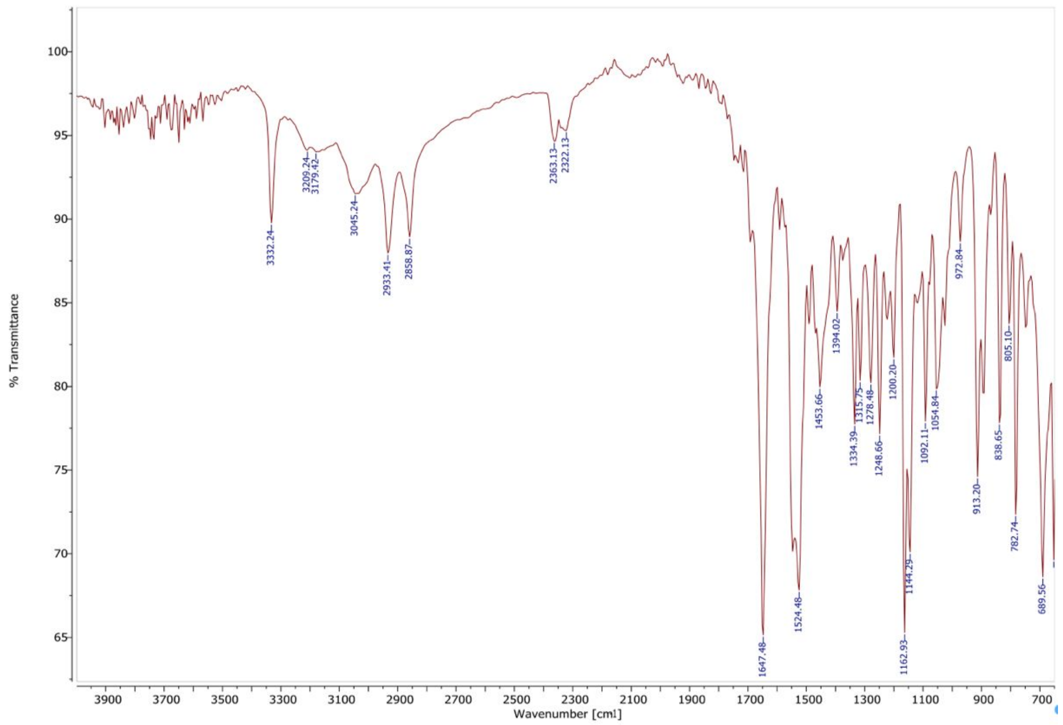

Figure S35. FTIR spectrum of compound **11**

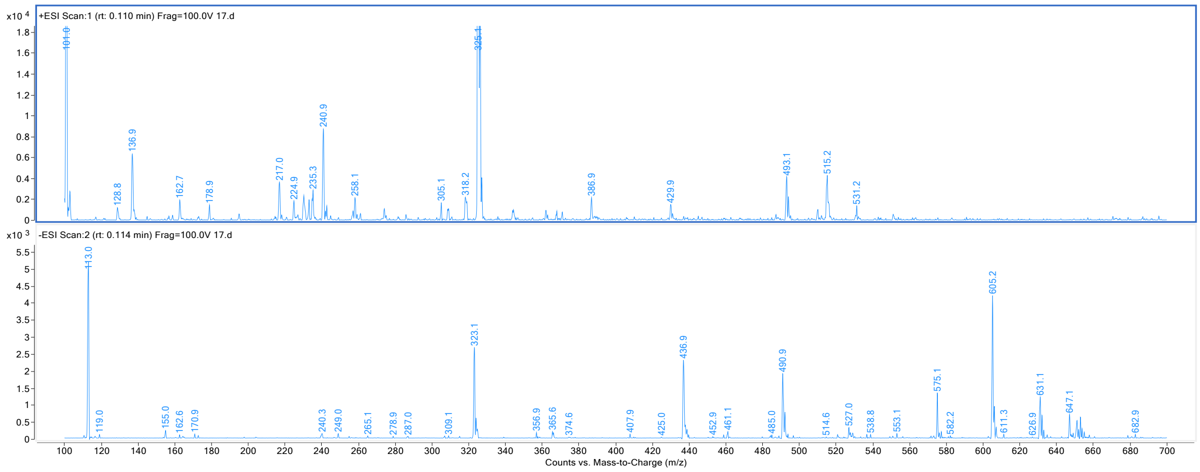

Figure S36. Mass spectrum of compound **11**

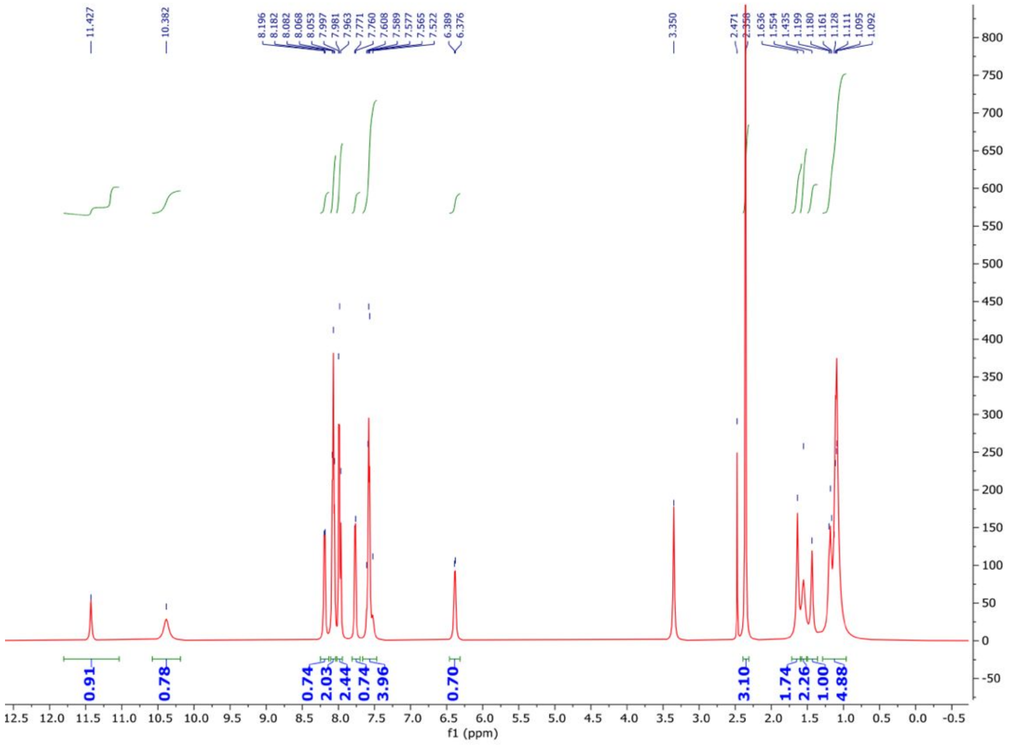

Figure S37. ^{1}H NMR spectrum of compound **11**

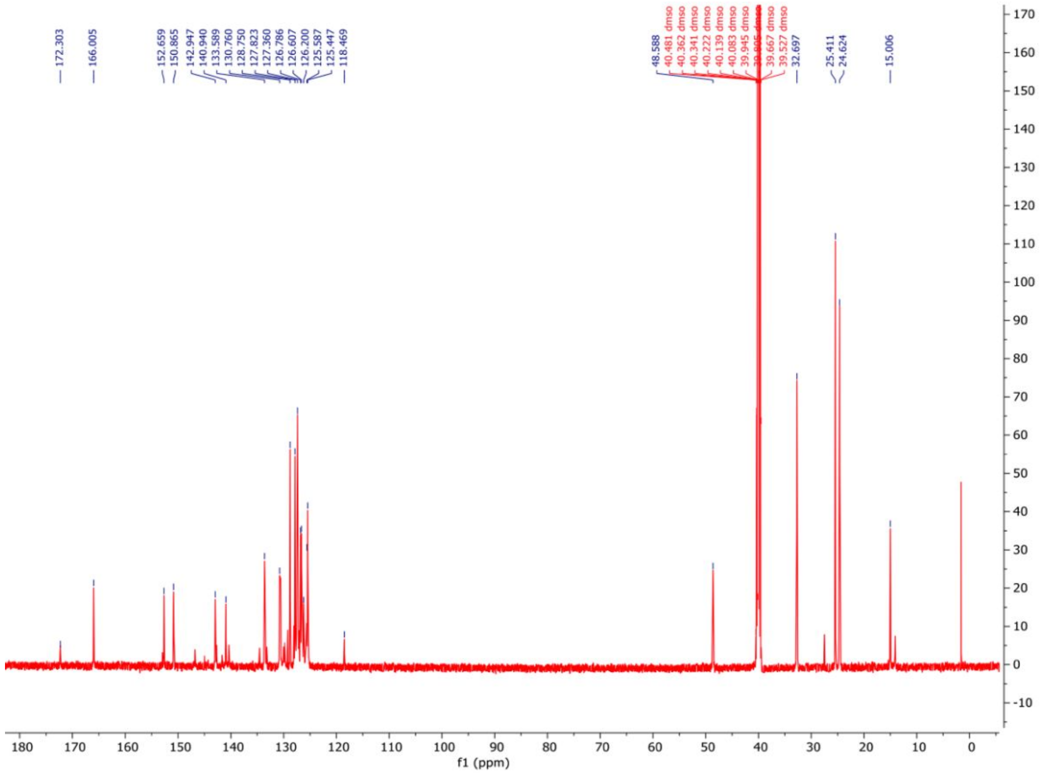

Figure S38. ^{13}C NMR spectrum of compound **11**

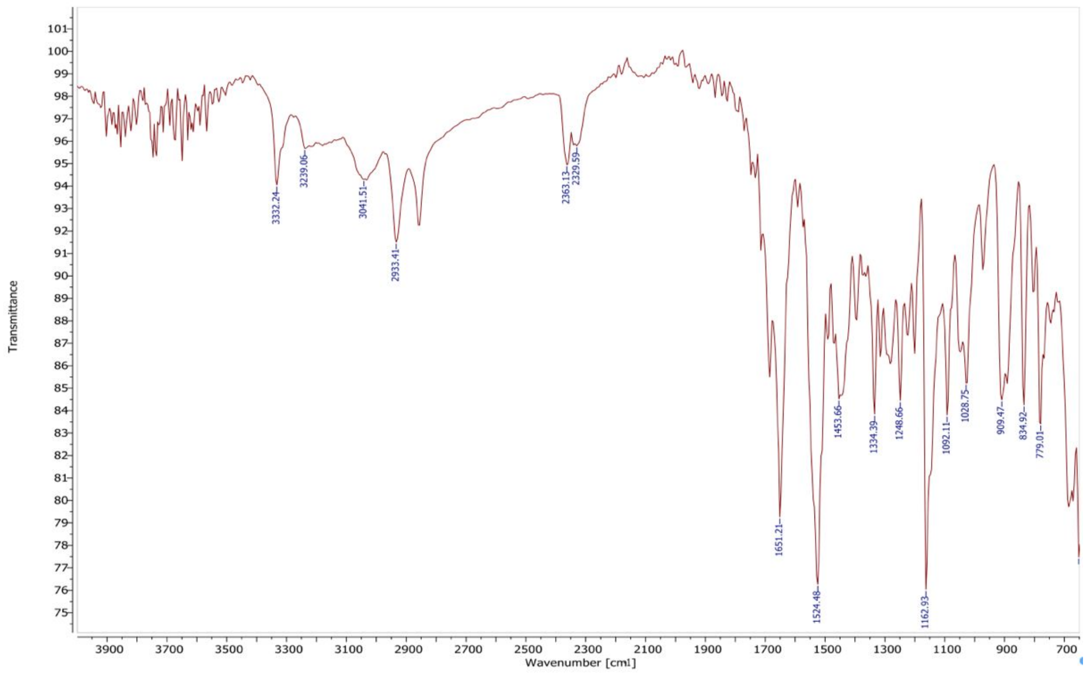

Figure S39. FTIR spectrum of compound **12**

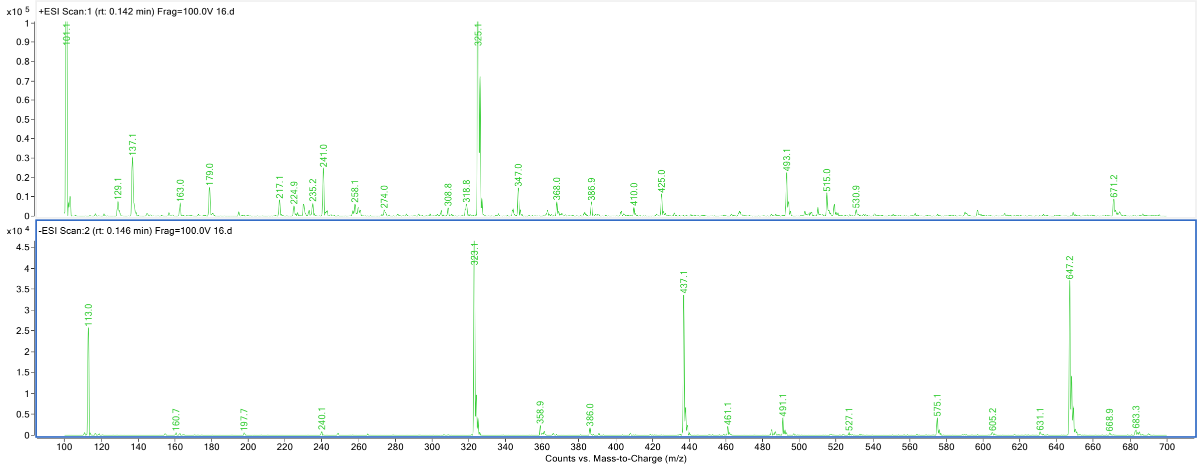

Figure S40. Mass spectrum of compound **12**

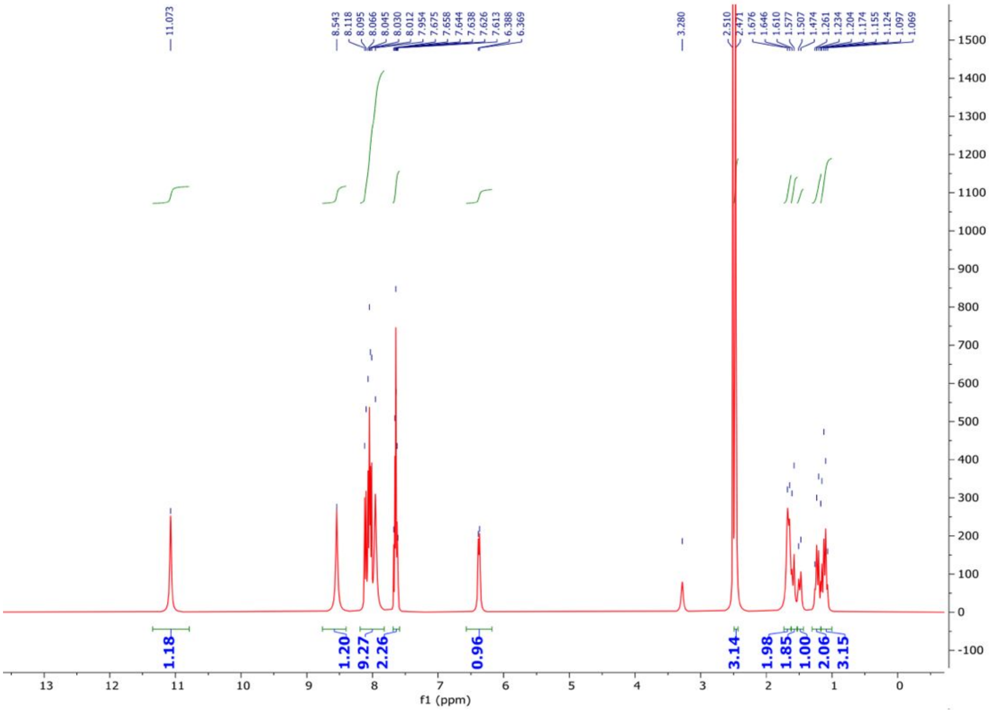

Figure S41. ^{1}H NMR spectrum of compound **12**

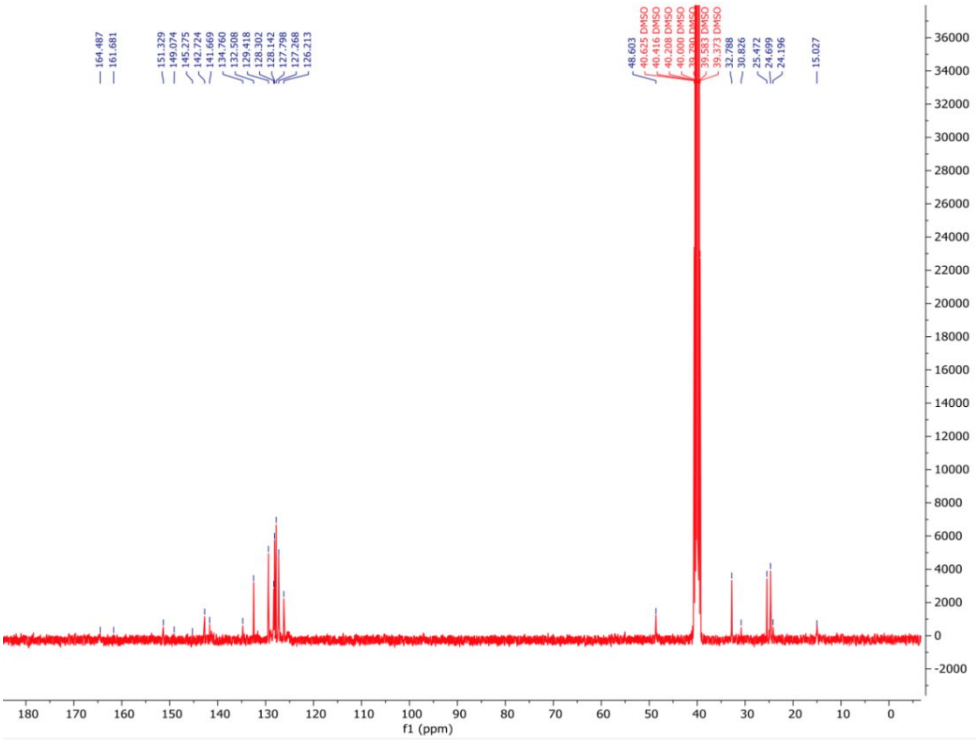

Figure S42. ^{13}C NMR spectrum of compound **12**

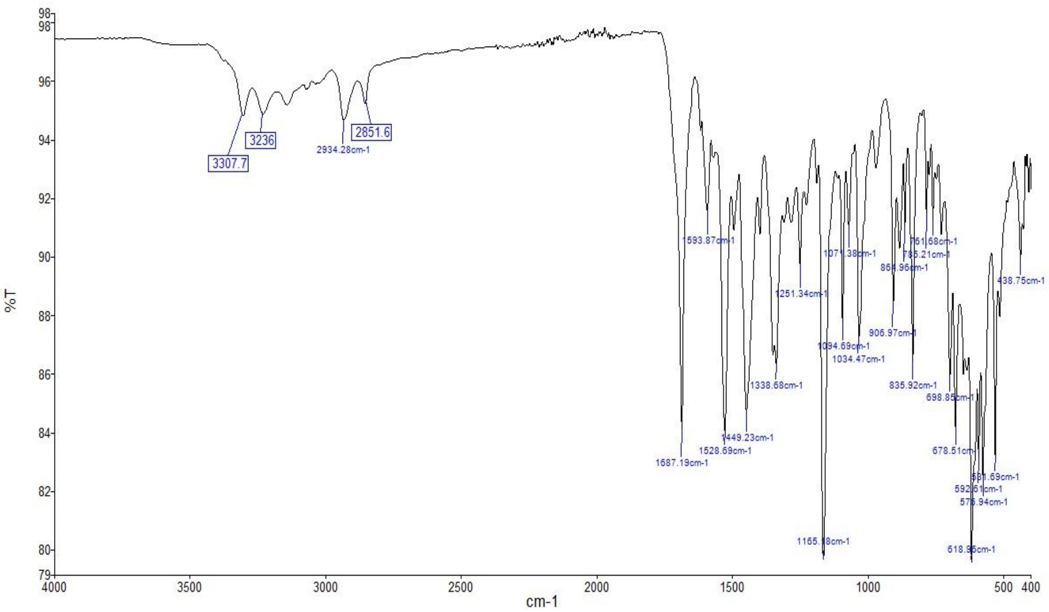

Figure S43. FTIR spectrum of compound **13**

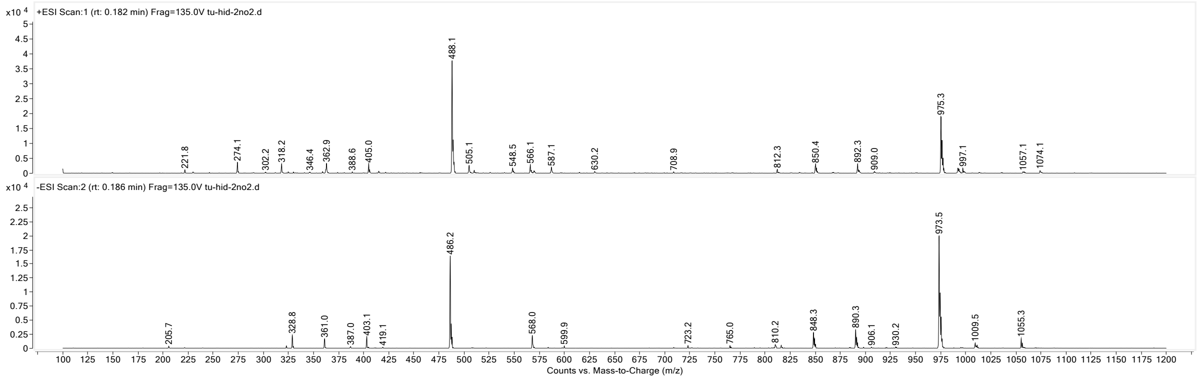

Figure S44. Mass spectrum of compound **13**

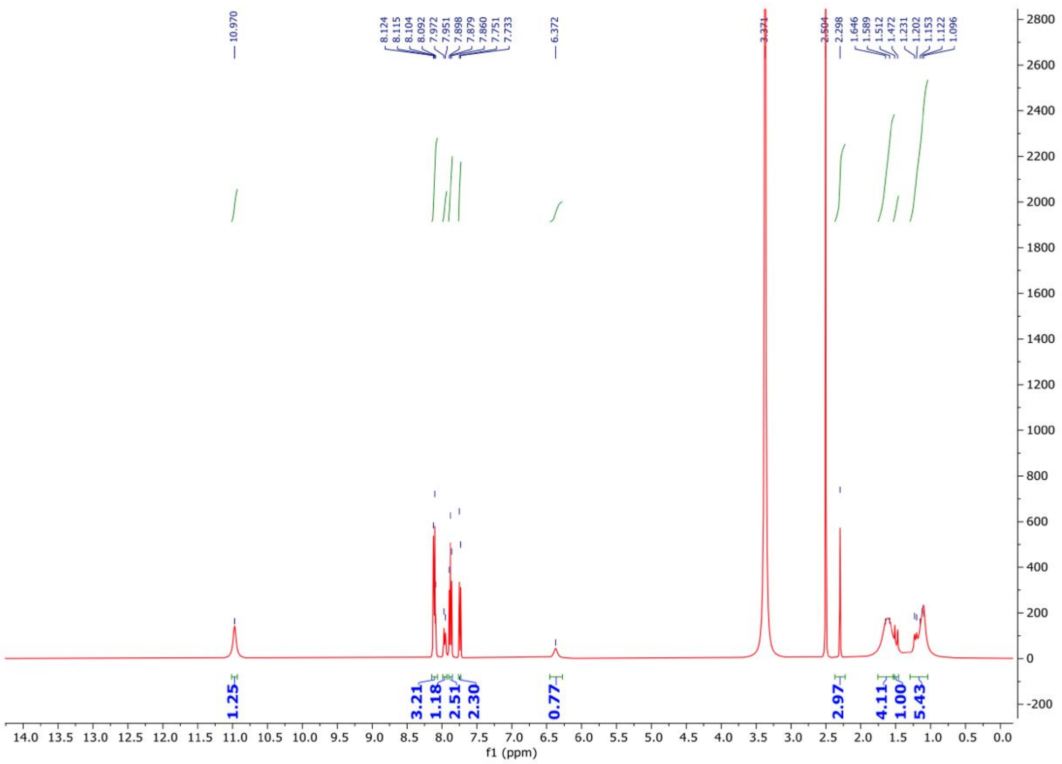

Figure S45. ^{1}H NMR spectrum of compound **13**

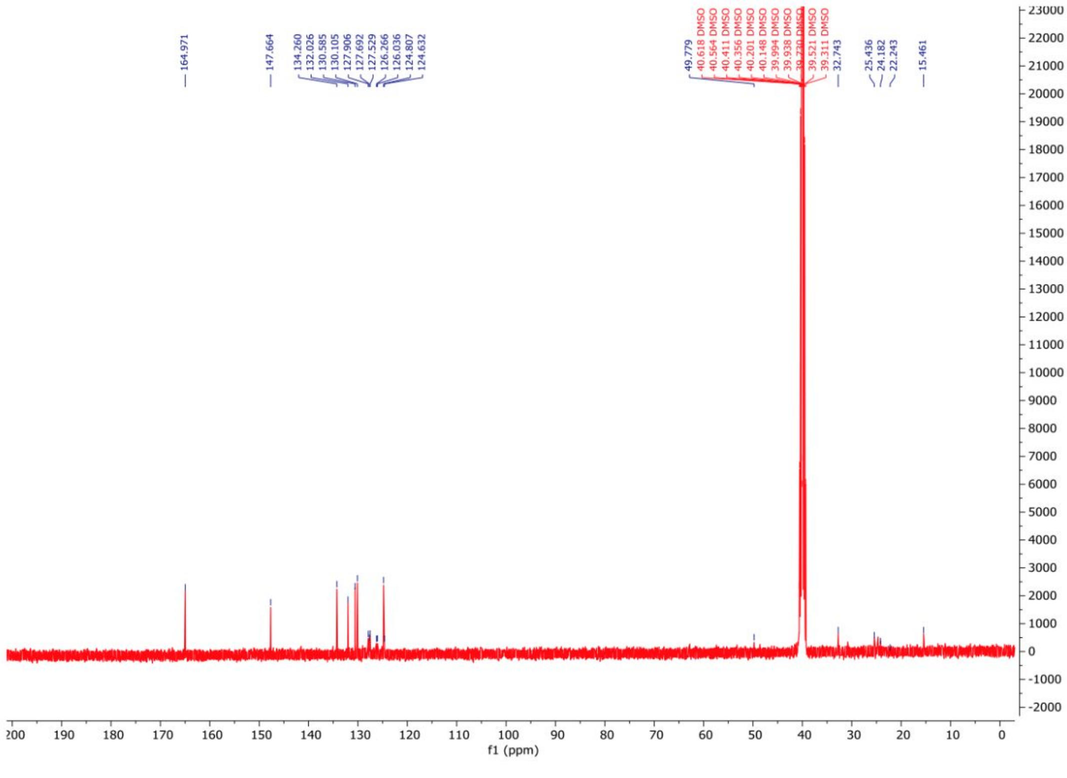

Figure S46. ^{13}C NMR spectrum of compound **13**

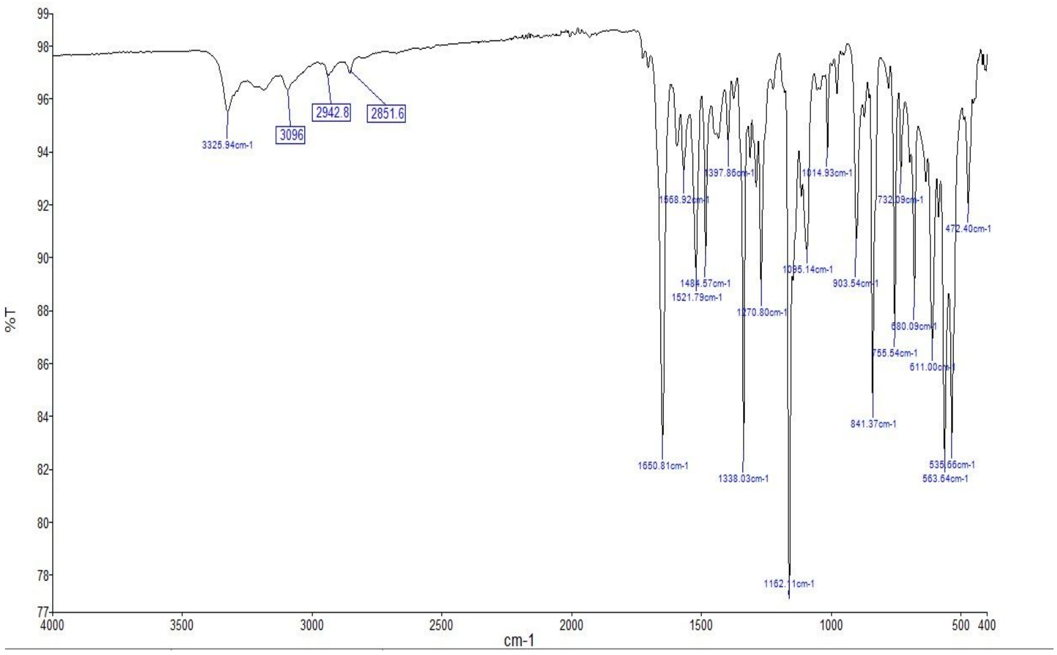

Figure S47. FTIR spectrum of compound **14**

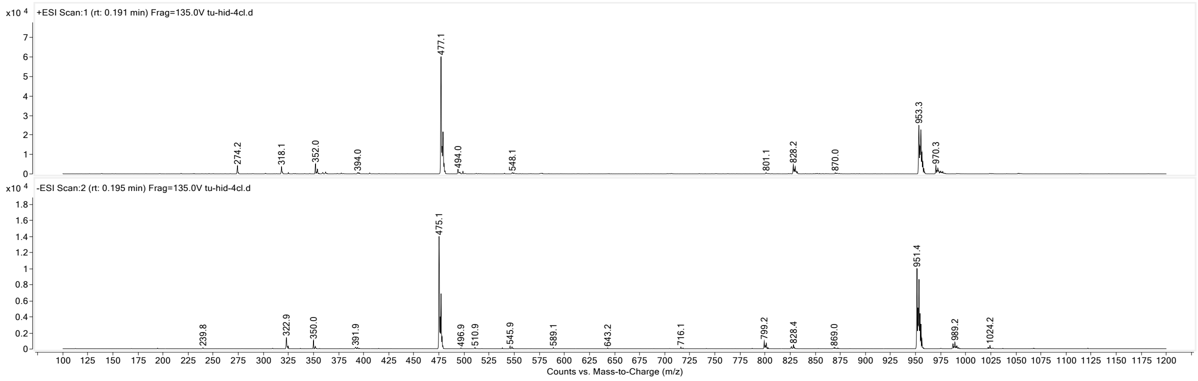

Figure S48. Mass spectrum of compound **14**

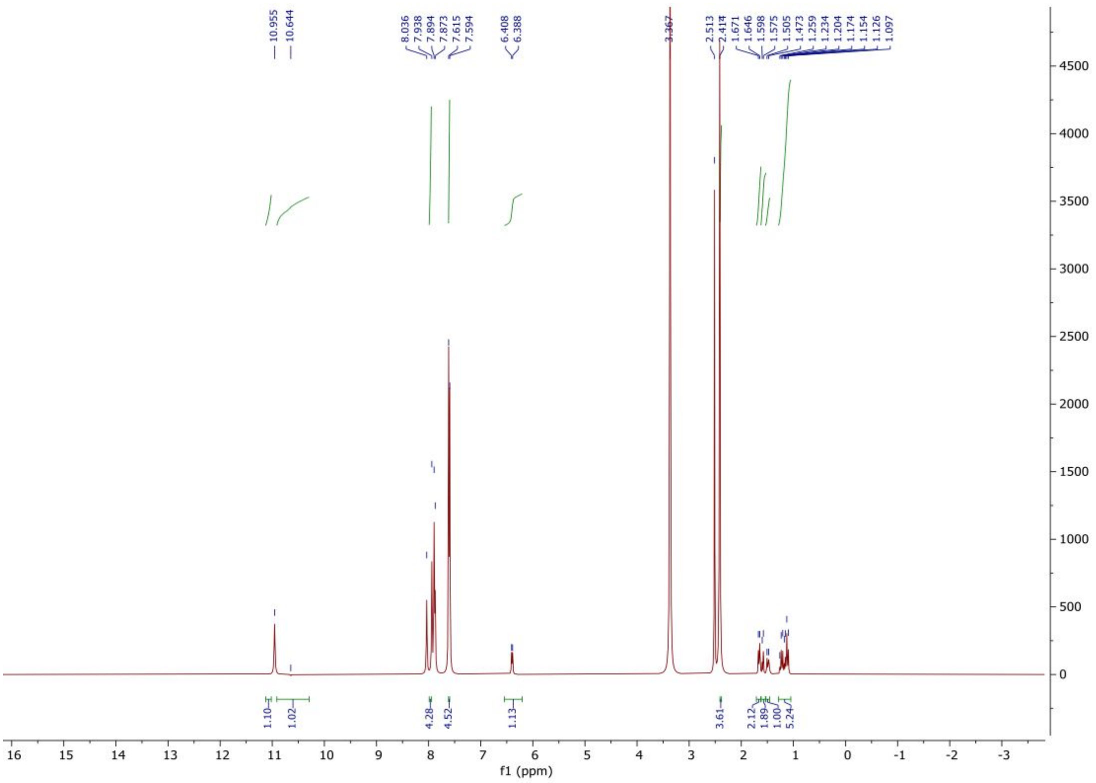

Figure S49. ^{1}H NMR spectrum of compound **14**

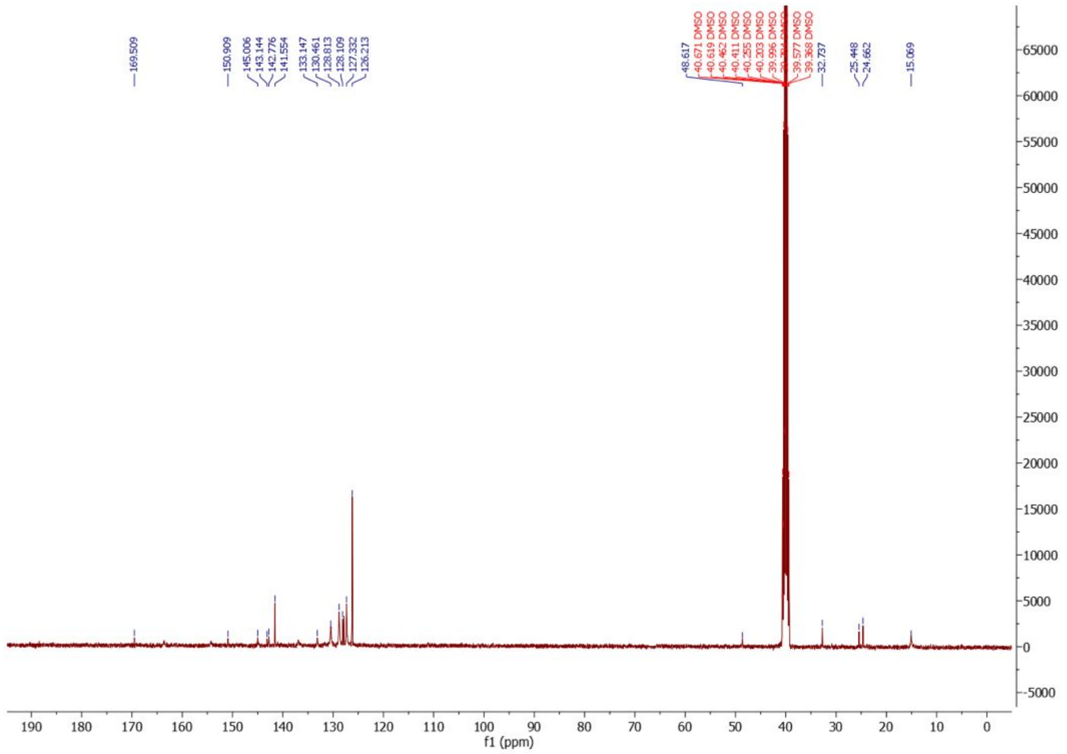

Figure S50. ^{13}C NMR spectrum of compound **14**
